# Thiazoles inhibit reactive oxygen and nitrogen species mediated diabetes mellitus: integrated *in vitro* and *in silico* insights

**DOI:** 10.1039/d6ra01667g

**Published:** 2026-07-02

**Authors:** Hamid Aziz

**Affiliations:** a Department of Chemistry, Quaid-I-Azam University Islamabad-45320 Pakistan hamidazizwazir@gmail.com

## Abstract

Thiazole heterocycles, characterized by their unique combination of sulfur and nitrogen atoms, have emerged as versatile scaffolds in medicinal chemistry. The presence of heteroatoms provide multiple binding sites, enabling rapid investigation of structure activity relationships (SARs), aiding the development of potent, and targeted inhibitors. Thiazoles exhibit remarkable tolerance to diverse functionalities, and can be readily modified to fine-tune lipophilicity, polarity, and metabolic stability, making thiazoles as valuable frameworks for hit-to-lead optimization, and drug development with favorable pharmacokinetic profiles. In this context, the current review (2020–2025) highlights: biologically inspired thiazoles, and approved drugs, synthetic approaches like condensation, multicomponent reactions, microwave-assisted synthesis, and cycloaddition strategies, recent advances in anti-oxidant, and antidiabetic thiazole analogues, analysis of SARs, and molecular docking insights into protein-ligand interactions. Thus, biological screenings reveal thiazole hybrids often outperform simple thiazoles, with several analogues demonstrating superior anti-oxidant and antidiabetic potential to drugs in free radical scavenging and enzymes inhibition assays. SARs analysis confirm electron-donating and electron-withdrawing groups around thiazole ring significantly influence inhibitory potential of the screened analogues. Molecular docking further supports these findings, showing strong intermolecular interactions that underpin enhanced bioactivity. To conclude, thiazole scaffolds represent a promising frontier in rational drug design, and discovery. Thus, the present review article emphasizes continued exploration through optimized synthetic methodologies, hybrid development, and comprehensive biological evaluation to unlock their full therapeutic potential.

## Abstract 2

Thiazole heterocycles, characterized by their unique combination of sulfur and nitrogen atoms, have emerged as versatile scaffolds in medicinal chemistry.

## Graphical abstract

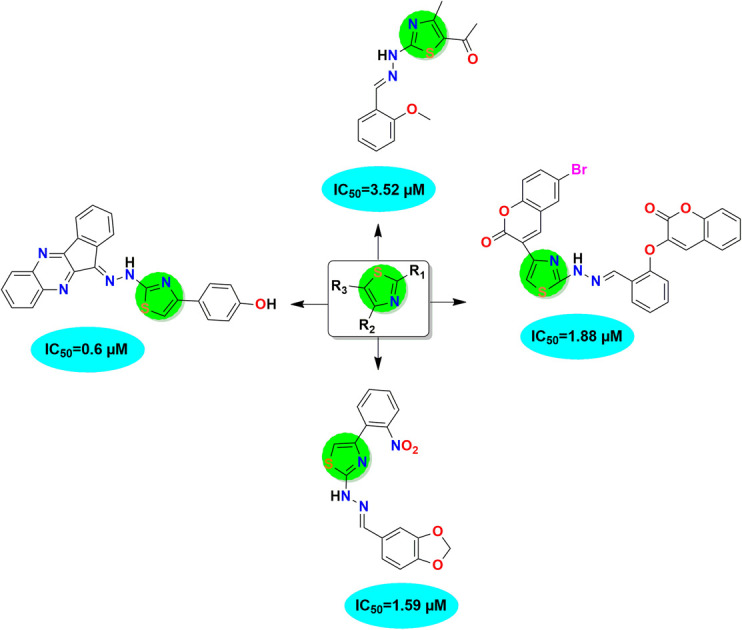

## 1 Introduction

The rapid evolution of contemporary lifestyles, and dietary patterns has established metabolic syndrome-defined by a constellation of interconnected risk factors including abdominal obesity, hypertension, and hyperglycemia-a significant public health burden.^1^ Within this context, Diabetes Mellitus (DM) presents as a prototypical chronic, lifestyle-associated disease, and remains among the top ten leading causes of mortality worldwide.^1^ DM pathogenesis involves either a quantitative defect in insulin secretion from pancreatic β-cells or impaired cellular insulin sensitivity, culminating in persistent hyperglycemia. The clinical presentation of DM is marked by hallmark symptoms: polyuria, polydipsia, polyphagia, unintended weight loss, fatigue, and elevated postprandial glucose levels. Sustained chronic hyperglycemia initiates a cascade of deleterious microvascular, and macrovascular complications. Microvascular insults typically manifest retinopathy, and nephropathy, while macrovascular involvement significantly elevates the risk of cardiovascular disease. DM frequently coexists with broader metabolic dysregulations, such as obesity, dyslipidemia, accelerated atherosclerosis, and adverse gestational changes. These comorbidities, particularly cardiovascular events, renal failure, neuropathy, and recurrent infections, represent the primary drivers of increased morbidity, and mortality within diabetic populations. The collective burden of these complications significantly complicates disease management strategies, hastens clinical progression, and substantially elevates the overall risk of mortality.^2^

Human body naturally generates reactive oxygen, and nitrogen species (RONS) as byproducts of normal metabolic processes, where they play essential roles in cell signaling, immune defense, and homeostasis. However, RONS are inherently unstable, short-lived, and highly reactive, posing a threat to cellular integrity when produced in excess. An overabundance of RONS leads to oxidative stress-a pathological condition characterized by modification, and damage of vital macromolecules, including proteins, lipids, and nucleic acids. This oxidative burden disrupts cellular redox homeostasis, and initiates harmful processes such as lipid peroxidation, and protein oxidation, ultimately impairing cellular function, and viability.^3^ Oxidative stress plays a pivotal role in the onset and progression of Type 2 diabetes mellitus, through impaired pancreatic β-cell function, and induction of insulin resistance. Furthermore, oxidative stress significantly contributes to the development of both microvascular, and macrovascular complications associated with DM. The excessive generation of RONS under hyperglycemic conditions leads to cellular damage, and chronic inflammation, which underpins progression of microvascular, macrovascular, and other systemic complications. Such complications are exacerbated by persistent oxidative environment, which disrupts homeostasis, promotes thrombosis, and degenerate tissues. Therefore, targeting oxidative stress remains a promising therapeutic strategy to mitigate systemic impact of DM.^4^

### 1.1. Prevalence of DM

According to World Health Organization (WHO), global burden of diabetes has risen sharply over the past three decades. In 2022, an estimated 14% of adults aged 18 years and older were living with diabetes, compared to only 7% in 1990. Alarmingly, more than half (59%) of adults aged 30 years, and above with diabetes were not receiving medication in 2022, with treatment coverage lowest in low- and middle-income countries. In 2021, diabetes directly caused 1.6 million deaths, nearly half (47%) of which occurred before the age of 70 years. In addition, diabetes was responsible for 530 000 kidney disease deaths, while high blood glucose contributed to approximately 11% of cardiovascular deaths. Since 2000, mortality rates attributable to diabetes have continued to rise. In contrast to the overall decline in mortality from the four major noncommunicable diseases (cardiovascular diseases, cancer, chronic respiratory diseases, and diabetes) between the ages of 30, and 70, which decreased globally by 20% between 2000, and 2019. The mentioned trends underscore the urgent need for improved prevention, treatment coverage, and global health strategies to address diabetes, and the related complications.^1^

• The global prevalence of DM has escalated dramatically, rising from approximately 200 million cases in 1990 to 830 million in 2022, underscoring the rapid expansion of this noncommunicable disease worldwide.

• In 2022, more than half of individuals living with DM were not receiving medication, reflecting substantial gaps in treatment coverage, and access to care worldwide.

• In 2021, diabetes, and diabetes-related kidney disease together accounted for more than 2 million deaths worldwide, underscoring the severe impact of poor glycemic control on global mortality.

• Elevated blood glucose is a major cardiovascular risk factor, accounting for nearly 11% of all cardiovascular deaths worldwide, thereby underscoring the systemic impact of diabetes beyond glycemic control.

• Evidence demonstrates that adopting a healthy diet, engaging in regular physical activity, maintaining normal body weight, avoiding tobacco use, and adhering to appropriate medication regimens can effectively prevent or delay the onset of Type 2 diabetes mellitus.^1^

International Diabetes Federation (IDF), Diabetes Atlas (11^th^ Edition), reports an adult prevalence of 11.1% (20–79 years), equivalent to ∼589 million (1 in 9) people in 2024, with projections of ∼853 million by 2050 (46% increase). Over 90% of diabetes diagnoses are for Type 2 diabetes mellitus, and remarkably, over 40% (4 in 10) of individuals living with the disease are completely unaware they have it. DM caused 3.4 million deaths in 2024 (1 every 9 seconds), and generated at least USD 1 trillion in health expenditure ∼338% increase over the past 17 years. The concentration of cases in low- and middle-income countries (∼81%) amplifies both mortality, and financial strain on vulnerable health systems.^5^ According to IDF Diabetes Atlas (11^th^ edition), Pakistan currently has the world's highest age-standardized diabetes prevalence among adults aged 20–79 years at 31.4%, with approximately 34.5 million adults living with diabetes in 2024, while China has the largest absolute number of diabetes cases globally at 148.0 million, followed by India with 89.8 million, and the United States of America with 38.5 million affected adults. More importantly, IDF projections indicate that Pakistan is expected to surpass United States by 2050 in total number of adults living with diabetes, rising to an estimated 70.2 million cases compared with 43.0 million in USA, highlighting the rapidly escalating diabetes burden in Pakistan.^5^

### 1.2. Classification of DM

DM is associated with increase in RONS, downregulation of polyol pathway, advanced glycation end-product, protein kinase C activation, and hexosamine pathway flux. Based on pathogenesis, DM is classified into Type 1 diabetes mellitus (T1DM) or insulin-dependent DM (IDDM), Type 2 diabetes mellitus (T2DM) or non-insulin dependent DM (NIDDM), and Gestational diabetes (GD).^2^

#### 1.2.1. Type 1 diabetes mellitus

T1DM is an autoimmune condition in which the body's immune system mistakenly attacks, and destroys insulin-producing β-cells within the pancreas. This form of diabetes represents a smaller proportion of total diabetes cases, making up approximately 5–10% of all diagnoses. T1DM is mostly diagnosed with young people. Daily dosage of insulin control optimal blood sugar level. T1DM arises from intricate interaction involving both environmental triggers, and a person's inherent genetic susceptibility, and cannot be controlled. Common indicators of T1DM include increased thirst, urination, losing weight without trying, visual disturbances, and chronic fatigue. TIDM is managed by regular insulin therapy.^2,6^

#### 1.2.2. Type 2 diabetes mellitus

T2DM, known as adult-onset or non-insulin-dependent diabetes, contributes to 85–90% of diabetes among individuals over 45 years. T2DM is caused by dysfunctions of metabolic system, and hormones, as well as physiological disturbances including insulin resistance, and progressive decline in β-cell function. In T2DM, body's cells develop resistance to insulin, resulting in elevated blood sugar levels, although adequate amount of insulin is produced by pancreas. Several lifestyle related factors primarily lead to insulin resistance, including obesity, lack of physical exercise, smoking, and high-glycemic-index diet. Moreover, mental health conditions like depression, and anxiety can lead to T2DM, which subsequently affects social life, often in conjunction with metabolic, and cardiovascular diseases.^2,6^

#### 1.2.3. Diabetes mellitus in pregnancy

DM that arises during pregnancy affects both mother, and developing fetus. DM in pregnancy is classified into pregestational diabetes mellitus (PGDM), which is present before conception, and gestational diabetes mellitus (GDM), which arises during pregnancy. PGDM encompasses both insulin-dependent, and non-insulin-dependent DM. GDM develops during pregnancy, and is common among expectant mothers.^2,6^

### 1.3. Therapy in DM

**Fig. 1:**
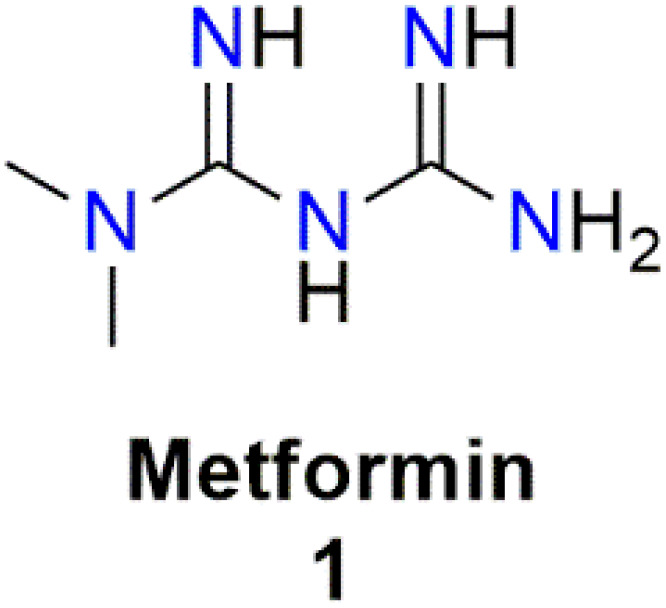
Structure of Biguanide-Metformin as first-line T2DM drug.

Insulin's discovery in 1922 was a turning point in medicine, transforming DM from fatal disease into manageable chronic condition. Since then, therapy has expanded into diverse sets of pharmacologic classes, each targeting different aspects of glucose regulation. Insulin is the first lifesaving therapy for T1DM and advanced T2DM. However, insulin being the most effective, increases weight, and induces hypoglycemia.^7^ Biguanides primarily treat T2DM with Metformin 1 as first-line drug that reduces hepatic glucose production, improves insulin sensitivity, manages weight, and reduces heart disease (Fig. 1).^8^

**Fig. 2:**
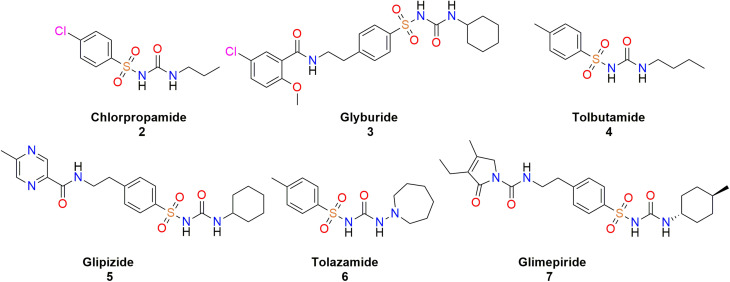
Structures of sulfonylureas as oral hypoglycemic agents in T2DM.

Sulfonylureas stimulate β-cells in pancreas, promoting insulin secretion in T2DM. Sulfonylureas (Chlorpropamide 2, Glyburide 3, Tolbutamide 4, Glipizide 5, Tolazamide 6, Glimepiride 7) cause significant hypoglycemia, weight gain, and gastrointestinal disturbances (Fig. 2).^9^

**Fig. 3:**
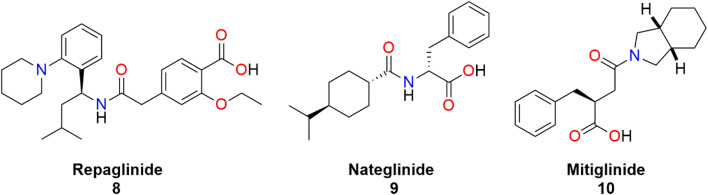
Structures of Meglitinides as class of short-acting oral antidiabetic drugs in T2DM.

Meglitinides are a class of short-acting oral antidiabetic drugs designed to manage post-meal blood sugar levels in T2DM by stimulating the pancreas to release insulin. Examples are Repaglinide (Prandin) 8, Nateglinide (Starlix) 9, and Mitiglinide (Glufast) 10. Side effects are hypoglycemia, and weight again (Fig. 3).^10^

**Fig. 4:**
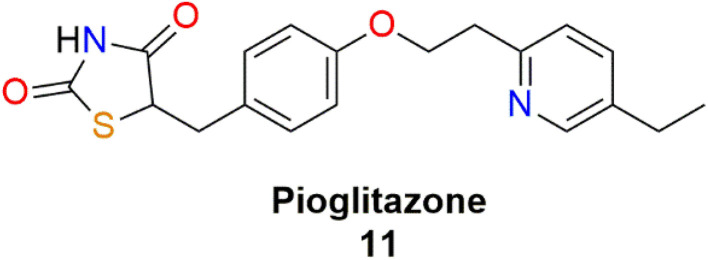
Pioglitazone (thiazolidinone) as oral antidiabetic drugs in T2DM.

Thiazolidinediones (TZDs) 11 are oral antidiabetic drugs, that increases insulin sensitivity, which helps lower blood glucose levels to manage T2DM. TZDs show side effects like weight gain, fluid retention, and higher risk of bone fractures (Fig. 4).^11^

**Fig. 5:**
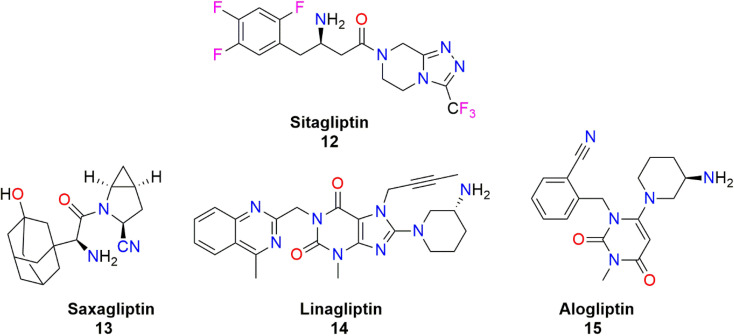
DPP-4 inhibitors as group of antihyperglycemic medications in T2DM.

Dipeptidyl peptidase 4 (DPP-4) inhibitors are a group of antihyperglycemic medications used to manage T2DM. FDA approved DPP-4 inhibitors are Sitagliptin 12, Saxagliptin 13, Linagliptin 14, and Alogliptin 15. Common side effects of DPP-4 inhibitors include respiratory infections, headaches, and nasopharyngitis (Fig. 5).^12^

**Fig. 6:**
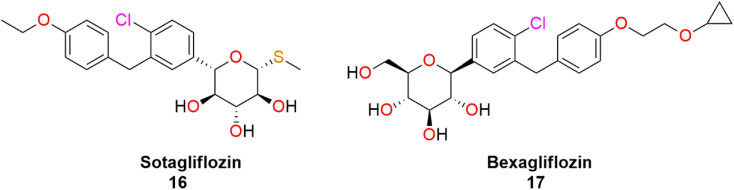
SGLT-2 inhibitors decrease blood glucose level in T2DM.

Sodium-glucose cotransporter-2 (SGLT2) inhibitors 16–17 decrease blood glucose level by causing the kidneys to excrete more glucose in urine, providing significant cardiovascular, and renal benefits. However, SGLT2 causes higher risk of genitourinary infections because the excess glucose in the urine can promote bacterial growth (Fig. 6).^12^

**Fig. 7:**
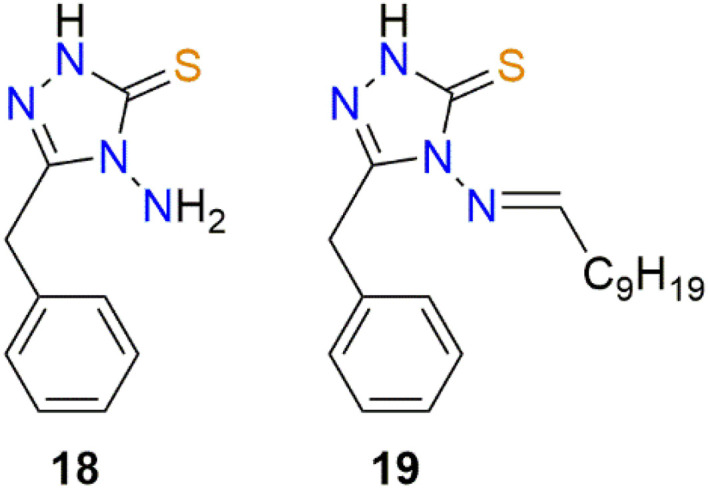
Triazolothione based a-glucosidase inhibitors.

α-Glucosidase inhibitors 18–19 delay digestion and absorption of carbohydrates, thus effectively blunt the sharp rise in blood glucose levels (postprandial hyperglycemia).^13^ Enzyme inhibition targeting dual pathways holds significant therapeutic potential against DM. This approach is associated with enhanced efficacy, and minimum side effects (Fig. 7).^14^

**Fig. 8:**
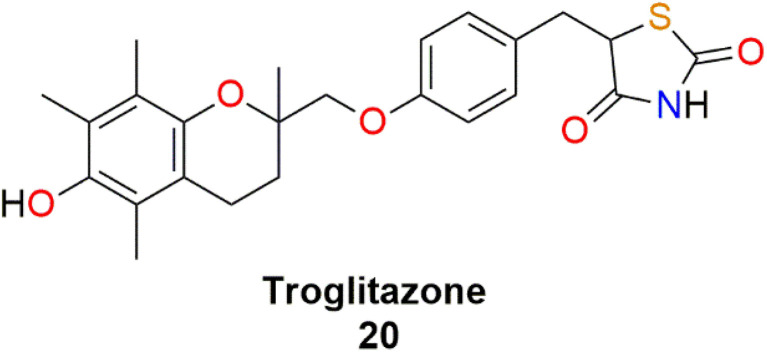
Agonists target GLP-1, and PPAR-γ signaling pathways in T2DM.

Agonists such as Liraglutide, and Semaglutide act through glucagon-like peptide-1 (GLP-1), while Troglitazone 20 target peroxisome proliferator-activated receptor gamma (PPAR-γ) signaling pathways, to manage T2DM. These medications increase insulin production, and sensitivity, unlike glucocorticoid receptor (GR) agonists which are not considered as standard treatment to combat T2DM (Fig. 8).^15–17^

**Fig. 9:**
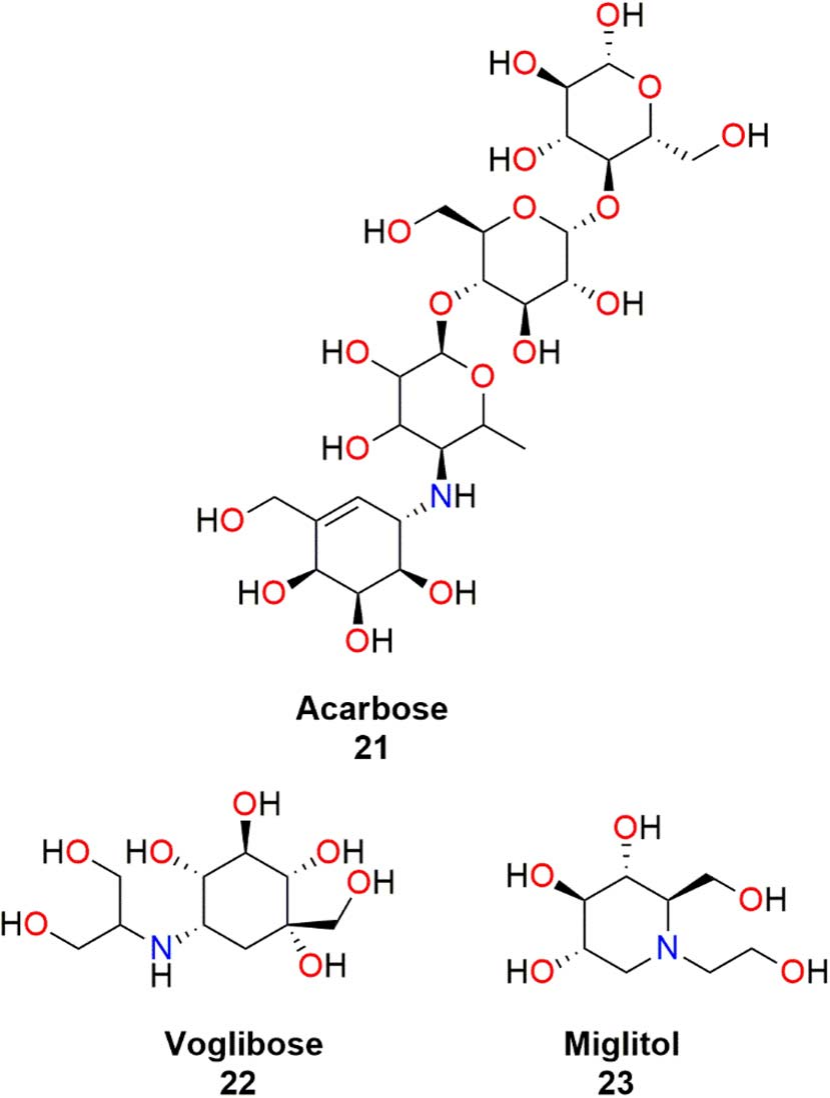
Some common, α-amylase, and α-glucosidase inhibitors.

GLP-1 is a gut hormone that regulates blood sugar by stimulating insulin release, and inhibiting glucagon secretion. GLP-1 slow gastric emptying, reducing appetite, promoting fullness, and is involved in physiological functions. GLP-1 agonists are class of medications that mimic these effects to treat T2DM. PPAR-γ is a nuclear receptor that regulates genes involved in glucose, and lipid metabolism. PPAR-γ is a key target for drugs used to treat insulin resistance. Dysregulation of PPAR-γ is associated with DM, and related complications. GR signaling contributes to DM by promoting hyperglycemia, and insulin resistance through mechanisms that include increasing glucose production, decreasing glucose uptake, and interfering with insulin signaling. However, GR signaling has a cell-specific role, sometimes causing GR resistance in immune cells, and potentially playing a role in β-cell glucose sensing, and other diabetic complications like retinopathy, and nephropathy. Therefore, GLP-1, PPAR-γ, and GR signaling pathways show distinct physiological functions, regulatory mechanisms, and therapeutic effects against metabolic diseases like T2DM.^15–17^ Pancreatic α-amylase enzyme digest complex dietary carbohydrates, by breaking specific internal bonds (α-1,4 glycosidic bonds) within these carbohydrates, turning them into smaller sugar molecules called oligosaccharides, further broken down into simple glucose by other enzymes, specifically intestinal α-glucosidases. This final glucose is absorbed into the bloodstream. Because this process directly leads to an increase in blood sugar levels after eating (postprandial sugar levels), it is a significant pathway for managing T2DM. Acarbose 21, Voglibose 22, and Miglitol 23 are common α-amylase, and α-glucosidase enzymes inhibitors (Fig. 9).^2,6^

Inhibition of carbohydrate-hydrolyzing enzymes, which delays glucose absorption, and moderates postprandial hyperglycemia represents an effective strategy for the management of T2DM. α-Amylases, secreted by salivary glands, and pancreas, initiate starch hydrolysis in oral cavity, and small intestine, producing disaccharides, and oligosaccharides. These intermediates are subsequently cleaved into glucose by α-glucosidases, located in the brush border of the small intestine, facilitating intestinal absorption. Pharmacological inhibition of these enzymes slows the rate of starch hydrolysis, thereby attenuating postprandial hyperglycemia. Clinically approved inhibitors such as Acarbose 21, Voglibose 22, and Miglitol 23 have demonstrated efficacy; however, their therapeutic utility is limited by gastrointestinal side effects including abdominal distention, flatulence, meteorism, and diarrhea. Moreover, long-term usage may lead to reduced responsiveness, underscoring the need for a broader pool of inhibitors to diversify treatment options. Given that the onset, and progression of T2DM are strongly influenced by dietary habits, lifelong adaptation, and strict adherence to nutritional guidance remain central to disease management. WHO recommends the incorporation of foods naturally rich in starch digestive enzyme inhibitors, both as dietary therapy, and as functional food ingredients, to complement pharmacological interventions, and enhance glycemic control.^18,19^

### 1.4. Dual drug targets

α-Amylase, and α-Glucosidase are enzymes involved in the digestion of dietary starch through the breakdown of starch, and sugar into simple glucose molecules.

#### 1.4.1. α-Amylase enzymes

α-Amylases (EC 3.2.1.1) termed as 1,4-α-d-glucanohydrolase, are hydrolytic enzymes belong to glycoside hydrolase group, produced by plants, animals, bacteria, fungi, and humans. α-Amylase catalyze hydrolysis of α-(1,4) glycosidic linkages of polysaccharides (Starches) into a mixture of oligosaccharides like maltose, maltotriose, α-(1–6), and α-(1–4) oligoglucans dependent upon the presence of a metal co-factor (Calcium). Human α-amylases are found in salivary glands that secrete enzyme into the mouth, and pancreas which secretes enzyme into small intestine. Starch, the most prevalent storage polysaccharide in plants, is a macromolecule composed of two types of polysaccharides, amylose (a linear α-(1→4) linked glucan), and amylopectin (α-(1→6) branched linkages). α-Amylases as endo-acting enzymes, hydrolyze interior parts of starch, specifically, α-(1→4)-glycosidic linkages to maltose, maltotriose, maltotetraose, maltodextrins, and glucose. Salivary α-amylase, perform partial digestion of polymeric starches, shorter oligomers then reach digestive system, for degradation into smaller oligosaccharides by pancreatic α-amylase. As oligosaccharides cross brush border membrane, α-glucosidase enzymes finally hydrolyze them into glucose.^18,19^

#### 1.4.2. Glucosidase enymes

α-Glucosidase (EC 3.2.1.20) systematically named as α-d-glucoside glycohydrolase are carbohydrate hydrolytic enzymes found in microorganisms, plants, animals, and human tissues. α-Glucosidases, are classified as maltase-glucoamylase (MGAM), and sucrase-isomaltase (SI). Each enzyme has two catalytic domains, N-terminal, and C-terminal subunit, termed ntMGAM, ctMGAM, ntSI, and ctSI. α-Glucosidase is found in luminal surface of enterocytes, secreted in small intestine, and catalyze hydrolytic cleavage of disaccharides (Maltose, Sucrose) into monosaccharides (Glucose, Fructose). MGAM belongs to glycosyl hydrolase family 31 (GH31). MGAM, and SI (EC 3.2.148, 3.2.10) hydrolyze low-weight oligosaccharides from α-amylases as final starch catabolism. α-Glucosidase catalyzes the hydrolysis of α-(1,4)-linked terminal non-reducing α-glucose residues to release α-glucose molecules, subsequently transported in bloodstream. Inhibition of α-glucosidase, halts blood sugar level, and suppress postprandial hyperglycemia. α-Amylase, maltase-glucoamylase, and sucrase-isomaltase, are starch-degrading enzymes, from glycoside hydrolase (GH) class. Salivary and pancreatic α-amylases are categorized within GH13 family, while MGAM, and SI under GH31 family.^18,19^

To conclude, starch digestion begins with salivary α-amylase in oral cavity, and pancreatic α-amylase in intestine, yielding maltotriose, glucose, maltose, and oligosaccharides. α-Amylases are unable to hydrolyze α-1,6 linkages in amylopectin or α-1,4 bonds adjacent to α-1,6 branches. The resulting oligosaccharides are subsequently converted into absorbable monosaccharides by the brush-border enzymes maltase-glucoamylase (MGAM) [α-glucosidase (EC 3.2.1.20) and glucoamylase (EC 3.2.1.3)], and SI [sucrase (EC 3.2.1.48) and isomaltase (EC 3.2.1.10)]. All four α-glucosidases catalyze the hydrolysis of α-1,4 glycosidic linkages, with ctMGAM (EC 3.2.1.20) showing the highest activity, liberating glucose from non-reducing ends of oligosaccharides, contributing significantly to carbohydrate metabolism, and glycoprotein processing. In contrast, ntSI (EC 3.2.1.10) specifically cleaves α-1,6 bonds at the non-reducing ends of isomalt oligosaccharides, while ctSI (EC 3.2.1.48) facilitates sucrose digestion. Consequently, modulation of α-amylase, and brush-border α-glucosidase enzymatic potential is critical for maintaining glucose homeostasis, and represents an important therapeutic target in T2DM.^18,19^

### 1.5. Heterocyclic compounds

#### 1.5.1. Azole heterocycles

Azoles represent one of the most prevalent classes of natural, and synthetic heterocycles, modulating diverse biological processes both in plants, and animals. Azoles structural versatility, and functional relevance have established them as indispensable tools in medicinal chemistry. Notably, U.S. Food, and Drug Administration reports that approximately 60% of approved low-molecular-weight therapeutics incorporate azole motifs, underscoring their inherent structural significance, and synthetic feasibility. This remarkable prevalence highlights their central role in modern drug discovery, where azoles function as privileged scaffolds in computational, and rational design strategies to optimize pharmacological activity, and drug-like properties.^20^

#### 1.5.2. Thiazole heterocycles

**Fig. 10:**
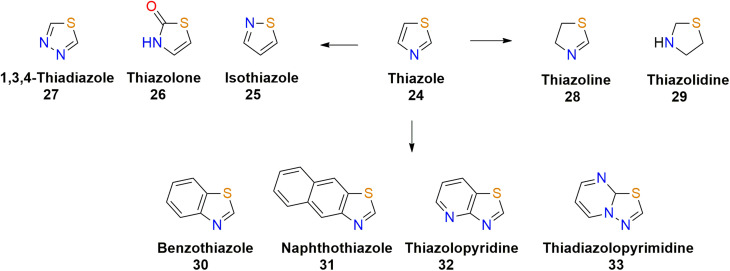
Structural analogues of 1,3-thiazole heterocycle.

Thiazole moiety was first described by Hantzsch and J. H. Weber in 1887 as “the pyridine of the thiophene series.” Subsequently, in 1889, Popp identified, and confirmed its structure.^21^ Thiazoles are five-membered aromatic heterocycles containing both nitrogen, and sulfur atoms, positioned at 1,3-locations of the ring 24.^22,23^ Structural analogues of thiazole include isothiazole 25, thiazolone 26, 1,3,4-thiadiazole 27, hydrogenated analogues such as thiazoline 28, and thiazolidine 29, as well as fused systems include benzothiazole 30, naphthothiazole 31, thiazolopyridine 32, and thiadiazolopyrimidine 33 (Fig. 10).^24^

**Fig. 11:**
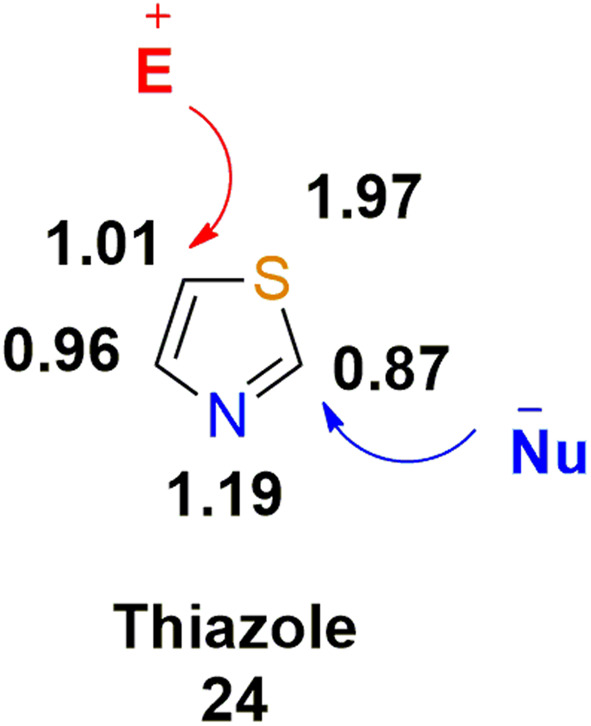
Distribution of π-electron density in thiazole ring.

Physical characterization of thiazole revealed a molecular formula of C_3_H_3_NS, specific gravity of 1.2, yellow coloration, and pyridine-like odor. Thermodynamic studies established the boiling point of thiazole at 118.24 °C. Thiazole is soluble in alcohol, and ether, slightly soluble in water, and miscible with dyes. Thiazole exhibits a dipole moment of 1.61 D with an orientation value of −53°, relative density of 1.1998, and refractive index of 1.5669. Chemical investigations confirmed the planar nature of thiazole, and augmented tiazole stronger aromaticity in comparision to other azoles.^21^ Resonance analysis demonstrated aromatic character of thiazole ring, due to the delocalization of lone pair of electrons on sulfur atom, which contributes to 6π-electron system. The distribution of π-electron density indicates that electrophilic substitution preferentially occurs at C-5, and C-4 positions, whereas nucleophilic substitution predominantly takes place at C-2 position (Fig. 11).^24^

#### 1.5.3. Occurrence of thiazole heterocycles

**Fig. 12:**
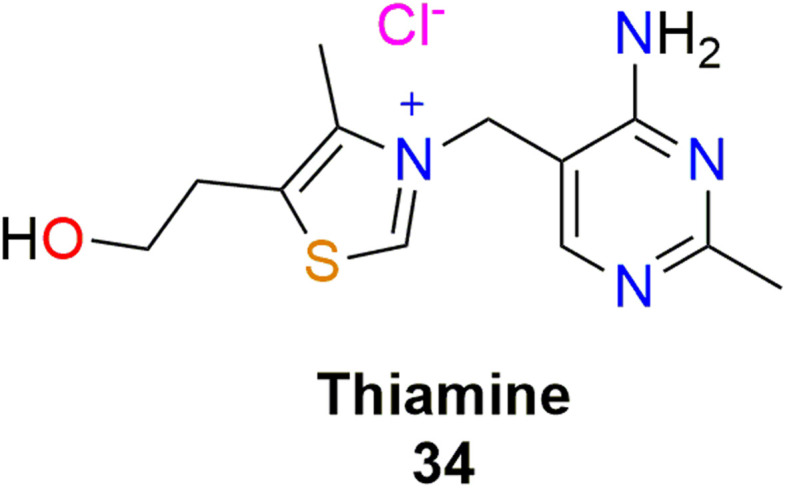
Thiamine essential component of CNS contains thiazole ring.

Thiazole heterocycles are widely distributed in natural products such as tomatoes, roasted coffee, thiamine, and vitamin B coenzymes, and serve as as distinct pharmacophores.^21^ Owing to their versatility as bioactive scaffolds, thiazoles are regarded as a privileged “wonder nucleus” in medicinal chemistry. Thiazole does not exist naturally, rather occurs in various natural products, including metabolites, cyclopeptides, alkaloids, anabolic steroids, flavones, and vitamin B_1_ (Thiamine) (Fig. 12).^25^ Vitamin B_1_34 occurs naturally in various foods and marine sources. Vitamin B_1_ is an essential water-soluble vitamin for mitochondrial energetics, particularly in ATP formation. Vitamin B_1_, acts as vital coenzyme in glucose, and amino acid metabolic pathways for living organisms.^26^

**Fig. 13:**
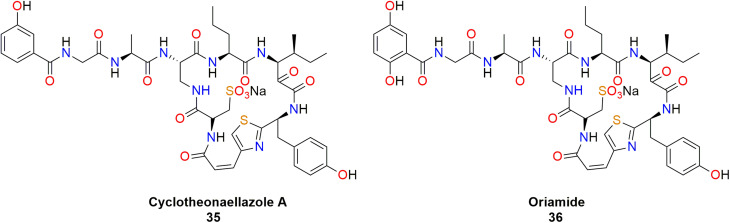
Thiazole based cyclotheonellazole A 35, and oriamide 36 isolated from marine sponge *Theonella* sp.

Cyclotheonellazole A 35, and Oriamide 36 are structurally related cyclic peptides isolated from marine sponge *Theonella* sp*.* Both contain a mix of proteinogenic amino acids and several unusual, non-proteinogenic amino acids, mostly novel 4-propenoyl-2-tyrosylthiazole unit (Fig. 13).^27,28^

**Fig. 14:**
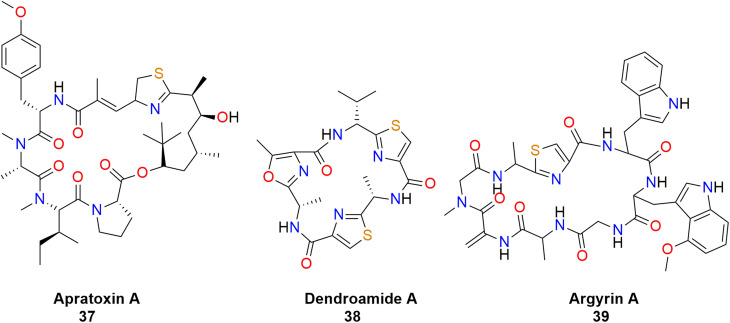
Natural products Apratoxin A 37, Dendroamide A 38, and Argyrin A 39 are thiazole-containing cyclic peptides.

Natural products Apratoxin A 37 is isolated from marine cyanobacterium *Lyngbya majuscula* as potent anticancer.^29^ Dendroamide A 38, isolated from cyanobacterium *Stigonema dendroideum Fremy,* exhibits antitumor activity that develops drug resistance.^30^ Argyrin A 39 is a thiazole containing cyclic peptide isolated from myxobacterium *Archangium gephyra,* associated with potent antitumor activity, and the ability to overcome drug resistance mechanisms (Fig. 14).^31^

**Fig. 15:**
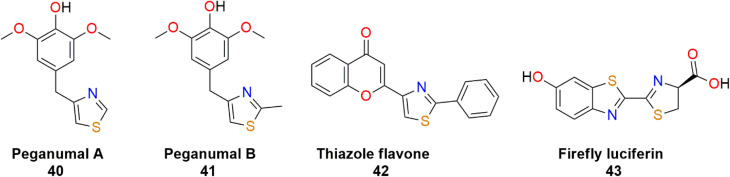
Thiazoles found in alkaloids 40–41, flavones 42, and firefly luciferin 43.

Thiazole occurs abundantly distributed in various natural products such as alkaloids (Peganumal A and B 40–41), flavones 42, and firefly luciferin 43 (Fig. 15).^25^

#### 1.5.4. Biological potential of thiazole heterocycles

Thiazole core is a privileged scaffold for constructing biologically and synthetically important compounds,^21^ as reflected by several marketed drugs. Elevated potency, and capability to engage diverse pharmacological targets turn thiazole-containing motifs valuable in drug discovery. Consequently, researchers continually modify existing inhibitor frameworks to generate more effective and selective derivatives.^21,32^ Thiazoles display versatile roles as anti-inflammatory, anticonvulsant, antituberculosis, antidepressant, anticancer, antibacterial, antifungal, antimalarial, monoamine oxidase, carbonic anhydrase, EGFR tyrosine kinase, and xanthine oxidase inhibitors.^21^

**Fig. 16:**
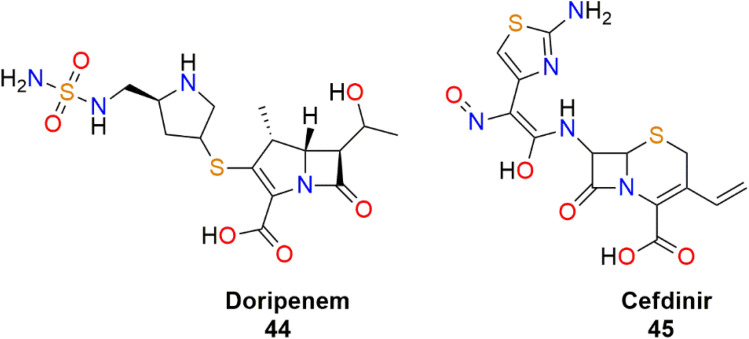
β-Lactams show synergy in pansusceptible, and resistant *Mycobacterium abscessus*.

Thiazoles serve as promising lead against antibiotic resistance. Thiazoles effect disruption of biofilms, cell-wall permeability, inhibit DNA gyrase, topoisomerase IV, and tryptophanyl-tRNA synthetase with MICs in micromolar range.^33^ Dual-β-lactam therapy (Doripenem 44, Cefdinir 45) shows reproducible *in vitro* synergy against both pansusceptible, and resistant *Mycobacterium abscessus* isolates, making it as promising therapeutic strategy (Fig. 16).^34^ The broader antimicrobial, and pharmacological potential of thiazoles extend from inhibition of bacterial, and protozoan infections to *Mycobacterium tuberculosis*, and anti-diuretic, as well as potential anti-Alzheimer effects (inhibition of amyloid plaques).^35^

**Fig. 17:**
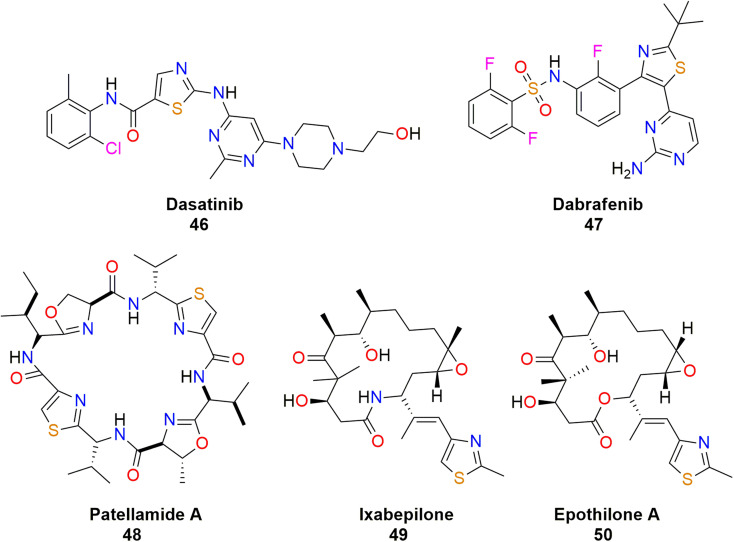
Thiazole heterocycles modulate diverse molecular pathways in therapeutic design.

Thiazole ring engages biomolecules *via* weak interactions like hydrogen bonds, π–π stacking, and van der Waals forces, serving as an excellent scaffold for small-molecule drug design.^36^ Thiazole structural adaptability assists the development of novel therapeutics with improved potency, and target engagement. Incorporating thiazole motifs into drug candidates often enhances efficacy, selectivity, bioavailability, and metabolic stability, thereby strengthening overall pharmacological profiles. Given their versatility, and favorable interactions with target, thiazole heterocycles are considered as indispensable tools for devising innovative strategies against complex, and multifactorial diseases. Thiazole scaffold functions as privileged ligand with broader pharmacological profile such as antihypertensive, hypnotic, neuroprotective, antipsychotic, diuretic, anti-oxidant,^20^ antitubercular, antiviral, antidiabetic, anthelmintic, and cardiovascular. Thiazole ring serves as modular, and pharmacologically active scaffold in many marketed, and investigation agents. Dasatinib 46, and Dabrafenib 47 illustrate how thiazole-based structures can selectively inhibit protein kinases involved in cancer signaling. Natural, and semi-synthetic macrocycles such as Patellamide A 48, and polyketide-derived agents like Ixabepilone 49, and Epothilones 50 suggest thiazole motifs contribution to compounds that stabilize or disrupt microtubules, producing potent anticancer effects. Together these examples emphasize thiazole core's adaptability for modulating diverse molecular pathways in therapeutic design (Fig. 17).^36,37^

**Fig. 18:**
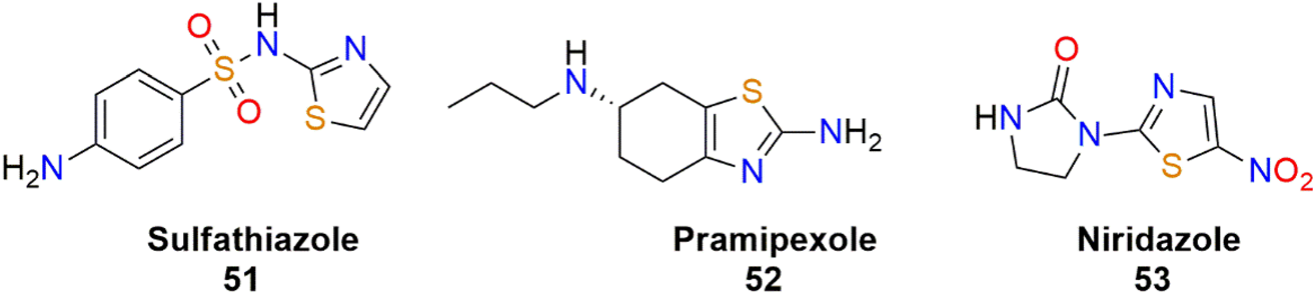
Thiazole based antimicrobial, antiparkinson, and anti-infections drugs.

Thiazole as excellent pharmacophore shows various biological applications as anti-inflammatory, and analgesic.^38^ Thiamine 34 is a thiazole-containing vitamin used to prevent, and treat deficiency supporting essential physiological functions, and overall health. Sulfathiazole drug 51 is used to treat microbial infections, inhibiting bacterial folic acid synthesis. Pramipexole 52 is a dopamine-agonist medication commonly used to treat motor symptoms of Parkinson's disease, and approved for restless legs syndrome. Niridazole 53 is a nitrothiazole with schistosomicidal activity, and treats infections caused by *Schistosoma species* (Fig. 18).

**Fig. 19:**
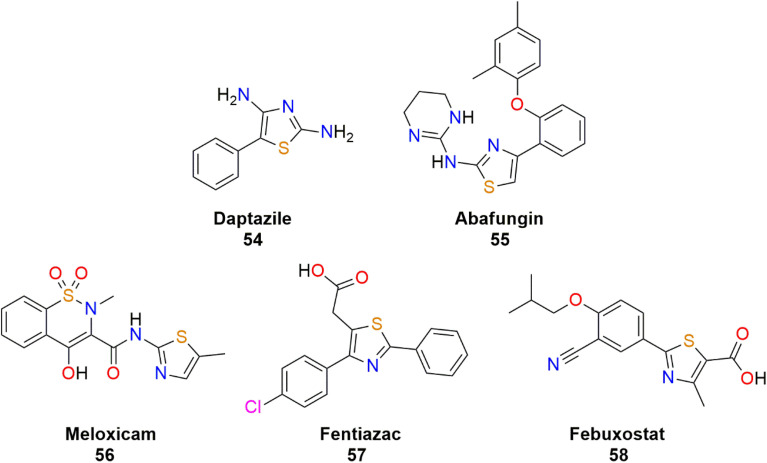
Thiazole drugs against respiratory depression, infections, arthritis, pain, and uric acid.

Amiphenaole (Daptazile) 54 is a non-opioid respiratory stimulant, used to counteract respiratory depression, and sedation caused by barbiturates, and opioids. Abafungin 55 is an investigational broad-spectrum topical antifungal developed for dermatomycoses, and other superficial fungal infections. Meloxicam 56 NSAID relieves inflammation, swelling, stiffness, and joint pain associated with osteoarthritis, and rheumatoid arthritis. Fentiazac 57 is a thiazole-based NSAID developed for relief of inflammatory joint, and muscular pain. Febuxostat 58 is a xanthine-oxidase inhibitor used to lower serum uric acid in adults with gout (Fig. 19)^32,38^ Thiazole moiety is an important framework of pharmaceutically important compounds due to the diverse biological activities, such as CNS active agents, and DNA targeting.^25^

**Fig. 20:**
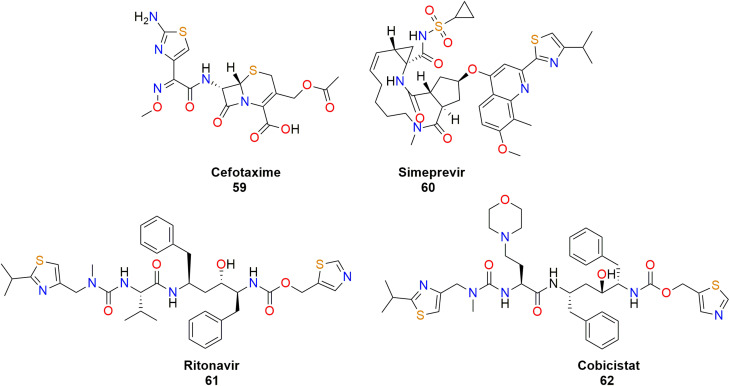
Thiazole based antimicrobial, and antiviral drugs.

Molecular hybridization is a powerful approach in advanced drug design, and development that involves the combination of two or more different pharmacophoric moieties to produce a new hybrid, and potent compound as drug candidate.^39,40^ Thiazole heterocycles have attracted considerable attention because of their favorable electronic properties, and structural versatility, which enable a wide range of chemical, and biological functions. More than 20 FDA-approved drugs incorporate a thiazole scaffold. Thiazole-containing drugs are used clinically; for example, the thiazole-bearing cephalosporin Cefotaxime 59 is an effective antibiotic for treating infections of the lower respiratory tract, and the genitourinary system. Simeprevir 60 is an antiviral agent indicated for the treatment of chronic hepatitis C virus (HCV) infection. Ritonavir 61, and Cobicistat 62 are primarily used as pharmacokinetic boosters for other antiretroviral drugs used to treat HIV/AIDS (Fig. 20).^41^

**Fig. 21:**
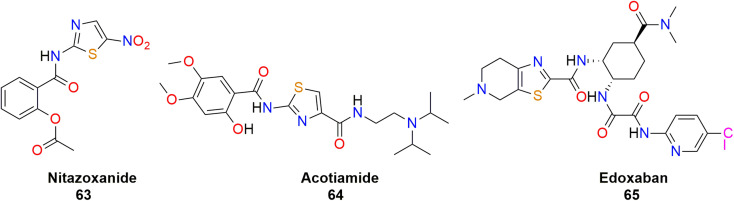
Thiazole based antiparasitic, antidyspepsia, and anticoagulant drugs.

Nitazoxanide 63 is an antiparasitic agent indicated for the treatment of diarrheal illnesses caused by *Cryptosporidium parvum,* and *Giardia lamblia.* Dasatinib 46 is an oncology drug indicated for the treatment of chronic myelogenous (myeloid) leukemia, particularly Philadelphia chromosome-positive CML. Acotiamide 64 is a gastrointestinal agent indicated for the treatment of functional dyspepsia. Edoxaban 65 classified as an anticoagulant, is used for the treatment of stroke, and systemic embolism (Fig. 21).^41^

**Fig. 22:**
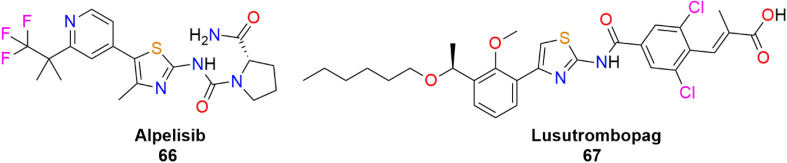
Thiazole based cancer inhibitor, and oral thrombopoietin receptor agonist.

NSAID Meloxicam 56 is used to relieve pain, and inflammation associated with musculoskeletal disorders. Alpelisib 66 is an oral PI3Kα-selective inhibitor indicated, in combination with fulvestrant, for the treatment of PIK3CA-mutated, hormone receptor-positive, HER2-negative advanced or metastatic breast cancer. Lusutrombopag 67 is an oral thrombopoietin receptor agonist used in oncology settings to raise platelet counts in patients with chronic liver disease-thereby reducing bleeding risk, and the need for platelet transfusion prior to invasive procedures. Owing to their broad spectrum of bioactivity, thiazole-based compounds are regarded as promising scaffolds for the development of therapeutics across a wide range of disease areas (Fig. 22).^41^

Moreover, thiazole motifs have exhibited significant miscellanous applications such as liquid crystals, sensors, dyes, pigments, and catalysts. Thiazole derivatives are extensively employed in materials sciences, pharmaceutical chemistry, and agrochemicals, underscoring their broad scientific, and industrial relevance.^41^

### 1.6. Antidiabetic potential of thiazole heterocycles

Thiazole heterocycles possess electron-rich, and polarizable ring systems which interact with enzymes, and receptors to regulate glucose homeostasis. Thus, thiazole heterocycles are extensively investigated as antidiabetic agents, yielding potent anti-oxidant, anti-inflammatory, DPP-4, Aldose reductase, α-glucosidase, and α-amylase inhibitors as well as PPAR-γ modulators.

#### 1.6.1. DPP-4 inhibition

**Fig. 23:**
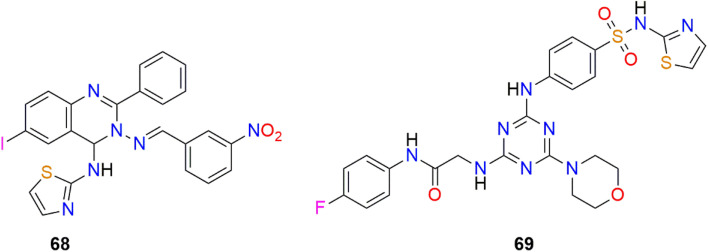
Thiazole based DPP-4 inhibitors, against T2DM.

DPP-4 enzyme breaks incretin hormones like GLP-1, increases insulin secretion, and lowers blood glucose. Thiazole-based DPP-4 inhibitors treat T2DM with comparable inhibitions to standard inhibitors like Linagliptin. 68 inhibited DPP-4 (IC_50_ = 0.76 nM), compared to Linagliptin (IC_50_ = 0.32 nM), and significantly reduced blood glucose level in Streptozotocin rat models. MD analysis of inhibitor 68 compared to Linagliptin, showed hydrogen bonding between oxygen of nitro group, and nitrogen of thiazole ring of 68 with hydroxyl group of Arg 125, and Arg 699. π-Interactions appeared with Arg 125, Phe 357, and Tyr 547 with phenyl, and thiazole ring of 68. Hydrophobic interactions between phenyl ring, and quinazoline ring occur with Glu205, Try 662, Try 631, Asn 710, Trp 659, Ser 630, Gly 632, Val 546, Hie 740, and Lys 544. MD suggests better binding (−6.27 kcal mol^−1^) of 68 to DPP-IV (PDB code: 2RGU) than DPP-IV inhibitor Linagliptin.^42^ Novel sulphonamide-triazine-thiazole hybrid 69 emerged as more effective DPP-4 inhibitor (2.32 nM) than Alogliptin (3.56 nM). 69 efficiently docked into the active site of the catalytic triad of Ser630, Asp708, and His740, encompassing both S1, and S2 pocket with a CDOCKER interaction energy of 57.80 kcal mol^−1^. Similarly, *in vivo* blood glucose lowering effects showed 69 induced dose-dependent enhancement of glucose tolerance in ICR orally. 69 (30 mg kg^−1^) reduced area under curve [(AUC) 0–120 min] to 37.46%, like hypoglycemic profile of Alogliptin. 69 demonstrated a dose-dependent reduction in blood glucose levels, and an improvement in insulin secretion, primarily through DPP-4 inhibition in STZ-induced diabetic rats (Fig. 23).^43^

#### 1.6.2. PPAR-γ modulation

**Fig. 24:**
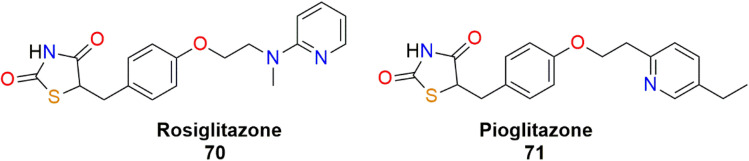
Thiazole based PPAR-γ inhibitors, against T2DM.

Selective PPAR-γ modulation treats T2DM, because this nuclear receptor is a master regulator of glucose homeostasis, and insulin sensitivity. PPAR-γ activation improves insulin sensitivity, glucose metabolism, and modulates inflammatory responses. Thiazolidinedione based anti-diabetic Rosiglitazone 70, and Pioglitazone 71 as PPAR-γ full agonists, have been validated clinically for improved insulin sensitivity, and control glucose levels in T2DM patients (Fig. 24).^44,45^

#### 1.6.3. Anti-oxidant, and anti-inflammatory pathways

**Fig. 25:**
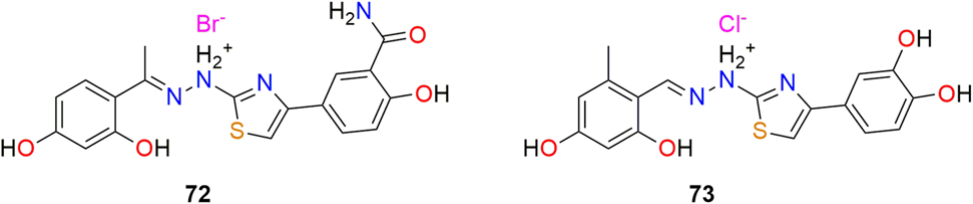
Thiazole based anti-oxidant and anti-inflammatory inhibitors.

Thiazoles quench reactive species like superoxide, and hydroxyl radicals, reducing oxidative stress. Some thiazoles bind transition metals, stopping Fenton reactions that generate RONS.^46,47^ Generally, oxidative stress, and inflammation contribute to insulin resistance. Thiazole moieties often increase radical-scavenging potential, and can inhibit pro-inflammatory mediators, improving insulin sensitivity indirectly. Thiazoles exhibit notable anti-oxidant, and anti-inflammatory properties that contribute to their antidiabetic potential. Thiazoles effectively quench RONS, and chelate transition metals thereby reducing oxidative stresses. Since oxidative stress, and chronic inflammation are key contributors to insulin resistance, thiazole moieties with radical-scavenging potential, and inhibitory effects on pro-inflammatory mediators can indirectly improve insulin sensitivity. Collectively, these properties highlight thiazoles as versatile pharmacophores for the design of multifunctional agents targeting both oxidative, and metabolic pathways in T2DM 72–73 (Fig. 25).^48,49^

#### 1.6.4. Aldose reductase inhibition

**Fig. 26:**
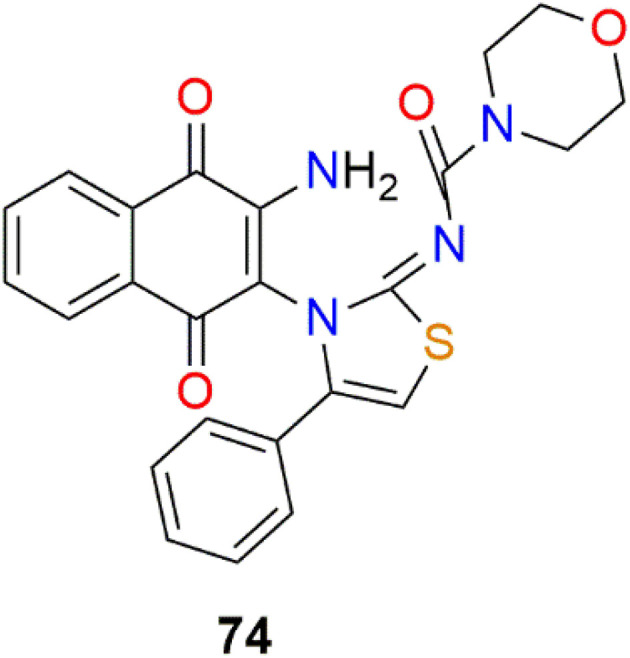
Thiazole based aldose reductase inhibitors.

Thiazoles inhibit aldose reductase, a key enzyme in polyol pathway that catalyzes the reduction of glucose to sorbitol. Excessive activation of this pathway under hyperglycemic conditions leads to sorbitol accumulation, osmotic stress, and secondary complications such as diabetic neuropathy, retinopathy, and nephropathy. By inhibiting aldose reductase, thiazole analogues can reduce sorbitol-induced cellular damage, and thereby mitigate the progression of these chronic diabetic complications. This mechanism highlights the potential of thiazole scaffolds not only in glycemic control but also in the prevention of long-term microvascular complications, positioning them as multifunctional agents in T2DM therapy 74(Fig. 26).^50^

#### 1.6.5. α-Glucosidase, and α-amylase inhibition

**Fig. 27:**
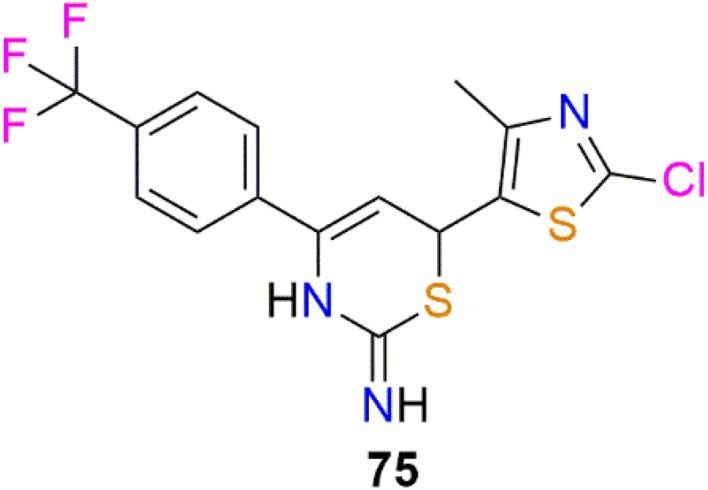
Thiazole hybrids as α-glucosidase, and α-amylase inhibitors.

Thiazole hybrids have emerged as promising scaffolds in developing antidiabetic agents.^51,52^ These hybrids inhibit α-amylase, and α-glucosidase enzymes, which catalyze the breakdown of complex carbohydrates into glucose. Inhibition of these enzymes slows carbohydrate digestion, and reduces intestinal glucose absorption, thereby attenuating sharp rises in postprandial blood glucose levels. This mechanism provides a gradual release of glucose into the bloodstream, helping to maintain glycemic control. Such enzyme inhibition is a well-established therapeutic strategy in T2DM, exemplified by clinically used drugs such as Acarbose. However, thiazole hybrids offer novel structural diversity and the potential for enhanced efficacy, and reduced adverse effects, positioning them as attractive candidates for further optimization in antidiabetic drug discovery (Fig. 27).^51,52^

##### 1.6.5.1 Scope of the review

The present review article provides a comprehensive survey of recent literature (2020–2025) on thiazole heterocycles, synthesizing information on their structural analysis, natural occurrence, commercial significance, and pharmacological relevance. Emphasis is given to the anti-oxidant potential, antidiabetic effects, and molecular docking analysis of the synthesized thiazoles. Several FDA-approved thiazole-based drugs, and their clinical analogues are highlighted, reflecting their therapeutic significance. Optimized synthetic strategies for the synthesis of both simple, and hybrid thiazole heterocycles are discussed, alongside *in vitro* biological evaluations against key antidiabetic targets like α-amylase, α-glucosidase, Aldose reductase, DPP-4, and PPAR-γ agonists, with comparison to standards. Oxidative stress is a major contributor to the onset, and progression of DM. The review highlights anti-oxidant potential of thiazoles, particularly their ability to inhibit DPPH, and ABTS radicals. These findings emphasize dual role of thiazoles, as antidiabetic, and anti-oxidant, augmenting the therapeutic potential of thiazoles in mitigating oxidative damage while improving metabolic regulation. SARs analysis correlates structural variations with obtained biological potencies, while docking simulations provide deeper insights into ligand–receptor interactions. Collectively, these findings offer valuable insights into rational design, and development of potent thiazole scaffolds with enhanced anti-oxidant, and antidiabetic efficacy.

An updated literature survey revealed that I. Mishra *et al.* provides comprehensive overview of the progress achieved (2016–2025) in the development of potent thiazole-based anti-oxidants, including hydrainyl-thiazole hybrids, fused thiazoles, thiaole-azmethine based co-ordination compounds, and thiaole functionalized with diverse heterocycles. The review highlights therapeutic significance of thiazole derivatives in mitigating oxidative stress, and managing associated pathological conditions, including cancer, neurodegenerative disorders, cardiovascular diseases, inflammation, and diabetes. A major focus of the review is the impact of structural modifications within thiazole scaffold, demonstrating that even subtle variations in substituents can profoundly influence anti-oxidant potency, molecular stability, and overall biological efficacy. Notably, thiazole derivatives bearing electron-donating substituents, catechol moieties, azomethine linkages, and thiaole functionalized with heterocyclic hybrid frameworks exhibited enhanced free-radical scavenging activity, frequently surpassing conventional anti-oxidant drugs. Furthermore, metal-co-ordinated thiazole compounds have been shown to enhance electron-transfer processes, and stabilize radical intermediates, thereby improving overall anti-oxidant efficiency. Mechanistic investigations revealed that these compounds predominantly exert their anti-oxidant effects through hydrogen atom transfer, and single-electron transfer pathways, driven by the synergistic electronic, and structural features of the thiazole nucleus. Collectively, the review underscores the pivotal role of SAR analysis in guiding rational design, and optimization of next-generation thiazole-based anti-oxidants with enhanced therapeutic potential, and pharmacological efficacy.^53^

In summary, the present review distinguishes itself by providing a more integrated perspective on thiazole chemistry, encompassing anti-oxidant, and antidiabetic pharmacology alongside synthetic strategies, molecular docking analysis, and translational drug-development approaches.

## 2 Antidiabetic potential of thiazole heterocycles

Molecular hybridization, achieved through covalent integration of two or more biologically relevant heterocyclic scaffolds with complementary mechanisms, is considered as a powerful strategy for designing multifunctional therapeutics with enhanced efficacy, and reduced toxicity. In the context of DM, heterocyclic hybrids exploit structural diversity, and tunable physicochemical properties to address the multifactorial nature of the disease. Among these, thiazole-based hybrids have attracted considerable interest owing to the metabolic stability, and broad biological relevance of thiazole nucleus. Recent synthetic efforts have yielded a wide array of thiazole-linked architectures-ranging from simple hybrids such as hydrazinyl-, azomethine-, pyrazole-, sulfonamide-, amide-, hydrazide-, imidazole-, and indole-based thiazoles to more complex systems including thiazolidinones, cholic acid-thiadiazoles, oxadiazole-thiadiazoles, coumarin-thiadiazoles, and spiro-pyrrolo-thiazole hybrids. Notably, advances in thiazoles synthesis using thiosemicarbazones, and phenacyl bromides as versatile precursors have enabled efficient access to structurally diverse thiazole hybrids with enhanced biological profiles. These molecular constructs exhibit promising antidiabetic activity through multi-target modulation, including DPP-4 inhibition, PPAR-γ modulation, improved insulin sensitivity, and suppression of carbohydrate-digesting enzymes (α-glucosidase, α-amylase) as well as aldose reductase inhibition, thereby controlling postprandial hyperglycemia, and mitigating long-term diabetic complications. Importantly, incorporation of anti-oxidant pharmacophores imparts RONS scavenging capacity, counteracting oxidative stress-mediated β-cell dysfunction. Collectively, thiazole-based molecular hybrids exemplify a rational, and synergistic design paradigm for next-generation antidiabetic agents that integrate metabolic regulation with anti-oxidant protection, offering the potential for improved therapeutic outcomes over conventional monofunctional drugs.

### 2.1. Hydrazinyl thiazoles

Hydrazinyl thiazoles have emerged as a promising class of heterocyclic hybrids exhibiting dual antidiabetic, and anti-oxidant properties. The strategic incorporation of a hydrazinyl moiety into thiazole framework enhances molecular flexibility, hydrogen-bonding capability, and redox responsiveness, thereby facilitating effective interactions with key biological targets involved in glucose homeostasis, and oxidative stress regulation. These hybrids act as efficient modulators of carbohydrate-hydrolyzing enzymes, and demonstrate potent inhibition of α-glucosidase, and α-amylase, often comparable to standards, thereby contribute to control postprandial hyperglycemia. In addition to glycemic regulation, several hydrazinyl-thiazole derivatives exhibit pronounced antiglycation activity, surpassing aminoguanidine in preventing advanced glycation end-product formation, and associated long-term diabetic complications. Furthermore, selected hydrazinyl-clubbed thiazoles have been identified as effective aldose reductase inhibitors, underscoring their role in mitigating diabetes-induced secondary complications. The electron-donating nature of the hydrazine functionality further imparts significant anti-oxidant activity, particularly through scavenging of DPPH, and ABTS radicals. Given the central role of oxidative stress in the progression of insulin resistance, and diabetic complications, the combined glucose-lowering, and anti-oxidant efficacy of hydrazinyl-substituted thiazoles positions them as attractive multifunctional candidates for next-generation antidiabetic therapy.

#### 2.1.1. Hantzsch synthesis of 1,3-thiazoles

**Scheme 1:**
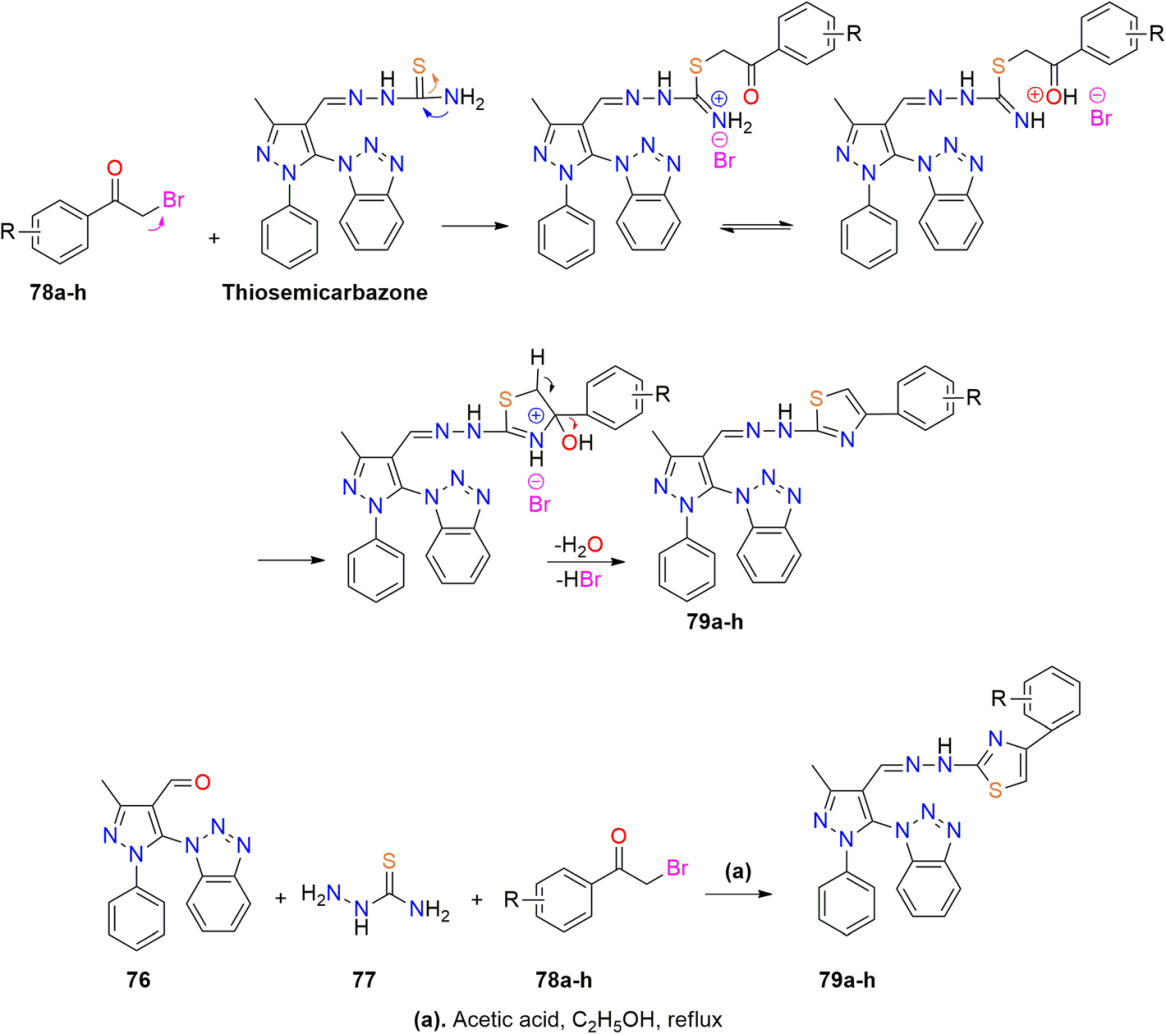
(A) Hantzsch's synthesis of thiazole using α-haloketone and thioamides. (B) Synthesis of triazole-pyrazole clubbed thiazole hybrids 79a–h.

Though literature survey presents various synthetic protocols for the construction of thiazole ring, however, the most common, simple, and efficient synthetic strategy includes Hantzsch synthesis of 1,3-thiazoles. In 1887, Arthur Hantzsch first reported an efficient method for the synthesis of thiazoles through the reaction of α-haloketones or α-haloaldehydes with thioamides, a classical approach that remains one of the most widely employed methods for thiazole ring construction (Scheme 1A).^54–56^

#### 2.1.2. Mechanism of Hantzsch's synthesis

Hantzsch's synthesis is the most common strategy used for the synthesis of 1,3-thiazoles that operates through cyclization, and condensation of haloketones with thioamides. The reaction starts by nucleophilic attack of sulphur atom of thioamide on the α-carbon atom of α-haloketone which results in the formation of α-thioketone intermediate. Following this, dehydration occurs which generates the respective thiazole ring.^54,55^

Keshav B. Gangurde *et al.* synthesized eight triazole-pyrazole based thiazoles 79a–h(Scheme 1B). Thiosemicarbazide 76, was poured into ethanolic solution of carbaldehyde 77, using acetic acid as catalyst, at reflux for 30 min. Later, phenacyl bromides 78a–h were added, and the reaction upon completion, was cooled, filtered, washed, and dried to get 79a–h.^56^

**Fig. 28:**
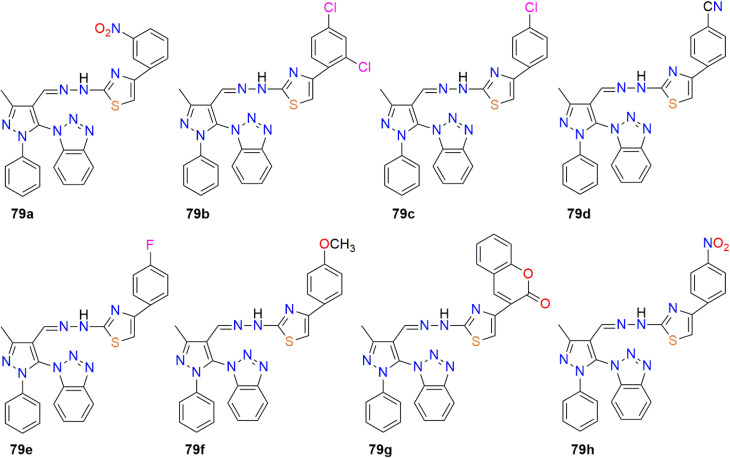
Structures of the synthesized triazole-pyrazole clubbed thiazole hybrids 79a–h.

79a–h were subjected to *in vitro* hydroxyl (OH), and DPPH% free radical scavenging assay (FRSA). Ascorbic acid (83.43 ± 0.81, 84.05 ± 0.55%), and α-Tocopherol (81.27 ± 0.99, 81.33 ± 0.31%) served as drugs. 79a–h served as good anti-oxidants, with 79a showing the most potent OH (78.06 ± 0.63%), and 79h as highest (75.63 ± 0.78%) DPPH FRSA. In OH FRSA, 79g (71.03 ± 0.45%), 79d (69.93 ± 0.66%), 79h (67.71 ± 0.62%), and 79c (67.65 ± 0.54%) showed good anti-oxidant potential. In DPPH FRSA, 79a (72.74 ± 0.0.70%), 79b (70.44 ± 0.67%), 79e (73.93 ± 0.26%), and 79g (72.52 ± 0.46%) showed similar, and good anti-oxidant effects. Thus, the screened hybrids served as good anti-oxidants compared to drugs (Fig. 28).^56^

**Scheme 2:**
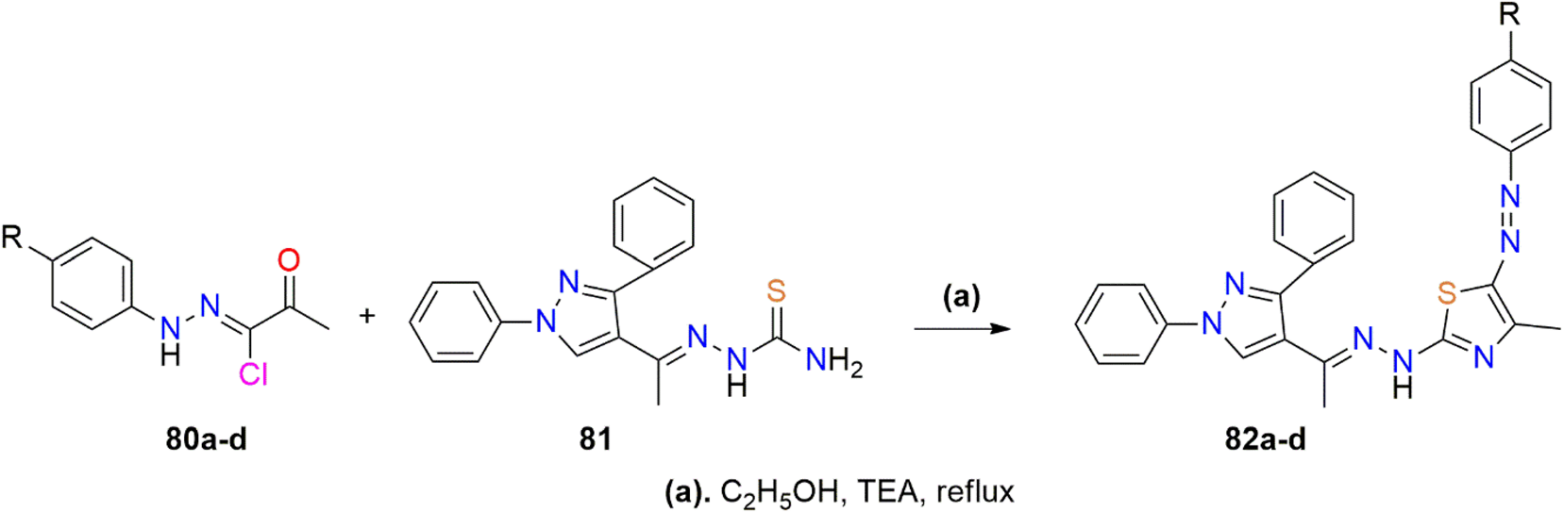
Synthesis of pyrazole-based hydrazinyl thiazoles 82a–d.

A. M. Hussein *et al.* synthesized four pyrazole-based hydrazinyl thiazoles 82a–d(Scheme 2). Thus, hydrazonoyl chlorides 80a–d, and thiosemicarbazone compound 81 mixed in ethanol, and TEA as catalyst, were refluxed for 1 h. The mixture was filtered, washed, and recrystallized from ethanol, and DMF to get products 82a–d.^57^

**Fig. 29:**
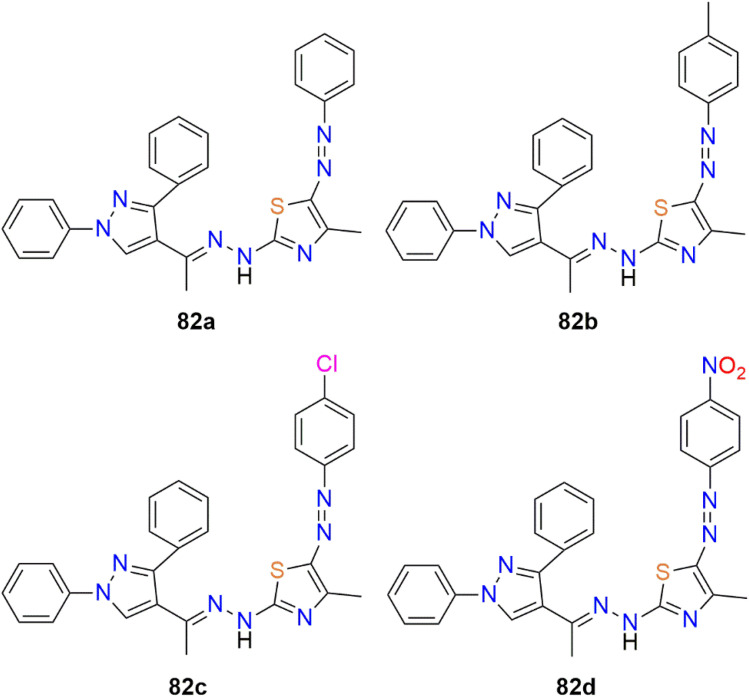
Structures of the synthesized pyrazole-based hydrazinyl thiazoles 82a–d.

82a–d were subjected to *in vitro* DPPH, and ABTS (2,2′-azino-bis-3-ethylbenzthiazoline-6-sulfonic acid) FRSA, to check their oxidative potential using Gallic acid as drug. 82a–d exhibited similar, and moderate % DPPH FRSA (38.03, 40.84, 39.44, 38.02%), very low than Gallic acid (83.10%) at five different concentrations (12.5, 25, 50, 100, 200 µg mL^−1^). Dose-dependent % FRSA was observed for Gallic acid, and all the screened hybrids, except 82b at 12.5, and 25 µg mL^−1^ concentration exhibited 40.84, and 38.03% DPPH FRSA. In contrast, 82a–d showed strong % ABTS FRSA in concentration dependent manner, and found to be comparable (93.13, 94.51, 93.81, 93.13%) to Gallic acid (96.29%) especially at higher concentration (200 µg mL^−1^) (Fig. 29).^57^

**Scheme 3:**
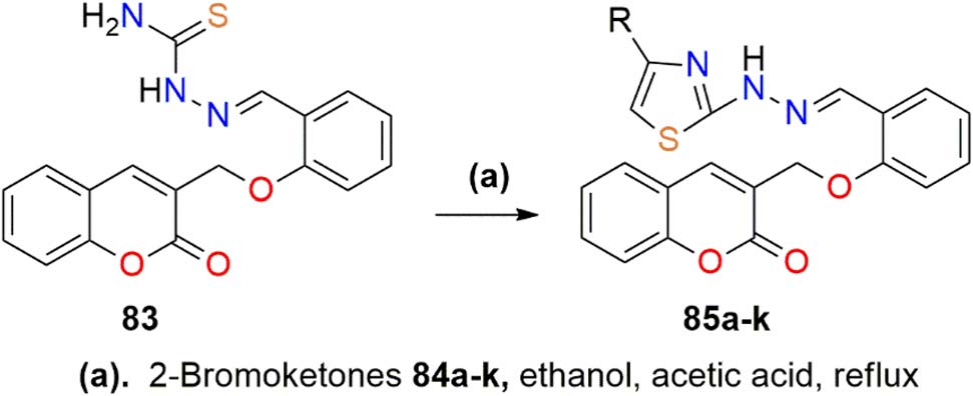
Synthesis of coumarin-based hydrazinyl thiazoles 85a–k.

Q. ul Ain *et al.* synthesized eleven coumarin-based hydrazinyl thiazoles 85a–k (Scheme 3). Thus, thiosemicarbazone compound 83, and 2-bromoketones 84a–k were refluxed in ethanol, using acetic acid as catalyst, for 6–8 h. The solids were filtered, washed, and recrystallized from ethanol to get target thiazole hybrids 85a–k.^58^

85a–k exhibited significant α-glucosidase enzyme inhibitions (IC_50_ = 1.88 ± 0.03–322.66 ± 1.14 µM) in comparison to Acarbose as drug (IC_50_ = 873.34 ± 1.67 µM). SAR suggest, 85a showed 870-folds higher inhibition (IC_50_ = 2.13 ± 0.04 µM) than drug. 85b–c displayed comparable α-glucosidase inhibition (IC_50_ = 3.15 ± 0.05, 2.75 ± 0.06 µM) to 85a. 85d showed lower inhibition (IC_50_ = 24.51 ± 0.16 µM), than 85a, and 85c. 85(e–f) showed similar potent α-glucosidase inhibition (IC_50_ = 4.68 ± 0.07, 4.32 ± 0.07). Inhibition decreased highly (IC_50_ = 322.61 ± 1.14 µM) in 85g, due to steric hindrance. 85h showed marked increase in α-glucosidase inhibition (IC_50_ = 2.59 ± 0.01 µM). 85i–j showed 800-folds more potential (IC_50_ = 4.42 ± 0.11, 3.68 ± 0.10 µM). In 85k, notable inhibition (IC_50_ = 1.88 ± 0.03 µM) was observed, and was ranked as the most potent antidiabetic agent. SARs suggests the presence of substituents across benzene ring strongly effect *in vitro* α-glucosidase inhibition, with most of the inhibitors associated with close, and highly potent antidiabetic effects. 85k, being the most potent, was evaluated kinetically to analyze the underlying mode of action, and revealed competitive mode of inhibition (*K*_i_ = 3.38 ± 0.181 µM).^58^

Molecular docking is an important *in silico* technique, used to investigate interactions between small molecules (ligands), and high-resolution 3D protein (receptors) structures. Molecular Docking (MD) analysis predicts the most favorable binding orientations, and interaction patterns of a ligand within the active site of a protein, providing insights into the molecular, and biochemical basis of ligand–receptor interactions. Strong intermolecular forces between the ligand, and protein indicate stable complex formation, associated with favorable binding affinity, and more neagtive binding energy. MD analysis enables efficient screening, comparision, and optimization of inhibitors to help identify promising drug candidates in drug design, and discovery programme.^59,60^

**Fig. 30:**
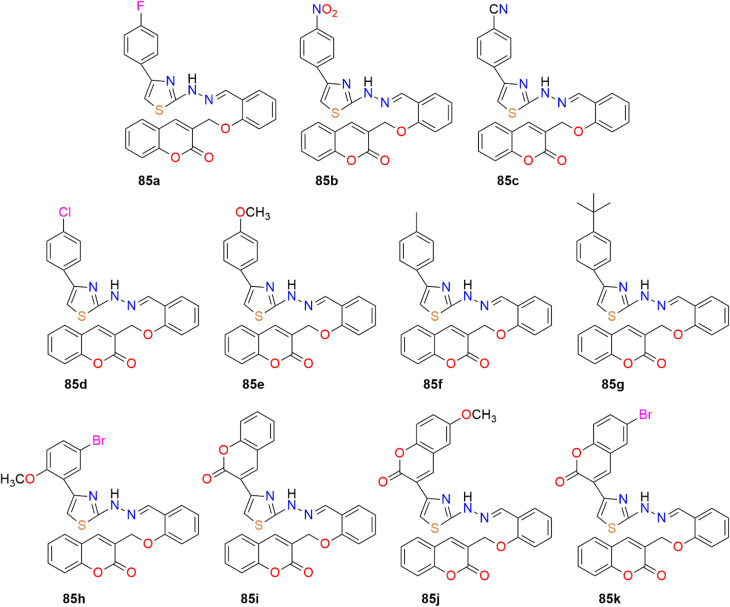
Structures of the synthesized coumarin-based hydrazinyl thiazoles 85a–k.

In the current research, SAR was analyzed through atom-based protein-ligand interaction. MD analysis provided insights into interaction of the molecules within the active sites of α-glucosidase, demonstrating their elevated antidiabetic capacity. 85k being the most potent, azomethine group donated hydrogen bond to the side chain of Glu411 (2.0 Å), and bromo-coumarin formed hydrogen bond through the backbone of amino group of Arg315 (2.31 Å). Moreover, benzene ring interacted with the side chain of Phe303 *via* hydrophobic (π–π) stablizations. 85k received higher score (−9.10 kcal mol^−1^), reflecting strong interactions with the target residues of the active site. Binding modes of related potent inhibitors 85a–c (−8.69, −8.54, −8.62 kcal mol^−1^), and 85h (−8.68 kcal mol^−1^), bear resemblance to 85k. Their azomethine group donated hydrogen bond to the side chain of Glu411. In 85a, and 85h, hydrophilic interactions decreased with the active site residues due to the lack of coumarin ring in their structures. Flouro ring of 85a, and bromo-methoxy ring of 85h lack polar interactions to the surrounding residues, instead thiazole, and coumarin rings in 85a formed hydrophobic with the backbone amino group of Arg315, and π-cation interactions with side chain of Arg442. Coumarin ring in 85h formed hydrophobic interaction with side chain of Tyr158, and Phe178. Due to reduced polar interactions with the active site residues, hydrophobic interactions stabilized 85a, and 85h within the active site. In 85c, thiazole ring showed hydrophobic interactions (Arg315), absent in coumarin and linker phenyl moiety of 85c with the surrounding residues. Like 85k, the linker phenyl rings of 85a, 85h, and 85b formed π–π interactions with the side chain of Phe303. Phenyl rings of 85c, and 85b formed hydrogen bond with side chain of Lys156 through cyano, and nitro groups. 85j (−8.46 kcal mol^−1^) exhibited significant inhibition (IC_50_ = 3.68 µM). 85j displayed conformational similarity to 85b, with both coumarin, and linker phenyl rings adopting identical orientations to 85b. In 85j, coumarin as substituent was unable to form any interactions because the azomethine group twisted toward Asp307, and created hydrogen bond with the side chain of Asp307 at 2.27 Å. 85j followed binding pattern, consistent with the related inhibitors (85a–b, 85h, 85k): aryl of the linker interacted Phe303 hydrophobically, and thiazole moiety formed hydrophobic contacts (Arg315), mirroring the behavior of 85a, and 85c. 85j lacks hydrophilic binding, and hydrophobic interaction of coumarin, decreasing inhibition compared to 85k, 85a, 85h, 85c, and 85b. 85f (−8.31 kcal mol^−1^), 85i (−8.10 kcal mol^−1^), and 85e (−8.11 kcal mol^−1^) showed inhibitions of 4.32, 4.42, and 4.68 µM. 85f showed hydrogen bonding (Glu411) *via* azomethine group like 85a, 85h, 85c, and 85b, and hydrophobic interaction of the linker phenyl ring (Phe303) like 85k, 85a, 85h, and 85b. Thiazole, coumarin ring, and tolyl group of 85f lack interactions, resulting in reduced inhibition. Positioned near the active site opening, 85i engaged merely in hydrogen bonding between azomethine group, and side chain of His280. 85e mirrored binding pattern of 85j, and displayed comparable interaction behavior. 85e resembled 85j, characterized by hydrogen bonding of the azomethine group (Asp307), and hydrophobic interaction of the linker phenyl ring (Phe303). 85d (−6.14 kcal mol^−1^), with moderate potency, mirrored binding pose of 85h. The presence of *para*-chloroaryl group shifted inhibitor's orientation towards the active site opening, thereby thiazole, linker aryl, and coumarin moieties being displaced into the entrance loop. 85d lacked hydrophobic stabilization, and relied solely on hydrogen bonding of its azomethine group with Tyr158. 85d has significantly reduced inhibition compared to 85f, 85i, and 85e. Lowest inhibition of 85g (IC_50_ > 322 µM) reflects the detrimental influence of *t*-butyl aryl group at *para*-position. Because of this group, 85g (−5.89 kcal mol^−1^) assumed conformation akin to 85d near the active site entrance. Consequently, hydrophobic interaction with Phe303 was mediated exclusively by the linker aryl ring, while the azomethine moiety failed to interact. Due to the added group, 85g adopted 85d-like conformation near the active-site entrance, allowing only the linker aryl ring to interact hydrophobically with Phe303, while the azomethine group did not participate in binding. Without stabilizing hydrogen bonds or hydrophobic contacts, the hybrid emerged as least potent analogue (Fig. 30).^58^

**Scheme 4:**
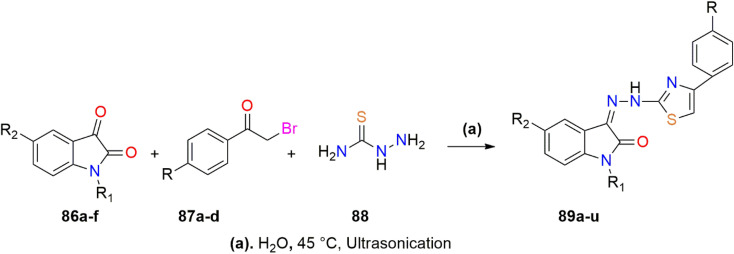
Synthesis of 3-[2'–(4′-arylthiazol-2′-yl)hydrazono]indolin-2-ones 89a–u.

**Scheme 5:**
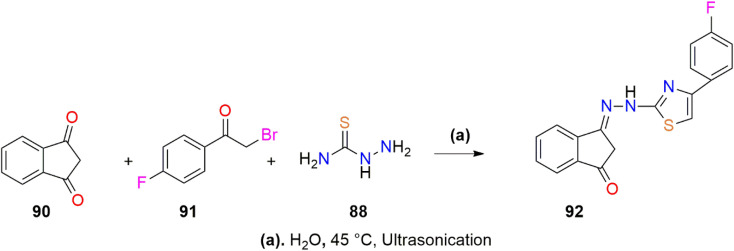
Synthesis of 3-{2-[4’–(4″-fluorophenyl)thiazol-2-yl]hydrazono}-2,2-dihydroinden-1-one 92.

**Scheme 6:**
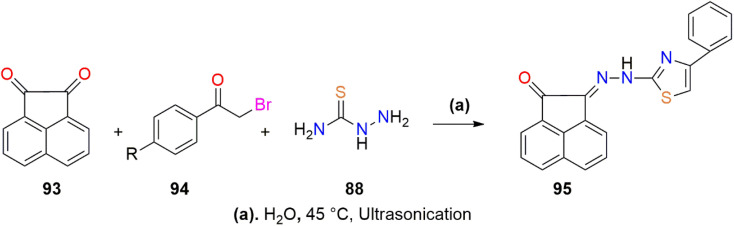
Synthesis of 2-[2-(4′-phenylthiazol-2-yl)hydrazono]acenaphthylen-1-one 95.

**Scheme 7:**
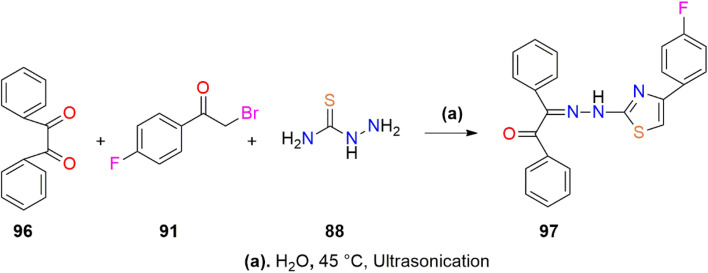
Synthesis of 2-{2-[4′–(4″-fluorophenyl)thiazol-2-yl]hydrazono}-1,2-diphenylethanone 97.

A. Khanum, and M.A. Pasha *et al.* synthesized twenty-four hydrazino-thiazoles 89a–u, 92, 95, and 97. Isatins/1,3-indane-dione/acenaphthenequinone/benzil, hydrazine carbothioamide, 2-bromo-1-phenylethanones, and distilled water were sonicated at 45 °C for 30–35 min. The mixture was stirred, washed, and recrystallized from ethanol to get 89a–u, 92, 95, and 97(Schemes 4–7).^61^

**Fig. 31:**
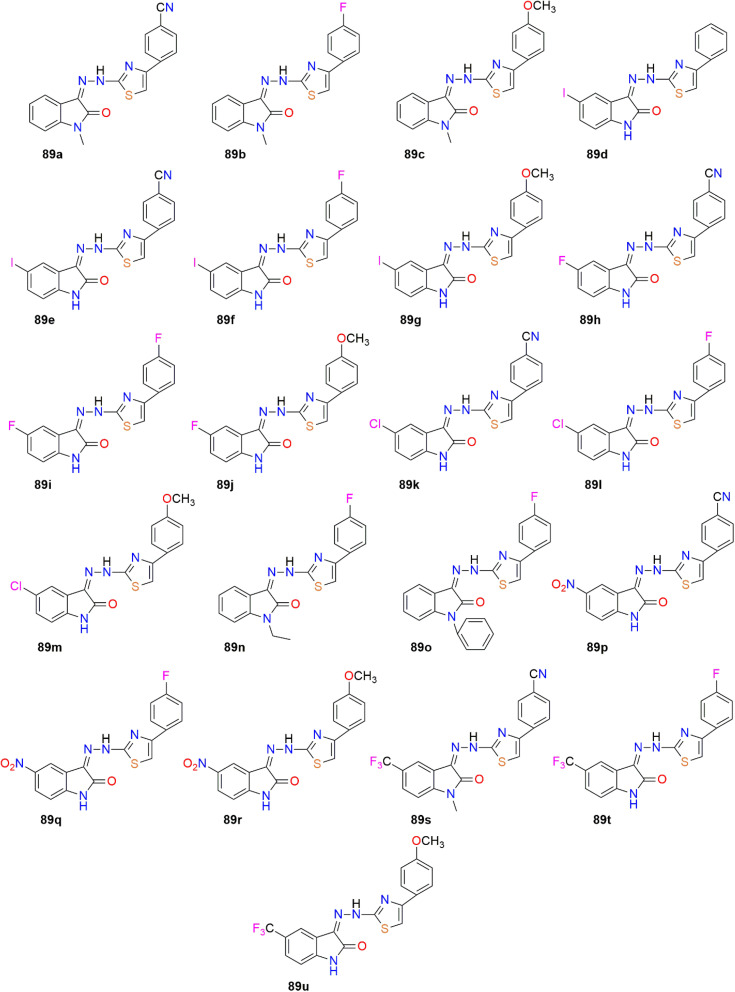
Structures of the hydrazino-thiazoles 89a–u.

89a–97 were screened *in vitro* by DPPH FRSA to check their potential as hydrogen radical donors or free radical scavengers using Ascorbic acid as drug. 89i, and 92 were highly potent (IC_50_ = 5.02, 8.54 µg mL^−1^) than Ascorbic acid (IC_50_ = 9.01 µg mL^−1^). 89a–c, 89g–h, 89k–l, 89n, and 89q–r (IC_50_ = 11.76, 18.09, 18.43, 17.36, 22.74, 32.7, 31.65, 32.42, 35, 18.53 µg mL^−1^) served as moderate to good anti-oxidants. *N*-alkyl isatins showed better FRSA (IC_50_ = 11.76, 18.09, 18.43, 78.24 µg mL^−1^) than *N*-benzyl isatin (IC_50_ = 303 µg mL^−1^). Among 5-halo isatins (I, F, Cl), fluoro group showed dominant anti-oxidant potential. Among phenacyl bromides, anti-oxidant potential was F > OCH_3_ > CN. 89i with two fluoro groups, increased FRSA (IC_50_ = 5.02 µg mL^−1^) than drug (Fig. 31).^61^

**Scheme 8:**
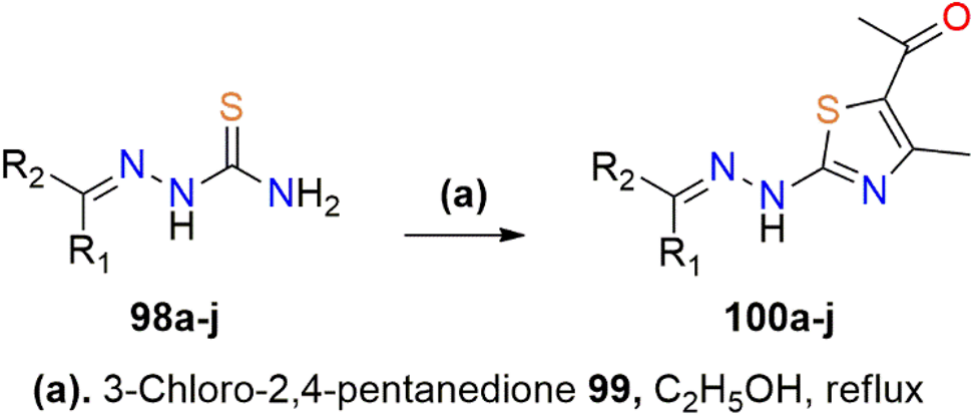
Synthesis of (thiazol-5-yl)ethan-1-ones 100a–j.

Mohammed Adnan Abid synthesized ten (thiazol-5-yl)ethan-1-ones (Schemes 8). Thus, thiosemicarbazone compounds 98a–j, and 3-chloro-aceyl acetone 99 dissolved in ethanol, were refluxed for 4–7 h. Upon completion, ice was added to get precipitates of the target (thiazol-5-yl)ethan-1-ones 100a–j in good yields.^62^

**Fig. 32:**
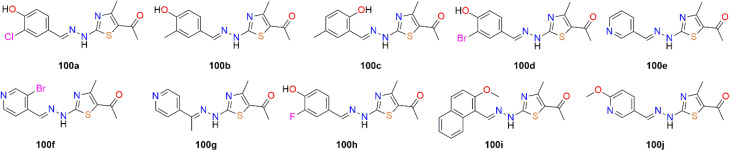
Structures of the synthesied (thiazol-5-yl)ethan-1-ones 100a–j.

100a–j were screened as potential antidiabetics using Acarbose as drug (IC_50_ = 16.04 µg mL^−1^). 100i served as highly potent inhibitor of α-amylase enzyme (IC_50_ = 18.68 µg mL^−1^), conversely, 100e–f served as least potent inhibitors (IC_50_ = 43.08, 138.60 µg mL^−1^), 100b–c, and 100i were almost equipotent (IC_50_ = 19.05, 18.02. 18.68 µg mL^−1^), in comparison to Acarbose. Against α-glucosidase, 100i, and 100c showed the highest inhibitions (IC_50_ = 20.25, 22.63 µg mL^−1^), and 100e emerged as the least potent (IC_50_ = 122.75 µg mL^−1^) compared to Acarbose (IC_50_ = 33.77 µg mL^−1^). In Dipeptidyl Peptidase IV (DPP-IV) inhibition, most of the inhibitors showed lower inhibitions, however 100i, 100c, and 100b showed average inhibition (IC_50_ = 23.81, 24.42, 28.11 µg mL^−1^), relative to Vildagliptin (IC_50_ = 3.39 µg mL^−1^) (Fig. 32).^62^

MD analysis was performed against α-glucosidase (lOGS), DPP4-inhibitor (3OMG), and α-amylase (lPPI) using Acarbose as drug. lOGS + 100i received highest affinity (−7.9 kcal mol^−1^), with non-conventional hydrogen interaction, and supportive electrostatic, as well as hydrophobic contacts. lOGS + 100c (−7.9 kcal mol^−1^), hydrogen bonds are present in A:ARG170:HE-A:UNL:O1, and A:HIS495:HD-A:UNL:N1, and in lOGS + 100g (−7.9 kcal mol^−1^), as ARG170 (HH21) interacting with UNL1 (N3), HIS451 (HD1) engaging UNL1 (O1), and UNL1 (H12) forming a hydrogen bond with TYR40 (OH). In lOGS + 100b system, the ligand achieved a binding affinity of −7.8 kcal mol^−1^, stabilized through hydrogen-bond interactions between ARG170 (HH21), and UNL1 (N3), HIS451 (HD1), and UNL1 (S1), and UNL1 (H12), and THR30 (O). In lOGS + 100d system, the ligand achieved binding affinity of −7.7 kcal mol^−1^, stabilized through hydrogen-bond interactions involving ARG170 (HH21) with UNL1 (N3), and UNL1 (H12) with THR30 (O), and lOGS + 100f system (−7.7 kcal mol^−1^), featured two key hydrogen bonds: ARG170 (HE) engaging UNL1 (O1), and HIS495 (HD1) interacting with UNL1 (N1). For lOGS + 100a, a binding affinity of −7.6 kcal mol^−1^ was observed, stabilized through hydrogen-bond interactions involving ARG170 (HH2) with UNL1 (S1), HIS451 (HD1) with UNL1 (N3), and UNL1 (H4) with SER38 (OG). For lOGS + 100h complex, ligand exhibited binding affinity of −7.3 kcal mol^−1^, stabilized through multiple hydrogen-bond interactions: ARG285 (HH21) with UNL1 (O1), ARG285 (HH22) with UNL1 (O1), TRP312 (HE1) with UNL1 (O1), ALA318 (HN) with UNL1 (O1), and UNL1 (H5) with LEU314 (O). In lOGS + 100j complex, ligand exhibited a binding affinity of −7.2 kcal mol^−1^, stabilized through several hydrogen-bond interactions: TYR40 (HH) with UNL1 (O2), ARG170 (HE) with UNL1 (N4), and UNL1 (H5) with GLN169 (OE1). For lOGS + 100e, lowest binding affinity of −7.0 kcal mol^−1^ was recorded, arising from a lone hydrogen bond formed between CYS4 (HN), and UNL1 (O1). In the lOGS + Acarbose system, a binding affinity of −7.5 kcal mol^−1^ was observed, supported by multiple hydrogen-bond interactions: ARG285 (HH22)-UNL1 (O), HIS365 (HD1)-UNL1 (O), ARG463 (HH12)-UNL1 (O), UNL1 (HA)-PHE316 (O), UNL1 (H)-ARG463 (O), and UNL1 (H)-ASP443 (O). In comparison, 100i (−7.9 kcal mol^−1^), showed superior α-amylase inhibitory potential relative to the drug.^62^

In DPP IV (3OMG) complex, 100a engaged Lys554 as the principal binding site for inhibition. Strongest binding affinity (−8.4 kcal mol^−1^) was achieved in the 3OMG + 100a system, supported by hydrogen-bond interactions between LYS512 (HN), and UNL1 (O2), LYS512 (HZ2), and UNL1 (N3), LYS512 (HZ3), and UNL1 (N1), UNL1 (H6), and ARG560 (O), and UNL1 (H4), and ASP545 (OD1). 3OMG + 100b achieved binding affinity of −8.1 kcal mol^−1^, with stabilization arising from electrostatic interactions in combination with multiple hydrophobic contacts. In 3OMG + 100i, binding energy of −8.0 kcal mol^−1^ was observed, with stabilization arising from a hydrogen bond formed between UNL1 (H4), and PRO510 (O), and 3OMG + 100c, hydrogen-bond interactions were observed between TYR547 (HH), and UNL1 (O2), as well as UNL1 (H5), and GLU205 (OE1). In 3OMG + 100h system, binding energy of −7.8 kcal mol^−1^ was observed, driven by hydrogen bond between UNL1 (H5), and ASN170 (OD1). Meanwhile, 3OMG + 100g complex was stabilized through multiple hydrogen bonds: GLN123 (HE22) with UNL1 (N2), GLN123 (HE22) with UNL1 (N4), and ASN151 (HD21) with UNL1 (O1). Hydrogen bonds were absent in the 3OMG + 100j complex (−7.5 kcal mol^−1^) as well as in 3OMG + 100d, 3OMG + 100e, and 3OMG + 100f (−7.3 kcal mol^−1^), though stabilization was achieved through favorable electrostatic contacts and multiple hydrophobic interactions. Apart from 100a, Acarbose demonstrated superior affinity toward 3OMG (−8.1 kcal mol^−1^), supported by hydrogen-bond interactions between MET509 (HNA), and UNL1 (F), THR565 (HG1), and UNL1 (F), and UNL1 (H) and PRO510 (O). Thus, decreasing order of mean affinity is: 100i: −8.0 > 100c: −7.9 > 100a = 100b: −7.867 > 100g: −7.667 > 100a: −7.533 > 100d: 7.433 > 100f: −7.4 > 100j: −7.300 > 100e0: −7.167 kcal mol^−1^.^62^

100a–j were subjected to *in vitro* anti-oxidant DPPH FRSA to check their ability to neutralize free radicals, and subsequent oxidative stress. Thus, varied anti-oxidant effects were obtained among the screened inhibitors with 100i emerged as highest DPPH free radical scavenger (IC_50_ = 2.50 µg mL^−1^), indicating strongest anti-oxidant potential. 100f showed the lowest DPPH free radical scavenging potential (IC_50_ = 32.36 µg mL^−1^), suggesting relatively weaker anti-oxidant effects compared to Ascorbic acid (IC_50_ = 2.09 µg mL^−1^).^62^

**Scheme 9:**
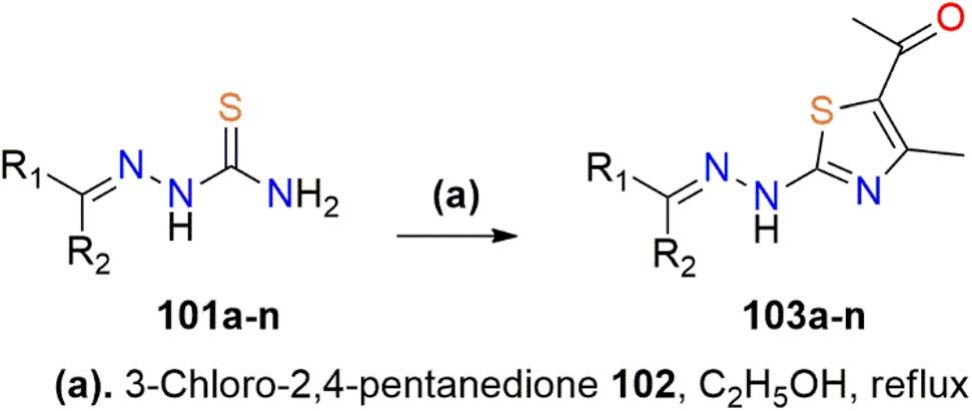
Synthesis of 5-acetyl-2-(benzylidenehydrazin-1-yl)-4-methyl-1,3-thiazoles 103a–n.

H. Mehmood *et al.* synthesized fourteen hydrazinyl-thiazole hybrids 103a–n. Thus, thiosemicarbazone compounds 101a–n, and 3-chloro-2,4-pentanedione 102 were refluxed in ethanol for 5–7 h to get hydrazinyl thiazoles 103a–n in good yield (66–82%) (Schemes 9).^63^

**Fig. 33:**
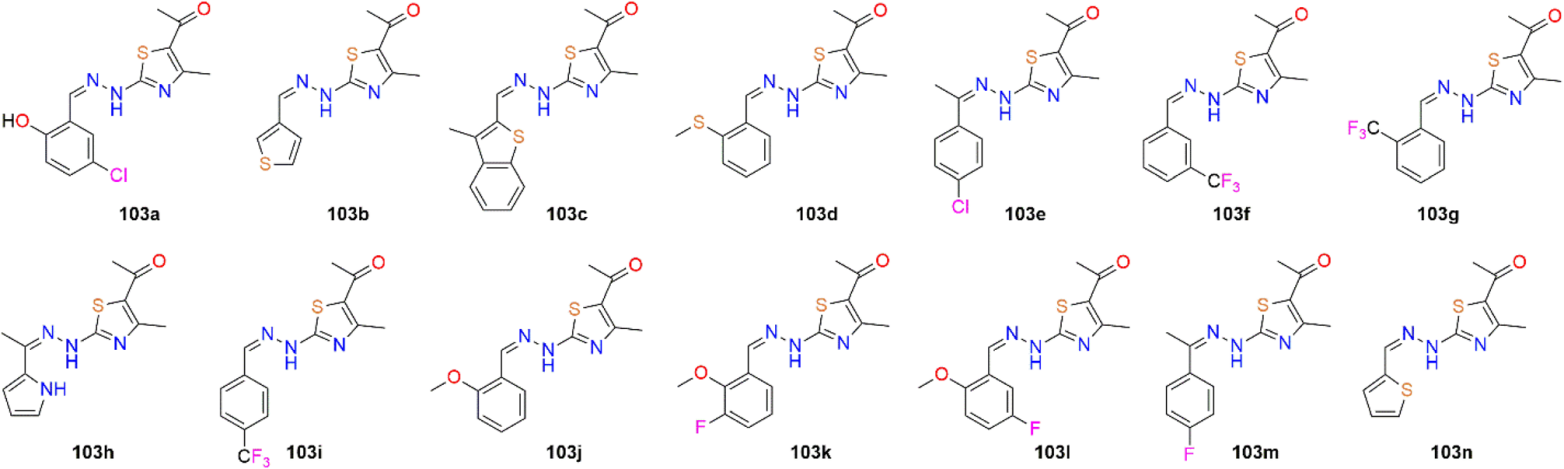
Structures of 5-acetyl-2-(arylidenehydrazin-1-yl)-4-methyl-1,3-thiazoles 103a–n.

103a–n are potent α-amylase inhibitors compared to Acarbose (IC_50_ = 5.62 ± 0.04 µM). 103e showed better inhibition (IC_50_ = 4.79 ± 0.08 µM). 103a (IC_50_ = 4.90 ± 0.09 µM), 103c (IC_50_ = 4.80 ± 0.07 µM), 103d (IC_50_ = 4.86 ± 0.19 µM), 103f (IC_50_ = 4.88 ± 0.07 µM), 103g (IC_50_ = 4.92 ± 0.15 µM), 103k (IC_50_ = 4.93 ± 0.03 µM), 103l (IC_50_ = 4.97 ± 0.06 µM), and 103n (IC_50_ = 4.97 ± 0.07 µM) were more potent than Ascorbic acid. 103b, 103h–I, and 103m (IC_50_ = 5.39 ± 0.09, 5.53 ± 0.10, 5.49 ± 0.15, 5.12 ± 0.08 µM) showed better inhibition than Acarbose. 103f–g, and 103i with trifluoromethyl group served as variable inhibitors (IC_50_ = 4.88 ± 0.07, 4.92 ± 0.15, 5.49 ± 0.15 µM). Minor changes in IC_50_ values of 103c–d, 103h, and 103n were due to different aromatic rings at hydrazine-1-yl moeity. Mono and di-substituted phenyl rings affected inhibitions as in 103a, 103e, 103k–m(Fig. 33).^63^

103a–n were screened to assess their antiglycation potential. Thus, good to moderate glycation inhibition (IC_50_ = 2.353 ± 0.008–3.413 ± 0.001 mM) was exhibited compared to amino guanidine drug (IC_50_ = 2.353 ± 0.044 mM). 103g showed least (2.353 ± 0.008 mM), 103b, 103e–f, and 103h good inhibition (IC_50_ = 2.54 ± 0.004, 2.53 ± 0.008, 2.52 ± 0.003, 2.53 ± 0.017 mM). Inhibitory potential of 103f, 103e, 103h, and 103b was comparable to reference drug. To conclude, concentration dependent antiglycation effects were observed, which increased with increase in concentration of the inhibitor.^63^

103a–n were docked into the active site of human pancreatic α-amylase (pdb: 4W93) in comparison to Acarbose as drug. 103e being the most potent, was stabilized by π–cation, π–sulphur, π–π stacking, and hydrogen bond. Acarbose drug lacks π–π stacking, and π-sulphur interaction with the target protein. π-Electrons of thiazole ring formed π–cation interactions with His201 (2.54 Å), π electrons of His201 form π–sulphur interactions with thiazole (5.11 Å), oxygen of acetyl group as hydrogen bond acceptor forms strong hydrogen bond with –NH of main chain amide of Ile235 (2.00 Å). Alkyl group of Ile235 forms π-sigma interactions with π-electronic cloud of thiazole ring (3.98 Å). Chlorophenyl ring of 103e forms π–sigma interactions with Leu135 (3.96 Å). Leu162 forms π-alkyl interactions with π–electrons of thiazole (5.28 Å). Acarbose, His201 forms hydrogen bonds with two OH groups, lacking π–sulphur, π–sigma, π–alkyl, π–cation, and π–π interactions. 103c being the 2^nd^ most potent, forms hydrogen bond with His201, and acyl oxygen (2.0 Å), hydrazine-1-yl moiety, and carboxylate oxygen of Asp197 (2.7 Å). 103c showed π–anion interaction. π-Electrons of thiazole forms π–π interaction with carboxylate oxygen of Glu233 (3.84 Å). 103c due to thiazole ring, showed π–π stacking with Trp59 (5.02, 4.28 Å). π–Electrons of thiazole forms π–alkyl interactions with Ala198 (5.12 Å). Molecular docking analysis revealed 103e (−7.9 kcal mol^−1^), and 103c (−8.1 kcal mol^−1^), showed stronger binding with the enzyme than reference drug.^63^

103a–n were subjected to in *in vitro* DPPH free radical scavenging assay, compared to Ascorbic acid. Thus, 103c–d, 103i–j, 103l, and 103n were average to good anti-oxidants (IC_50_ = 10.81 ± 0.02–16.10 ± 0.03 mM) compared to Ascorbic acid (6.70 ± 0.01 mM). The remaining inhibitors 103a–b, 103e–h, 103k, and 103m exhibited less than 50% anti-oxidant effects. At low conctration, no significant anti-oxidant potential was observed, and at higher concentration, no inhibitor displayed inhibition well above Ascorbic acid.^63^

**Scheme 10:**
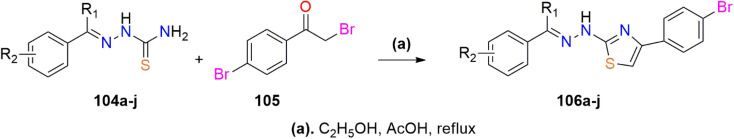
Synthesis of 1,3-hydrazinylthiazoles 106a–j.

A. Ghafoor *et al.* synthesized ten differently substituted hydrazinyl thiazoles 106a–j (Scheme 10). Thus, thiosemicarbazone compounds 104a–j, and phenacyl bromide 105 in ethanol/dioxane were refluxed for 3–8 h. Upon completion, the product was centrifuged, washed, and dried to get 106a–n.^64^

**Fig. 34:**
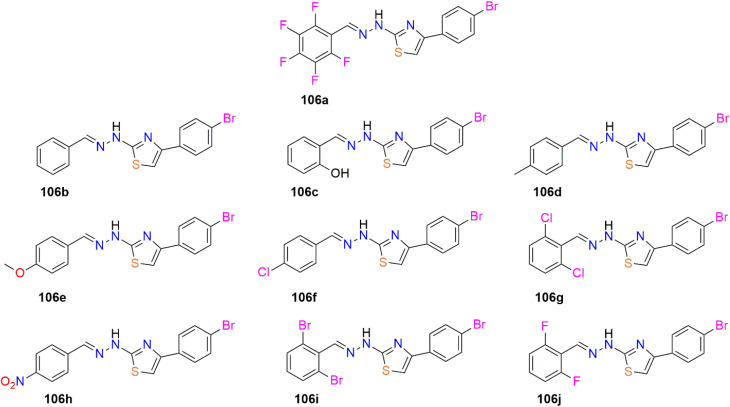
Structures of the screened 1,3-thiazoles 106a–j.

106a–j were screened for DPPH (10.2±2–92 ± 2%), and ABTS FRSA (14±2–91 ± 2%) using BHT (IC_50_ = 5.086, 5.083 mg mL^−1^), and Gallic acid (IC_50_ = 5.081, 5.078 mg mL^−1^) as standards. 106i–j showed higher DPPH inhibition than standard anti-oxidants BHT, and Gallic acid. 106c–d, 106f, and 106j showed highest ABTS inhibition than standard BHT. 106i showed strong ability (92%) against DPPH than standard BHT (85%). 106c, 106d, 106f, and 106j showed maximum FRSA (91%, 91%, 78%, 74%) than standard BHT. 106c was termed as the least potent inhibitor against DPPH (10.2%), and ABTS (14%) free radicals. In terms of IC_50_ values, 106i, and 106a expressed higher DPPH free radicals inhibition (IC_50_ = 4.949, 4.884 mg mL^−1^), than standard BHT (IC_50_ = 5.083 mg mL^−1^). 106a, 106c, 106d, and 106f showed higher inhibition (IC_50_ = 4.967, 4.943, 4.944, 4.984 mg mL^−1^) for ABTS than standard BHT DPPH (IC_50_ = 5.086 mg mL^−1^) (Fig. 34).^64,65^

**Scheme 11:**
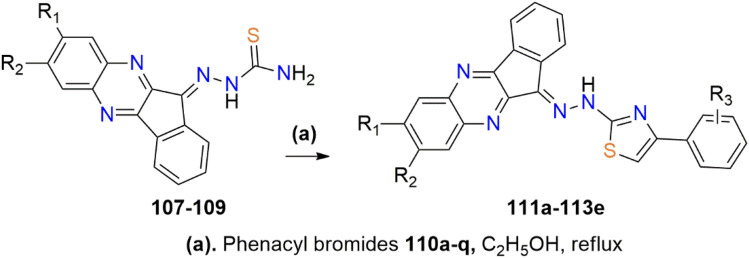
Synthesis of indenoquinoxalines linked hydrazinyl thiazoles 111a–113e.

S. Hameed *et al.* synthesized thirty indenoquinoxalines linked hydrazinyl thiazoles (Scheme 11). Thus, thiosemicarbazone compounds 107–109 refluxed phenacyl bromides 110a–q in ethanol to yield indenoquinoxalines linked thiazole hybrids 111a–q, 112a–h, 113a–e.^66^

**Fig. 35:**
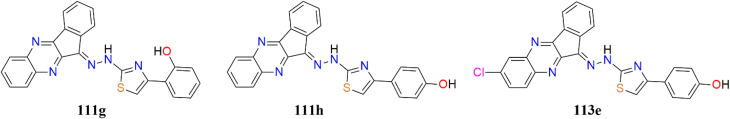
Structures of potent indenoquinoxalines linked hydrazinyl thiazoles.

111a–q, 112a–h, and 113a–e were screened for *in vitro* antidiabetic potential using Acarbose as standard (IC_50_ = 13.5 ± 0.2 µM). Thus, potent to moderate inhibition was noted against both enzymes *i.e.*, α-amylase (IC_50_ = 0.3–76.6 µM), and α-glucosidase (IC_50_ = 1.1–92.2 µM). Limited SARs suggest, 111h (IC_50_ = 0.6 ± 0.0, 1.4 ± 0.1 µM), 111g (IC_50_ = 1.3 ± 0.2, 3.0 ± 0.2 µM), and 113e (IC_50_ = 6.5 ± 0.1, 10.3 ± 0.1 µM), as potent inhibitors (Fig. 35).^66^

**Fig. 36:**
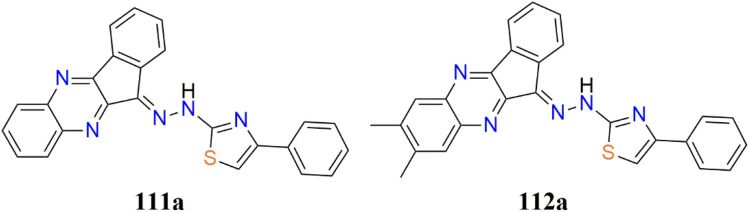
Structures of moderate indenoquinoxalines linked hydrazinyl thiazoles.

111a (IC_50_ = 29.5 ± 0.2, 33.9 ± 0.3 µM), and 112a (IC_50_ = 12.5 ± 0.1, 20.1 ± 0.1 µM) exhibited moderate effects (Fig. 36).^66^

**Fig. 37:**
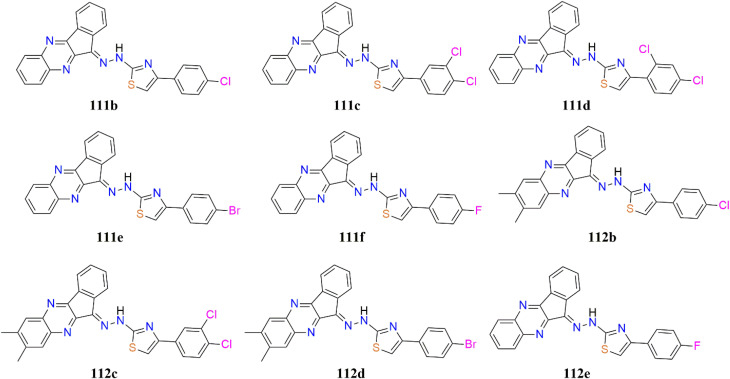
Structures of indenoquinoxalines linked hydrazinyl thiazoles as moderate dual inhibitors.

In hybrids bearing halogen-groups, 111b–f, 112b–e, and 113d possess moderate inhibitions against both enzymes (Fig. 37).^66^

**Fig. 38:**
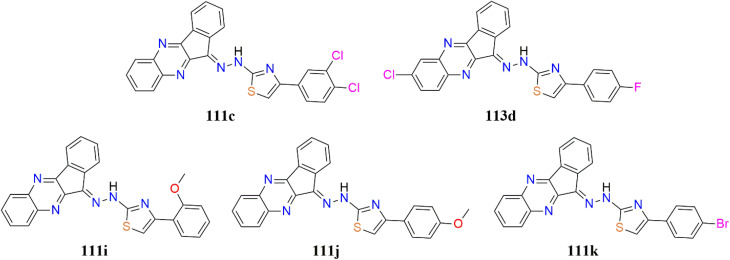
Structures of potent indenoquinoxalines linked hydrazinyl thiazoles.

111c (IC_50_ = 19.6 ± 0.4, 27.8 ± 0.2 µM), and 113d (IC_50_ = 14.1 ± 0.2, 24.4 ± 0.3 µM) showed better inhibitions. In 112i–k, 112k(IC_50_ = 13.1 ± 0.1, 21.6 ± 0.1 µM) emerged as potent inhibitors compared to 112i–j (Fig. 38).^66^

**Fig. 39:**
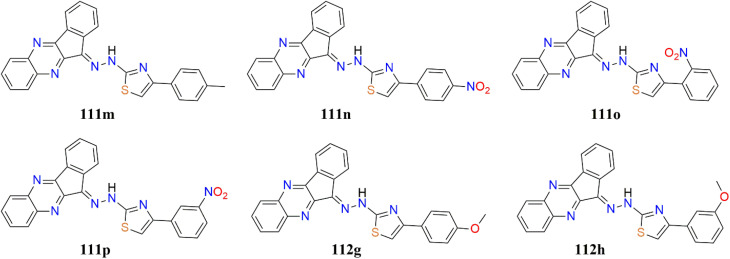
Structures of potent indenoquinoxalines linked hydrazinyl thiazoles.

111m (IC_50_ = 12.7 ± 0.2, 19.6 ± 0.4 µM) showed similar effects as drug. 112g (IC_50_ = 20.2 ± 0.2, 29.3 ± 0.1 µM) showed better inhibition then 112h (IC_50_ = 44.8 ± 0.2, 56.1 ± 0.2 µM) against both enzymes. 111o (IC_50_ = 13.1 ± 0.1, 21.6 ± 0.1 µM) showed better inhibition in reference to 111n (IC_50_ = 30.0 ± 0.2, 42.8 ± 0.2 µM), and 111p (IC_50_ = 28.6 ± 0.3, 27.6 ± 0.2 µM) (Fig. 39).^66^

In Acarbose, NH group binds to α-amylase primarily through hydrogen bonding with carbonyl oxygen (Glu233), and its broad binding affinity allows additional hydrogen interactions with residues such as His201, Gln63, His299, Arg195, Tyr151, Lys200, Ala198, Glu240, His101, and His305. 111m exhibited interaction (−6.5 kcal mol^−1^) with conserved water molecule, and hydrogen bond (Asp300). 111g, 111o, and 112a were stabilized (−6.5, −7.0, −8.2 kcal mol^−1^) primarily through hydrophobic contacts, and water-mediated bridges within the binding pocket. 113e, bearing halogen group, established hydrogen bonding interactions (−9.8 kcal/mol) with critical amino acid residues of α-amylase.^66^ Against α-glucosidase enzyme, the inhibitors demonstrated strong binding affinities, which can be attributed to their preferred orientations within the active site. Moderate inhibitors have reasonable hydrogen, and hydrophobic interactions. Binding profile of quinoxaline derivative accounts for the relatively modest inhibition. 111g–h exhibited same interaction (−17.6, −17.0 kcal mol^−1^), interacting Arg212, Thr215, and Arg439 inhibiting α-glucosidase. 111o, and 113e interact (−17.4, −16.6 kcal mol^−1^) critical residues within the cavity in a similar fashion. Molecular docking analysis suggests intact quinoxalines as promising targets for structural modifications.^66^

**Fig. 40:**
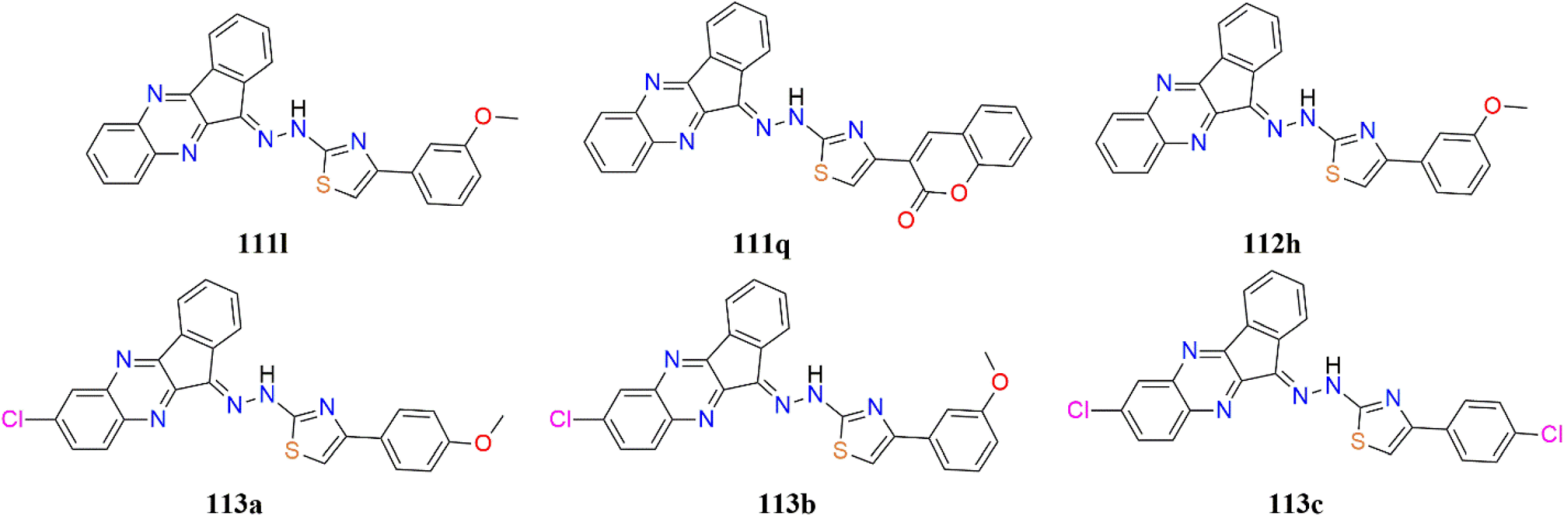
Structures of anti-oxidant indenoquinoxalines linked hydrazinyl thiazoles.

Anti-oxidant capability of the synthesized hybrids was assessed using Cupric reducing anti-oxidant capacity (CUPRAC), ferric reducing antioxidant power (FRAP), and DPPH quenching assay. 111g, 111h, 111o, and 113e showed better, and the remaining hybrids as moderate anti-oxidant potential. DPPH scavenging (SC_50_ = 7.6–125.9 µM) was measured using Ascorbic acid as refence (SC_50_ = 21.5 ± 0.2 µM). 111h served as the most potent inhibitor (SC_50_ = 7.6 ± 0.1 µM) as: 111h > 111g > 1110 > 113e > Ascorbic acid. 111a–q displayed significant to moderate DPPH FRSA (SC_50_ = 10.3–125.9 µM). 111g–h (SC_50_ = 15.5 ± 0.1, 10.3 ± 0.1 µM) were potent in reference to drug. 111o (SC_50_ = 19.4 ± 0.2 µM) has higher inhibition compared to drug. 112a–h showed less DPPH FRSA. 113a–e further decreased activity compared to Ascorbic acid (Fig. 40).^66^

**Scheme 12:**
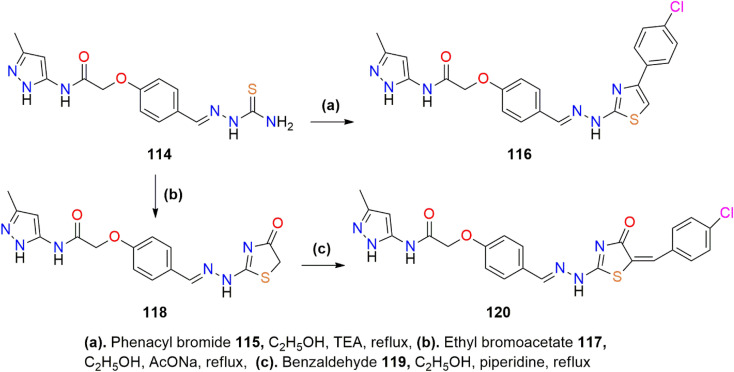
Synthesis of pyrazole based hydrainyl thiazole hybrids 116-120.

Alia Abdulaziz Alfi, A. Alharbi, J. Qurban *et al.* synthesized three hydrazinyl thiazoles (Scheme 12). Thus, ethanolic solution of thiosemicarbazone compound 114, TEA, and 4-chlorophenacyl bromide 115 were refluxed in ethanol for 2 h, to pyrazole-thiazole 116. Ethyl bromoacetate 117 was poured into a suspension of thiosemicarbazone compound 114, and fused sodium acetate in ethanol at reflux for 4 h, cooled, and recrystallized from EtOH-DMF. Later, 4-chlorobenzaldehyde 119, and thiazolinone 118 in ethanol, were added piperidine, at refluxed for 2 h. The solid was collected, washed, and dried to get pyrazole-based thiazolinone (120).^67^

**Scheme 13:**
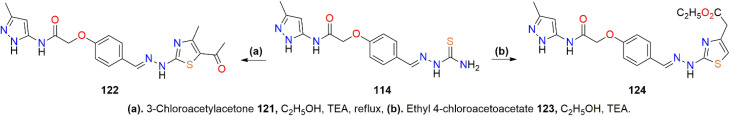
Synthesis of pyrazole based hydrainyl thiazole hybrids 122, 124.

Similarly, thiosemicarbazone compound 114, and TEA in ethanol, were added into 3-chloroacetylacetone 121/ethyl 4-chloroacetoacetate 123 at reflux for 4 h. Upon completion of the reaction, pyrazole-based hydrazinylthiazoles 122, 124 were dried, and recrystallized from THF (Scheme 13).^67^

DPPH stabilizes free radicals through proton or electron donation. Anti-oxidant efficiency of pyrazole based hydrazinyl thiazole hybrids 116, 118, 120, 122, and 124 was compared to Butylated hydroxytoluene (BHT), and Ascorbic acid as drugs (IC_50_ = 25.46 ± 0.11, 22.05 ± 0.03 µg mL^−1^). 118, 120, and 124 showed higher inhibition (IC_50_ = 23.15–29.83 µg mL^−1^). 116, and 122 showed reasonable inhibition (IC_50_ = 33.27, 36.34 µg mL^−1^). SARs of pyrazole based hydraziny thiazole hybrids augment 118, 120, and 124 showed highest anti-oxidant activity. 118 contains hydrazo moiety, showed significant activity. 124 has hydrazo linkage, and ester group, which imparts anti-oxidant effects. 118 possess arylidene moiety, and have notable anti-oxidant effects.^67^ Molecular docking analysis was performed on Urate oxidase crystal structure as nominated protein (PDB-1R4u). 116 with thiazolinone ring (−7.0788 kcal mol^−1^) established hydrogen bond (H-donor) through S atom of thiazole with Glu 272 (3.43 Å), and hydrogen bond (H-acceptor) through O atom of amide with Arg 176 (3.27 Å), and thiazolyl ring displayed π-H with Arg 275 (4.27 Å). 118 with thiazolinone ring (−7.6003 kcal mol^−1^) established four hydrogen acceptors using O atom of amide, and Ile 177 by (3.06 Å), O atom of thiazolone ring, and Val 227 (3.31 Å), O atom of thiazolone ring, and Arg 176 (3.56 Å), and N-atom of thiazolone ring, and Arg 176 (3.34 Å). 120 with chlorine developed (−7.4130 kcal mol^−1^) two π-cation interactions with Arg 275, using pyrazolyl, and phenoxy rings (4.86, 4.47 Å). 122 (−6.9222 kcal mol^−1^) developed hydrogen-donor interaction with N_2_-atom of pyrazolyl ring, and Asn 254 (3.29 Å), π-H interactions of pyrazolyl ring with Arg 176 (3.96 Å), and phenoxy ring with Glu 259 (3.77 Å). 124 with ester group, exhibited good binding score (−7.5487 kcal mol^−1^), hydrogen-donor bonds among S-atom of thiazole ring with Glu 259 (2.98 Å), and Gly 272 (3.23 Å), as well as N-atom of hydrazo moiety with Glu 259 (3.42 Å). In comparison, Ascorbic acid developed (−4.8533 kcal mol^−1^) hydrogen-donor bonds among two O atoms of 1^st^, and 2^nd^ hydroxyl groups with Ile 177 (3.04, 3.19 Å). Thus, MD analysis augments 118, 120, 124 received higher binding scores for amino acid 1R4u, which correlate well with the obtained *in vitro* anti-oxidant assay.^67^

**Scheme 14:**
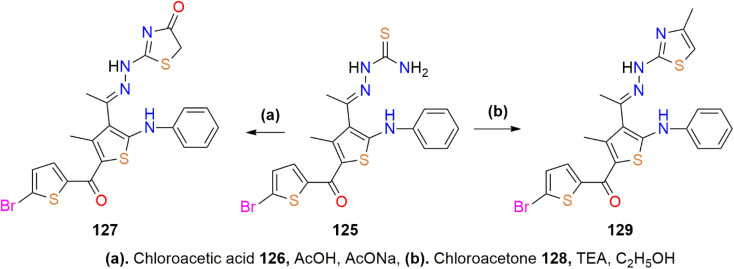
Synthesis of thiophenyl thiazole hybrids 127, 129.

I. Althagaf synthesized two thiophenyl thiazole hybrids 127, 129(Scheme 14). Thiosemicarbazone compound 125, and chloroacetic acid 126 were added fused sodium acetate, and acetic acid, at reflux for 4 h. The solid obtained was recrystallized from dioxane to thiophenyl thiazolin-4-one 127. Thiosemicarbazone compound 125 dissolved in ethanol, was added chloroacetone 128, and TEA, at reflux for 4 h. The solid obtained was filtered to get thiophenyl thiazole hybrid 129.^68^

127, 129 possess notable anti-oxidant effects using Ascorbic acid as drug. In terms of % inhibitions at three different concentrations (1.20, 2.40, 4.80 µg mL^−1^), 127 exhibited dose dependent superior inhibitions (13.18, 16.36, 18.11%) followed by 129 (8.15, 10.44, 12.61%), and Ascorbic acid (3.31, 4.17, 7.22%). In terms of their IC_50_ values, di-2-thienyl ketone hydrazinyl-thiazole 129, and di-2-thienyl ketone hydrazinyl-thiazolinone 127 showed significant inhibitions (IC_50_ = 34.37, 39.85 µM) compared to Ascorbic acid (IC_50_ = 125.43 µM). Structurally, 127 contain thiazolinone and 129 contain thiazole rings in their structures, responsible for their excellent anti-oxidant effects.^68^

Molecular docking analysis was performed for inhibitors 127, and 129 against cytochrome c peroxidase (PDB Code: 2AS1). Cytochrome c peroxidase is a potent mitochondrial anti-oxidant that inhibits the production of hydrogen peroxide. Briefly, di-2-thienyl ketone hydrazinyl-thiazolinone 127 established two different interactions (hydrogen bonding, π–π stick), and received significant binding energy (−9.0969 kcal mol^−1^). Intermolecular hydrogen bond was established between S-atom of thiazolinone ring, and Asp 37 (3.43 Å), and π–π stick binding was present between thiophene ring, and Trp 51 (3.94 Å). Di-2-thienyl ketone hydrazinyl-thiazole 129 received relatively higher binding energy (−9.6027 kcal mol^−1^), through hydrogen bonding between NH of anilino group, and Met 172 (3.42 Å), as well as π–π stick interaction exhibited between thiophene ring, and Trp 51 (3.34 Å). Ascorbic acid received lower binding energy (−4.6693 kcal mol^−1^), though five hydrogen bonds were observed between oxygen atoms, and surrounding residues (Pro 145, Met 172, Arg 48, His 51) of 2AS1. To conclude, this study suggested good agreement between the results obtained in *in vitro* evaluation*,* and *in silico* analysis.^68^

**Scheme 15:**
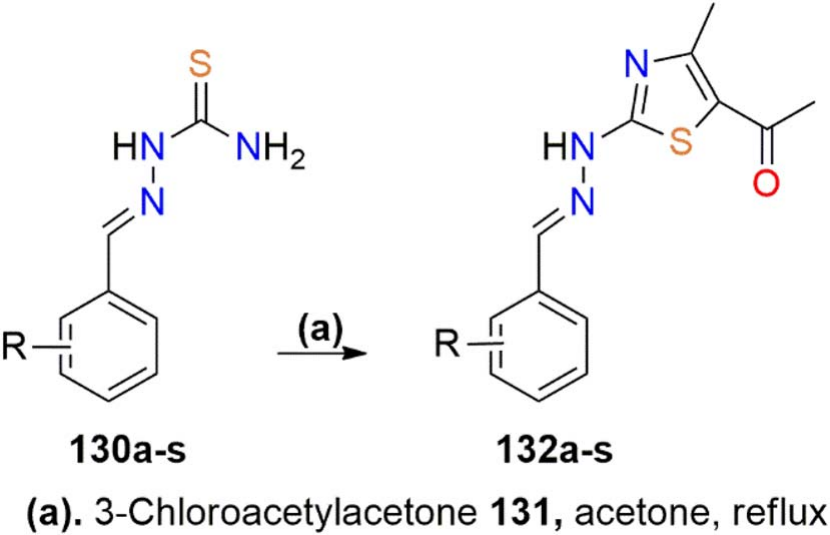
Synthesis of hydrazinyl thiazoles 132a–s.

Md. S. Shah, M. M. Rahman, Md. D. Islam *et al.* synthesized nineteen hydrazinyl thiazoles (Scheme 15). Thus, thiosemicarbazone compounds 130a–s, and 3-chloroacetylacetone 131 were refluxed in acetone at 60 °C for 2–5 h. Upon completion, the mixture was cooled, filtered, and recrystallized from ethanol to get 132a–s.^69^

**Fig. 41:**
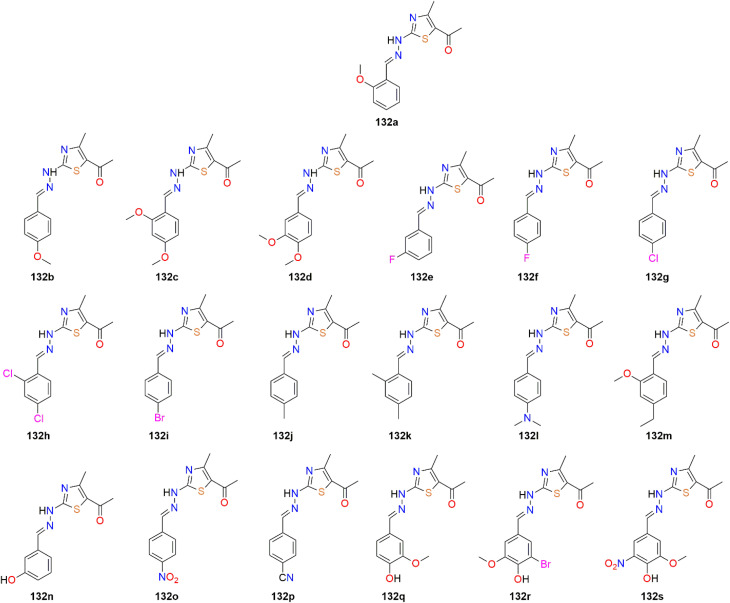
Structures of the hydrazinyl thiazoles 132a–s.

132a–s were subjected to *in vitro* DPPH FRSA, using Ascorbic acid as drug. Thus, % inhibition increased with concentration of drug, and inhibitors. The inhibitors showed excellent FRSA (>90%) (500 µg mL^−1^) except 132h, and 132a. In terms of IC_50_ values, 132a exhibited 9-fold higher anti-oxidant effects (IC_50_ = 3.52 ± 0.86 µg mL^−1^) than drug (IC_50_ = 27.34 ± 1.86 µg mL^−1^). 132b showed good anti-oxidant potential (IC_50_ = 41.65 ± 2.92 µg mL^−1^). 132c–d (IC_50_ = 44.56 ± 1.49, 85.89 ± 1.52 µg mL^−1^) proved detrimental showing FRSA because of steric factor. In halo substituted inhibitors, anti-oxidant effects decreased with increase in their size in 132f, 132g, and 132i (IC_50_ = 43.72 ± 3.19, 62.36 ± 3.20, 65.06 ± 5.64 µg mL^−1^). DPPH FRSA was subjected to size, and polarizability of halogens, which showed varied inhibitions with change in their positions. 132f (*para*-fluorine, IC_50_ = 43.72 ± 3.19 µg mL^−1^) was more favorable than 132e (*meta*-fluorine, IC_50_ = 75.08 ± 2.95 µg mL^−1^). 132h bearing *ortho*, *para*-chloro substitution showed higher anti-oxidant potential (IC_50_ = 33.90 ± 2.60 µg mL^−1^) than 132g (*para*-chlorine, IC_50_ = 62.36 ± 3.20 µg mL^−1^). Inhibition increased with increase in size of *para*-substituted alkyl group in 132j (*para*-methyl, IC_50_ = 27.89 ± 1.30 µg mL^−1^), and 132m (*para*-ethyl, IC_50_ = 23.78 ± 1.50 µg mL^−1^). 132k bearing *ortho*, *para*-methyl groups showed comparable inhibition to Ascorbic acid. 132l (*para-N*,*N*-dimethyl group) showed good potential (IC_50_ = 33.02 ± 2.41 µg mL^−1^). 132o, and 132p bearing electron withdrawing groups (Cyano, Nitro) possess marked decrease in oxidative potential (IC_50_ = 100.97 ± 3.08, 105.73 ± 5.80 µg mL^−1^). 132n showed weak (IC_50_ = 183.43 ± 2.26 µg mL^−1^), and 132q as moderate (IC_50_ = 48.30 ± 3.66 µg mL^−1^) anti-oxidant potential. Trisubstituted 132r, and 132s bearing electron donating, and withdrawing groups at *ortho-, meta-* and, *para*-positions were found to be associated with lower anti-oxidant potential (Fig. 41).^69^132a, being the most potent DPPH free radical scavenger (IC_50_ = 3.52 ± 0.86 µg mL^−1^), was analyzed in molecular docking analysis against human antioxidant enzyme receptor 3MNG. 132a received good binding affinity (−5.6 kcal mol^−1^). Arg95 established hydrogen bonding with carbonyl oxygen (3.27 Å), Ala90 (5.03 Å), and Leu96 (5.07 Å) were involved in hydrophobic interactions with the phenyl ring, as well as electrostatic interaction was present between Glu16, and thiazole ring (3.67 Å).^69^

**Scheme 16:**
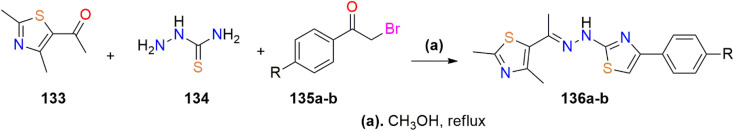
Synthesis of hydrazinyl thiazoles 136a–b.

V. A. Adole *et al.* synthesized two hydrazinyl thiazole hybrids 136a–b(Scheme 16). Equimolar 1-(2,4-dimethylthiazol-5-yl)ethan-1-one 133, thiosemicarbazide 134, and 2-bromo-1-(4-nitrophenyl)ethan-1-one 135a/4-(2-bromoacetyl)benzonitrile 135b were refluxed in methanol, and glacial acetic acid for 60 min. Once completed, the product was filtered, washed using ethanol, dried, and recrystallized from ethanol to yield 136a–b.^70^

**Fig. 42:**
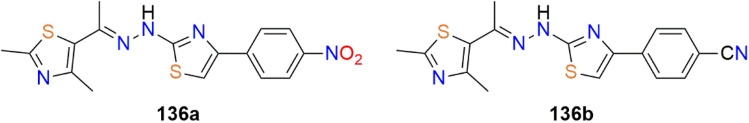
Structures of the synthesized hydrazinyl thiazoles 136a–b.

136a–b were screened in *in vitro* hydroxyl, and DPPH FRSA using Ascorbic acid as drug. In hydroxyl FRSA, 136a showed substantially higher FRSA (69.5%), compared to 136b which possess slightly lower FRSA (65.1%), than Ascorbic acid (84.5%). In DPPH FRSA, 136a showed lower (64.5%) FRSA, and 136b higher FRSA (71.5%), in reference to Ascorbic acid (83.1%). Thus, although the inhibitors exhibited lower anti-oxidant potential in comparision to Ascorbic acid as drug, yet possess notable % FRSA (Fig. 42).^70^

**Scheme 17:**
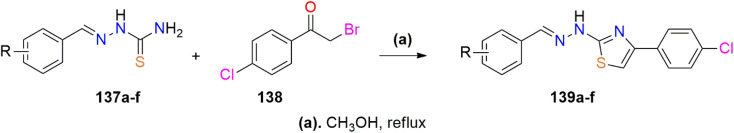
Synthesis of hydrazinyl thiazoles 139a–f.

Y. Zhu *et al.* synthesized six hydrazinyl thiazoles 139a–f(Scheme 17). Thus, equimolar thiosemicarbazone compounds 137a–f, 2′-bromo-4-chloroacetophenone 138, were refluxed in methanol for 4 h. Upon completion, cold water was added, stirred for 60 min, and the precipitates were filtered, washed with methanol to get hydrazinyl thiazoles 139a–f.^71^

**Fig. 43:**
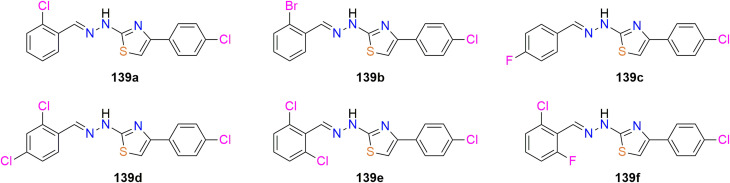
Structures of the potent hydrazinyl thiazoles 139a–f.

139a–f were evaluated for DPPH FRSA, using Ascorbic acid as drug. 139a–f showed % FRSA as: 90.84, 87.78, 90.06, 91.11, 84.07, and 89.22% (500 µg mL^−1^). 139d showed highest DPPH inhibition (91.11%). 139b–d, and 139f displayed over 85% DPPH FRSA (500 µg mL^−1^). In terms of their IC_50_ values, 139c served as potent (IC_50_ = 21.71 ± 2.64 µg mL^−1^), and 139e as weaker (IC_50_ = 36.44 ± 3.90 µg mL^−1^) anti-oxidants compared to Ascorbic acid (IC_50_ = 12.95 ± 3.15 µg mL^−1^). Halogens as electron withdrawing groups, enhanced anti-oxidant potential, influenced by size, and polarizability of the atoms. Anti-oxidant potential of 139c, 139a, and 139b (IC_50_ = 21.71 ± 2.64, 23.56 ± 3.39, 27.25 ± 3.61 µg mL^−1^) decreased, with decrease in halogens as EWGs. 139d (IC_50_ = 22.59 ± 4.26 µg mL^−1^), showed higher anti-oxidant potential than 139a. 139e (IC_50_ = 35.73 ± 3.90 µg mL^−1^) exhibited lower anti-oxidant potential, suggest steric hindrance lowers anti-oxidant potential (Fig. 43).^71^

139a–f were subjected to molecular docking analysis against human anti-oxidant enzyme receptor 3MNG. 139a–f showed better binding affinities (−5.42 to −6.37 kcal mol^−1^). 139a, being the most potent, received binding affinity of −6.31 kcal mol^−1^. Thus, two hydrogen bonds were found, as N13–H14⋯GLU27:O (oxygen atom of GLU27, 2.15 Å), S18⋯ASN24:H (ASN24, 2.33 Å). Hydrophobic interactions include residues LEU28, and VAL23 with π-electron of thiazole ring (5.38, 5.34 Å), LYS22 interacted with π-electron phenyl ring (4.32 Å). 139c with binding affinity of −5.42 kcal mol^−1^ served as next potent anti-oxidant. The resulting hydrogen bonds include, N13–H14⋯LYS22:O (oxygen atom of LYS22, 2.19 Å), ASN24:H⋯S18 (ASN24, 2.06 Å), GLU27:H⋯S18 (GLU27, 2.68 Å). Hydrophobic interactions include LYS22, VAL23, and LEU28 with π-electron of phenyl, and thiazole ring (4.68, 4.27, 5.27 Å). LEU28 formed π–sigma hydrophobic interaction with the phenyl ring (2.82 Å). 139b showed binding affinity of −6.37 kcal mol^−1^, with hydrogen bond as, N13–H14⋯GLU107:O (GLU107, 2.14 Å). 139d received binding affinity of −6.30 kcal mol^−1^. Hydrogen bond was found, as N13–H14⋯GLU27:O (GLU27, 2.27 Å). 139e showed binding affinity of −5.43 kcal mol^−1^ and hydrogen bond as ASN122:H⋯N21 (ASN122, 2.15 Å), followed by 139f(−5.61 kcal mol^−1^) with hydrogen bonds as N13–H14⋯GLU27:O (GLU27, 1.80 Å), ARG95:H⋯F33 (ARG95, 1.82 Å). Molecular docking suggests 139a–f possess binding ability to human anti-oxidant enzyme receptor, in accordance with *in vitro* anti-oxidant assay.^71^

**Scheme 18:**
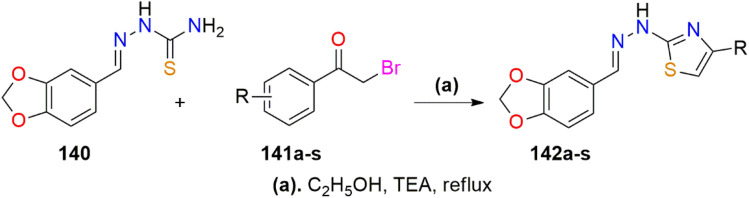
Synthesis of piperonal-based hydrazinyl thiazoles 142a–s.

A. Ahmad *et al.* synthesized nineteen hydrazinyl thiazoles 142a–s(Scheme 18). Thus, equimolar thiosemicarbazone compound 140, 2′-bromo-4-chloroacetophenones 141a–s, were refluxed in ethanol. Upon completion, cold water was added, precipitates were filtered, washed with water, and crystallized from ethanol to get hydrazinyl thiazoles 142a–s.^72^

**Fig. 44:**
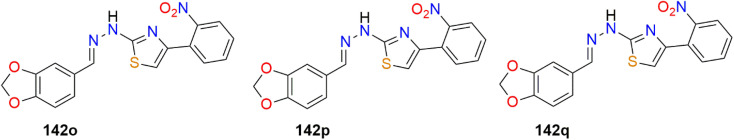
Structures of the most potent hydrazinyl thiazoles 142o–q.

142a–f served as strong to moderate inhibitors of α-glucosidase (IC_50_ = 1.59 ± 0.01–51.76 ± 0.02 µM), and α-amylase (IC_50_ = 3.39 ± 0.02–50.35 ± 0.02 µM) enzymes, using Acarbose (IC_50_ = 5.02 ± 0.02, 15.15 ± 0.01 µM) as drug. 142o emerged as the most potent inhibitor (IC_50_ = 1.59 ± 0.01, 5.72 ± 0.02 µM), 10-times, and 3-times higher than drug. Inhibition decreased slightly in 142q against both enzymes (IC_50_ = 2.27 ± 0.02, 8.56 ± 0.04 µM), still higher than drug. Similarly, inhibitory potential slightly decreased in 142p (IC_50_ = 2.68 ± 0.03, 11.08 ± 0.02 µM). Regio isomers 142o–q showed better inhibitions than Acarbose (Fig. 44).^72^

**Fig. 45:**
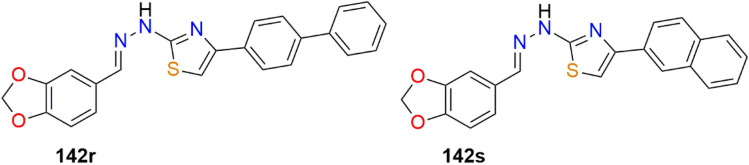
Structures of the least potent hydrazinyl thiazoles 142r–s.

142r–s showed decreased inhibitions against both enzymes. Biphenyl ring on 4^th^ position of thiazole in 142r decreased two-fold inhibitions (IC_50_ = 32.46 ± 0.03, 35.67 ± 0.04 µM) than drug. Naphthalene ring in 142s declined inhibition against both enzymes (Fig. 45).^72^

**Fig. 46:**
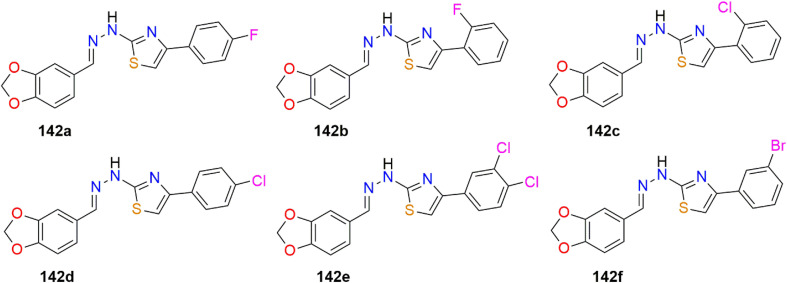
Structures of the halogen bearing hydrazinyl thiazoles 142a–f.

Inhibitors bearing electron withdrawing halogens, showed lower inhibition than drug. Among 142a–g, 142f (IC_50_ = 36.19 ± 0.02, 38.39 ± 0.02 µM), and 142g (IC_50_ = 38.13 ± 0.02, 40.15 ± 0.04 µM) were potent, and 142a–e as weak inhibitors (IC_50_ = 40.18–49.34, 37.48–50.13 µM) against both enzymes. Thus, inhibitors bearing electron withdrawing halogens showed 2–3 folds less inhibition than Acarbose (IC_50_ = 15.02 ± 0.02, 15.15 ± 0.01 µM) (Fig. 46).^72^

**Fig. 47:**
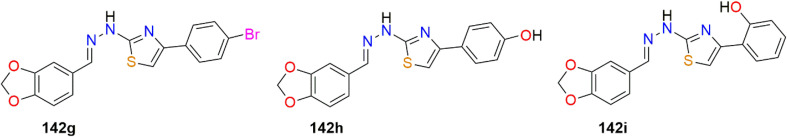
Structures of the EDGs bearing hydrazinyl thiazoles 142g–i.

Inhibitors with electron donating groups, 142h–i were more potent, with 142h as potent against both enzymes (IC_50_ = 7.43 ± 0.03, 3.39 ± 0.02 µM) compared to Acarbose. In 142i, inhibition decreased (IC_50_ = 15.81 ± 0.04, 16.12 ± 0.03 µM), though still comparable to Acarbose (Fig. 47).^72^

**Fig. 48:**
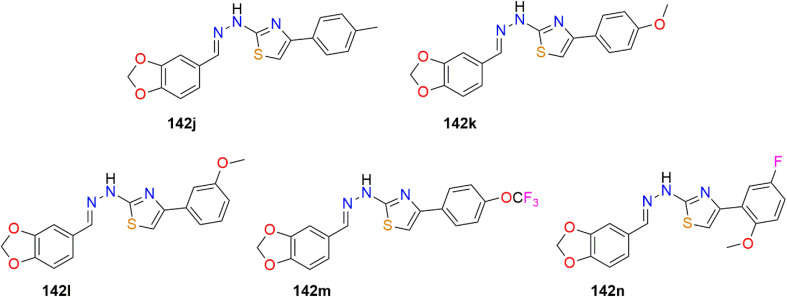
Structures of the weak hydrazinyl thiazoles 142g–i.

Inhibitions decreased in 142k (IC_50_ = 12.39 ± 0.03, 16.56 ± 0.02 µM) compared to 142h. In 142l (IC_50_ = 13.57 ± 0.04, 17.84 ± 0.03 µM), inhibitions were comparable against both enzymes. 142j showed sharp decrease in inhibition (IC_50_ = 27.45 ± 0.02, 24.87 ± 0.03 µM). Inhibitor 142n, showed much decreased inhibition (IC_50_ = 47.83 ± 0.04, 49.76 ± 0.03 µM). Similarly, 142m showed even more decrease in inhibitory potential (IC_50_ = 51.76 ± 0.02, 50.35 ± 0.02 µM) than 142n(Fig. 48).^72^

**Scheme 19:**
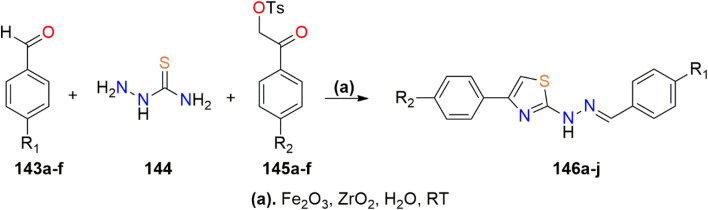
Synthesis of hydrazinyl thiazoles 146a–j.

A. Gurav *et al.* synthesized ten hydrazinyl thiazoles 146a–j. Thus, benzaldehydes 143a–f, thiosemicarbazide 144, and phenacyl tosylates 145a–f in water were stirred at room temperature. Upon completion, the products were recrystallized from ethyl acetate/ethanol (Scheme 19).^73^

**Fig. 49:**
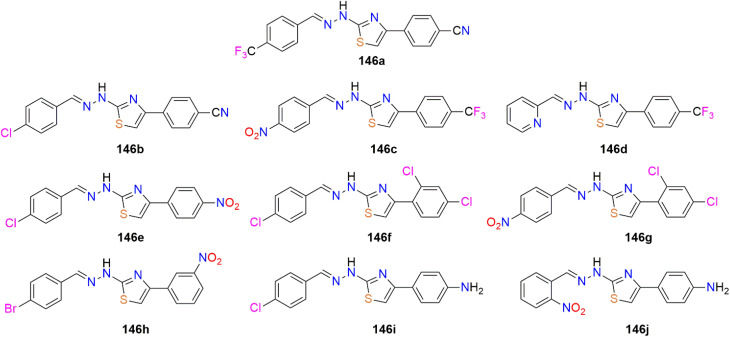
Structures of the synthesized hydrazinyl thiazoles 146a–j.

146c showed highest α-amylase inhibition (IC_50_ = 49.72 ± 0.8 µg mL^−1^) among 146a–e. Electron withdrawing groups (Trifluoromethyl, Nitro) improved lipophilicity, and hydrophobic interactions with active sites. Nitro group showed electrostatic interaction, and hydrogen bonding with α-amylase. 146a, showed moderate inhibition (IC_50_ = 54.86 ± 0.74 µg mL^−1^), suggesting significance of nitro group for enzyme inhibition, and 146b showed comparatively lower inhibition (IC_50_ = 55.18 ± 0.72 µg mL^−1^) than 146a. 146d–e (IC_50_ = 58.73 ± 0.34, 59.82 ± 0.43 µg mL^−1^), showed the least inhibitory potentials. Inhibition decreased in 146d, due to the lack of EWGs, and hydrophobic interactions. Similarly, decreased inhibitory potential of 146e than 146c, augments location, and combination of various groups as significant for enzyme inhibition (Fig. 49).^73^

Molecular docking analysis of 146a–e determined binding affinity using enzyme α-amylase (PDB:4W93) against Acarbose, and Metformin as drugs. 146a–e showed prominent binding, and docking scores (−8.4 to −9.3 kcal mol^−1^) than Acarbose, and Metformin (−7.7, −5.1 kcal mol^−1^). 146a exhibited binding potential (−9.3 kcal mol^−1^), with associated distinct interactions such as hydrogen bonding, π–alkyl, π–stacking, π–sigma, π–anion, and halogens (GLU233, LYS200, HIS201, ILE235, ALA198, LEU165, TRP59, ASP197, TYR62, GLN63). 146b–e developed notable π–alkyl interactions (ALA198, LEU165, LEU162), conventional hydrogen bonding (GLN63), π–sigma (ILE235), π–sulfur (HIS299, TRP58, TYR62), and π–stacking (TRP59, HIS201) (−8.9, −8.7, −8.4, −8.6 kcal mol^−1^). 146b–d showed salt bridge (ASP197, GLU233, TYR62). MD analysis suggests docked inhibitors have higher binding, and prominent interactions with active site residues than reference standards.^73^

**Scheme 20:**
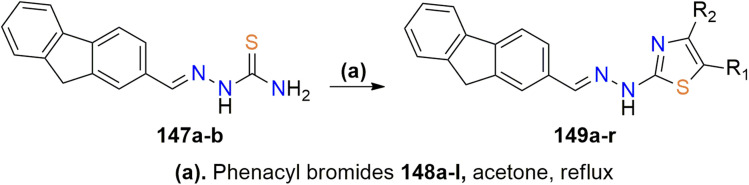
Synthesis of hydrazinyl thiazoles 149a–r.

S. S. Marufa *et al.* synthesized eighteen hydrazinyl-thiazoles 149a–r (Scheme 20). Thus, thiosemicarbazone compounds 147a–b, refluxed phenacyl bromides 148a–l in acetone at 60 °C. Upon completion, the mixture was cooled, filtered, and recrystallized from ethanol.^74^

**Fig. 50:**
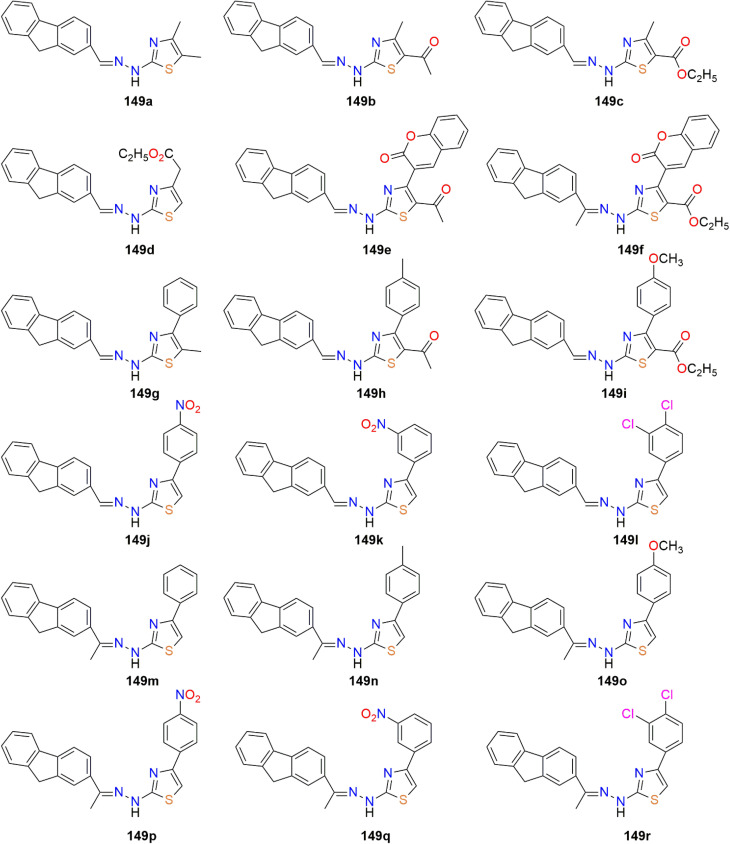
Structures of the synthesized hydrazinyl thiazoles 149a–r.

149a–r were evaluated in DPPH FRSA using Ascorbic acid (49.68 ± 3.68 µg mL^−1^) as drug. Thus, inhibitors showed higher (80%) DPPH FRSA, except 149a, and 149e. 149m being highly potent, showed 83% of inhibition. 149f showed predominant inhibition (98%, IC_50_ = 11.73 ± 1.22 µg mL^−1^), due to coumarin group across thiazole, followed by 149n (IC_50_ = 38.35 ± 2.44 µg mL^−1^), bearing methyl group, that stabilizes radicals by inductive effect. 149b, and 149m (IC_50_ = 48.62 ± 2.41, 48.99 ± 2.11 µg mL^−1^) showed comparable inhibition to Ascorbic acid. Electron withdrawing ester group decreased inhibition in 149c–d (IC_50_ = 57.39 ± 2.47, 99.86 ± 0.38 µg mL^−1^). Electron withdrawing nitro group at *meta* position reduced electron density in 149j making it as the least potent (IC_50_ = 140.87 ± 1.76 µg mL^−1^), and induced moderate inhibition (IC_50_ = 68.95 ± 0.56 µg mL^−1^) in 149q (Fig. 50).^74^149f being associated with higher anti-oxidant ability, was docked against human anti-oxidant enzyme receptor 3MNG, and received binding affinity of −8.9 kcal mol^−1^. Thus, thiazole substituted coumarin developed maximum interaction with surrounding amino acid residues. Coumarin ring, and its ring containing oxygen atom displayed π-sigma, and conventional hydrogen bond with Val23 (2.88, 4.16 Å). Glu16 interacted π-anion interactions with coumarin (4.99 Å), and fluorene ring (3.48 Å), while Leu28 through electrostatic interaction (5.37 Å). Leu96 interacted fluorene ring (5.29 Å), through π–alkyl interaction, and π-cation interaction with Arg86 (4.55 Å) as well as π–alkyl (5.33 Å) interactions with Ala90 (5.00 Å) by electrostatic interaction.^74^

**Scheme 21:**
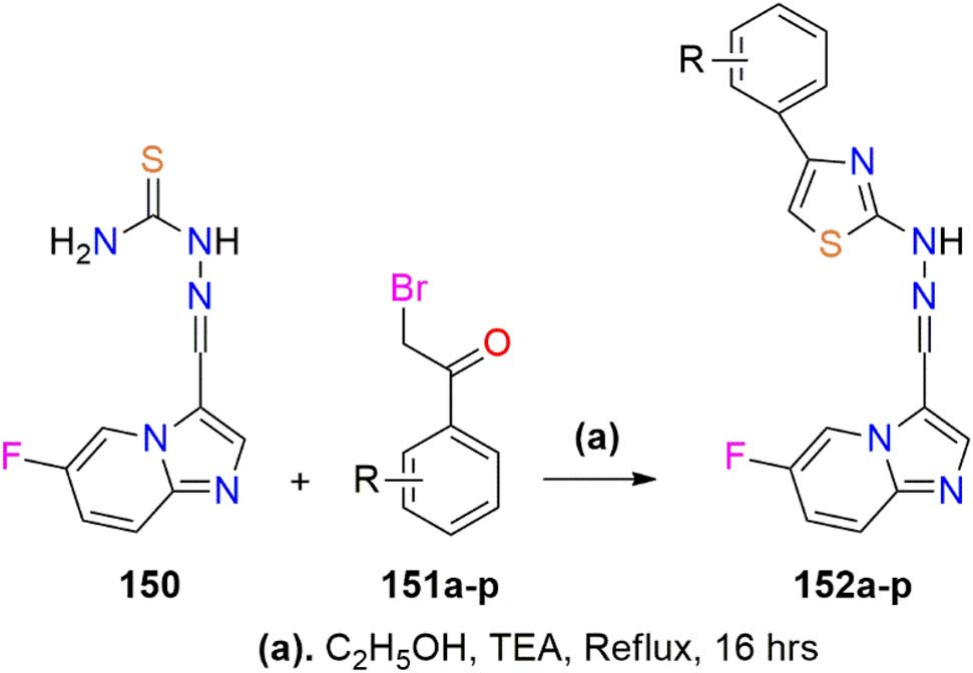
Synthesis of hydrazinyl thiazoles 152a–p.

R. Hussain *et al.* synthesized sixteen imidazopyridine-based thiazoles (Scheme 21). Thus, imidazopyridine based thiosemicarbazone compound 150, and 2-bromoacetophenones 151a–p were refluxed in ethanol, and TEA for 16 h to get hydrazinyl thiazoles 152a–p.^75^

**Fig. 51:**
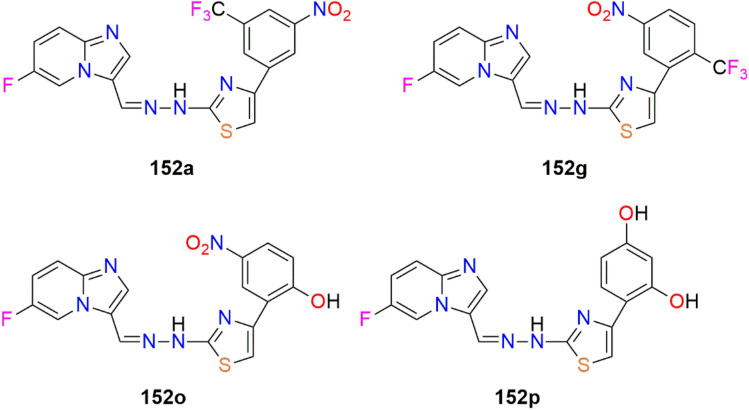
Structures of the potent imidazopyridine-derived thiazoles.

152a–p were potent α-glucosidase inhibitors (5.57 ± 3.45–63.46 ± 5.28 µM) in comparison to Acarbose (IC_50_ = 48.71 ± 2.65 µM) as drug. 152a, 152g, and 152o–p bearing electron withdrawing, and hydrogen bond donor groups induced potent inhibitions (IC_50_ = 6.85 ± 2.18, 5.57 ± 3.45, 7.16 ± 1.40, 10.48 ± 2.20 µM) than drug (Fig. 51).^75^

**Fig. 52:**
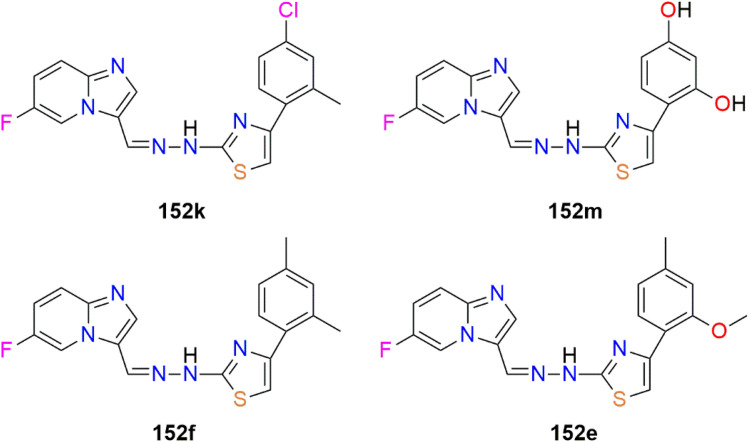
Structures of the potent imidazopyridine-derived thiazoles.

152k showed better inhibition (IC_50_ = 34.73 ± 5.23 µM) than drug due to EWGs. 152m is less potent (IC_50_ = 56.30 ± 5.94 µM) than drug, 152f is good inhibitor (IC_50_ = 50.96 ± 5.80 µM), and 152e is better inhibitor (IC_50_ = 44.89 ± 5.27 µM) than drug (Fig. 52).^75^

**Fig. 53:**
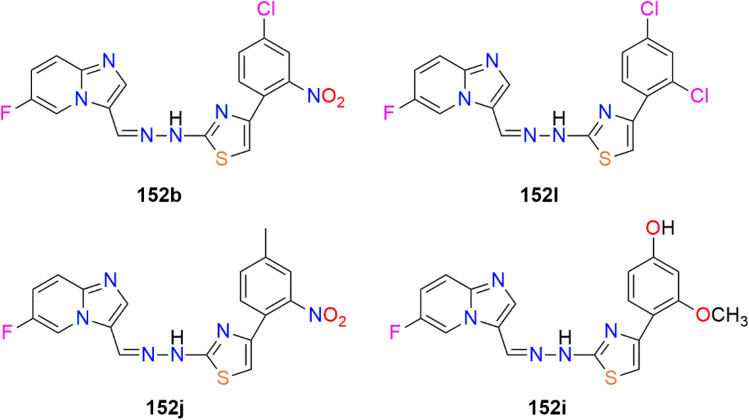
Structures of highly potent imidazopyridine-derived thiazoles.

152b (IC_50_ = 13.63*±* 1.67 µM), 152l (IC_50_ = 19.26 ± 2.58 µM), 152j (IC_50_ = 22.57 ± 3.55 µM), and 152i (IC_50_ = 34.91*±* 5.84) were many-fold potent than drug (Fig. 53).^75^

**Fig. 54:**
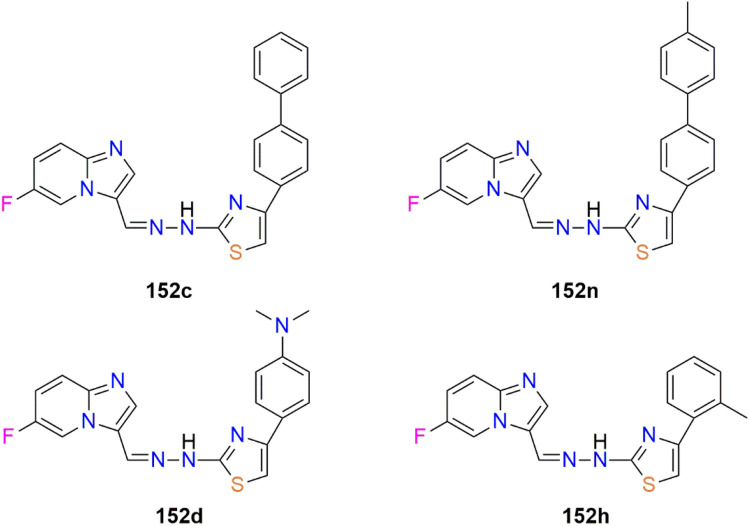
Structures of the least, and moderate imidazopyridine-derived thiazole inhibitors.

The presence of bulky groups decreased inhibition. 152c, and 152n (IC_50_ = 63.46 ± 5.28, 61.42 ± 4.56 µM) were least, 152d, and 152h (IC_50_ = 32.12 ± 4.293, 43.20 ± 6.16 µM) as moderate inhibitors (Fig. 54).^75^

MD analysis validated *in vitro* and *in silico* results. 152g, 152a, and 152o–p (−13.45, −12.87, −12.15, −11.25 kcal mol^−1^) interacted active sites of α-glucosidase, are potent (IC_50_ = 5.57 ± 3.45, 8.85 ± 2.18, 7.16 ± 1.40, 10.4–8±2.20 µM) inhibitors. MD analysis suggests 152a, and 152o as potent inhibitors, with excellent interactions against the active site. MD showed 152g being the most potent, interacted Phe200 (π–π T shaped), Glu595 (π-anion), Val96 (π–alkyl, π–π stacked), Phe51 (π-alkyl), Thr175 (carbon–hydrogen bond), His94 (fluorine), Phe95 (fluorine, π–alkyl), and Arg91 (π–alkyl). 152a interacted Trp90 (π–sulfur, π–π T shaped), Arg91 (π–π stacked), Gln202 (carbon–hydrogen bond), His94 (fluorine, π–π stacked), Val96 (π–alkyl), Val201 (π–alkyl), and Phe200 (fluorine, π–alkyl). Docking analysis suggests strong hydrogen bond donor groups enhance inhibition. 152o interacted, Phe51 (π–π T shaped), Arg91 (π–π stacked), Val96 (π–π stacked, π–alkyl), Gln202 (carbon–hydrogen bond), Glu595 (π–anion), Phe95 (carbon–hydrogen bond), and His95 (π–π T shaped). 152p interacted Arg91 (π–alkyl), Phe200 (π–π T shaped), Val96 (π–alkyl), Val201 (π–alkyl), Glu595 (π–anion), His94 (fluorine, carbon–hydrogen bond), and Phe95 (fluorine). MD analysis augmented the potent inhibitors strongly interacted, and stabilized within the protein-binding pockets. 152a, and 152o as the most potent inhibitors, showing superior binding interactions with the active site of the α-glucosidase enzyme.^75^

**Scheme 22:**
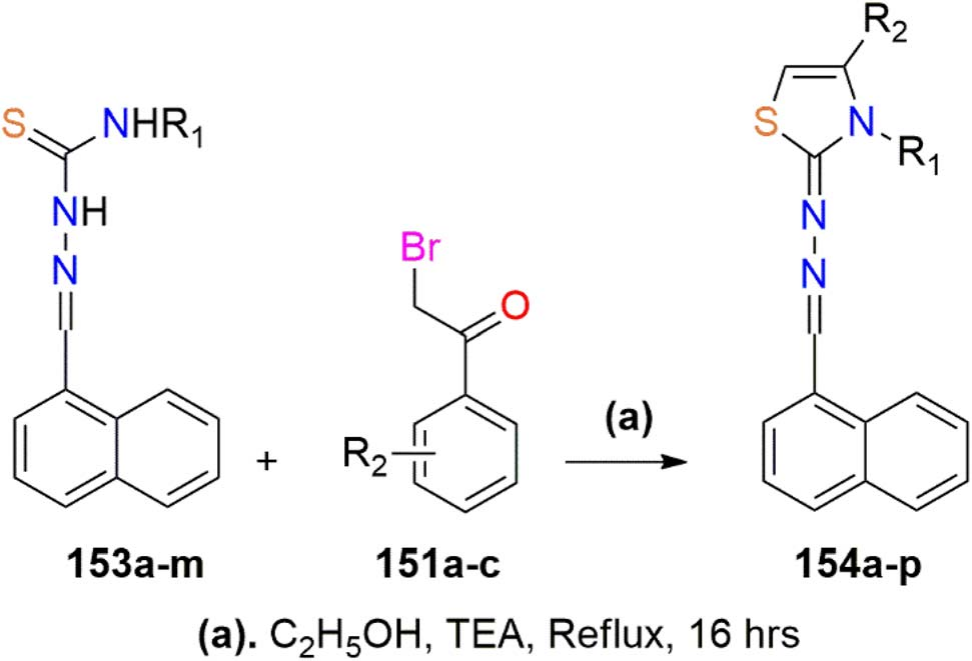
Synthesis of hydrazinyl thiazoles 154a–p.

N. F. N. Santos *et al.* synthesized eighteen naphthalene based hydrazinyl thiazoles (Scheme 22). Thus, thiosemicarbazone compounds 153a–m, were refluxed with phenacyl bromides 151a–c in ethanol, and acetic acid as catalyst for 2 h. The precipitates were washed with ethanol, and dried to get hydrazinyl thiazoles 154a–p.^76^

**Fig. 55:**
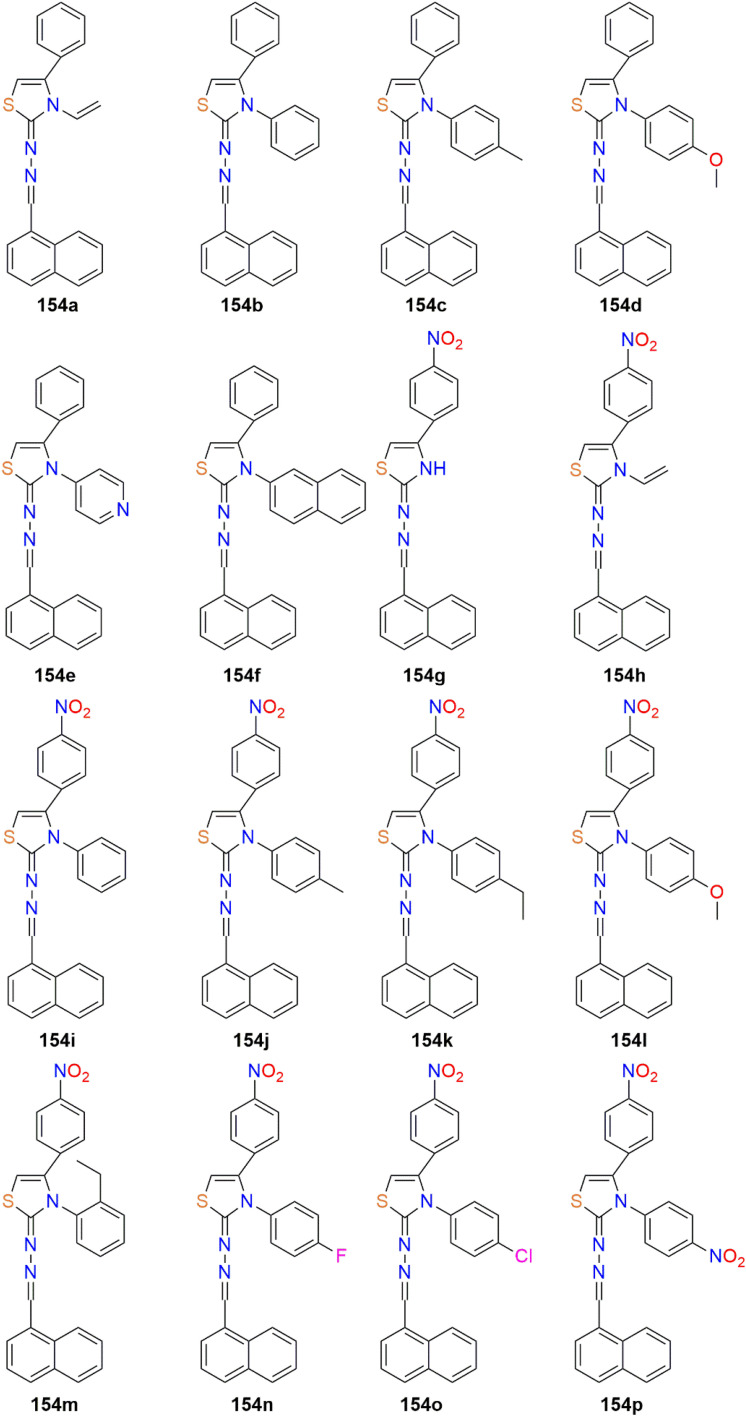
Structures of the synthesized hydrazinyl thiazoles.

154a–p were assessed *in vitro* ABTS assay (IC_50_ = 37.6->1830.3 µM). Inhibitors were classified as strong (IC_50_ < 80 µM), moderate (80–200 µM), and weak (>200 µM) anti-oxidants. Thus, 154g, and 154p received notable anti-oxidant effects (IC_50_ = 37.6 ± 0.7, 285.7 ± 2.0 µM) among the screened hybrids. More importantly, inhibitory potential enhanced with 4-nitro, 4-methoxy, and allyl groups, suggesting, structural components of the inhibitors as crucial for anti-oxidant capacity. Rest of the screened hybrids were very weak anti-oxidants, and received inhibitions well-above 1000 µM (Fig. 55).^76^

**Scheme 23:**
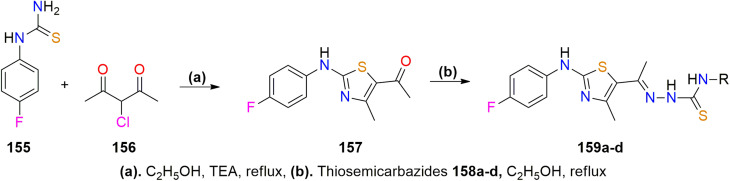
Synthesis of thiosemicarbazone based thiazoles 159a–d.

E. A. Fayed *et al.* synthesized four thiosemicarbazone-thiazole hybrids 15a–d (Scheme 23). Thus, thiourea 155, and chloro acetylacetone 156 were refluxed in absolute ethanol, using TEA as catalyst. Upon completion, the keto-thiazole 157, was then refluxed with thiosemicarbazides 158a–d, in ethanol at reflux to yield thiosemicarbazone-thiazole hybrids 159a–d.^77^

**Fig. 56:**
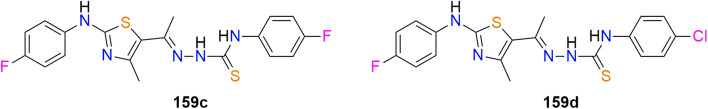
Structures of the potent hydrazinyl thiazoles.

Inhibitors 159c–d were screened against α-amylase using Acarbose (IC_50_ = 0.108 ± 0.005 µg mL^−1^) as drug. 159c–d exhibited poor α-amylase inhibition (IC_50_ = 11.78 ± 0.58, 24.18 ± 1.2 µg mL^−1^). 159c showed most potent α-glucosidase inhibition (IC_50_ = 0.446 ± 0.01 mM mL^−1^), and 159d showed good inhibition (IC_50_ = 1.914 ± 0.04 mM mL^−1^) compared to drug (IC_50_ = 0.74 ± 0.15 mM mL^−1^). Hypoglycemic 159c–d were screened against PPAR*-*γ binding, using Pioglitazone as drug (IC_50_ = 1.912 ± 0.11 ng mL^−1^). 159c–d displayed high affinity (IC_50_ = 1.594 ± 0.028, 1.631 ± 0.023 ng mL^−1^) towards PPAR*-*γ than Pioglitazone. MD analysis revealed Acarbose interacted α-amylase through twelve hydrogen bond donors (Glu240, His201, Glu233, Asp300, Asp197, Trp59, Thr163), six hydrogen bond acceptors (Lys200, Arg195, His299, His305, Gln63), and three hydrogen-arene interactions (Tyr151, Trp59). Acarbose interacts with α-glucosidase through eight hydrogen bond donors (Asp282, Asp616, Asp404, Met519), two hydrogen bond acceptors (Arg600, His674), and hydrogen-arene interaction (Phe649). Pioglitazone interacts with PPAR-γ through two hydrogen bond donors (Met364, Met348), four hydrogen bond acceptors (Ser289, His323, Tyr473, His449), and single arene–hydrogen interaction (Cys285). Against α-amylase, 159c exhibited a total of four interactions, comprising hydrogen bond, and three arene-hydrogen contacts. 159d demonstrated five stabilizing interactions within the active site, comprising three hydrogen bonds, and two arene-hydrogen contacts. The binding energy values (−10.23, −10.85 kcal mol^−1^) were more favorable than Acarbose (−9.547 kcal mol^−1^). Against α-glucosidase, 159c–d received scores of −11.01, and −10.47 kcal mol^−1^ in comparison to (−9.54 kcal mol^−1^). 159c showed three hydrogen bonds (Asp616, Met519, Trp613), 159d showed three hydrogen bonds (Asp616, Met519, Trp613). Against PPAR-γ, 159c–d showed scores (−12.42, −12.61 kcal mol^−1^) close to Pioglitazone (−12.77 kcal mol^−1^). 159c developed three hydrogen bonds (Cys285), and arene–hydrogen interaction (Gly284). 159d showed three hydrogen bonds (Ser289, Cys285, Ser342). MD analysis showed alignment with *in vitro* antihyperglycemic evaluation, and suggest 159c–d as promising antidiabetic hybrids (Fig. 56).^77^

**Scheme 24:**
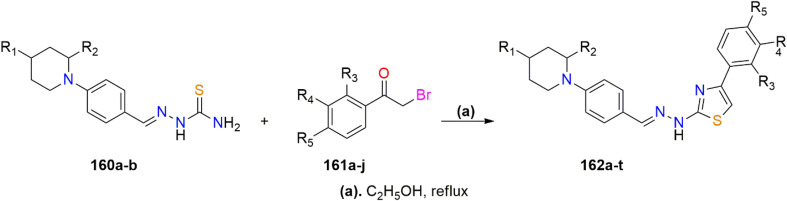
Synthesis of piperidine-based hydrazinyl thiazoles 162a–t.

U. Ghani *et al.* synthesized twenty piperidine based hydrazinyl thiazole hybrids 162a–t(Scheme 24). Thus, thiosemicarbazone compounds 160a–b, and phenacyl bromides 161a–j were refluxed in absolute ethanol for 3 h. Upon completion, the mixture was chilled, filtered, and crystallized from ethanol 162a–t.^78^

**Fig. 57:**
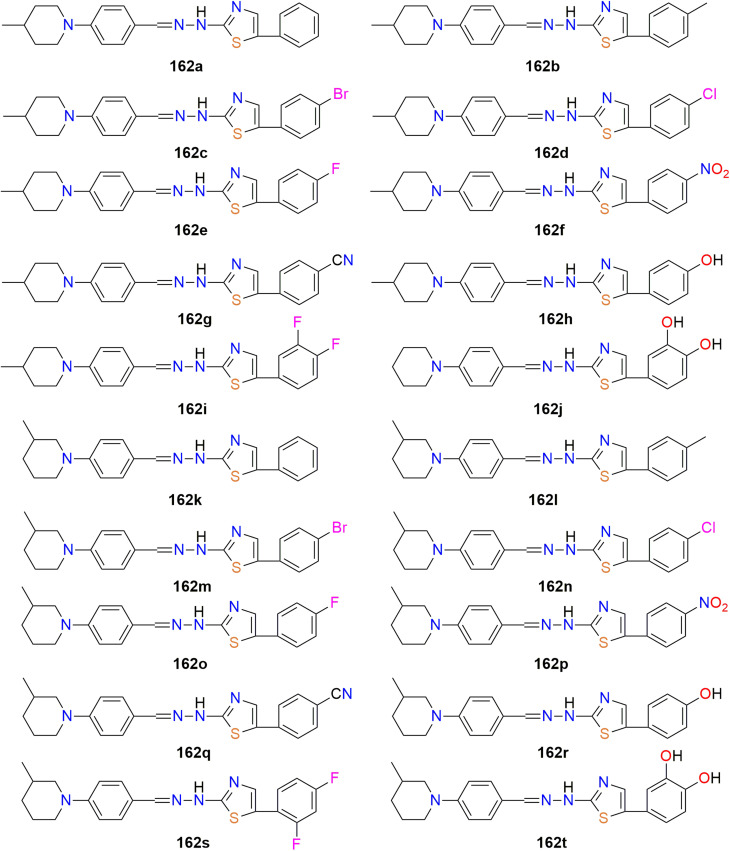
Structures of 4-methylpiperidine 162a–j, and 3-methylpiperidine 162k–t isomers.

In α-glucosidase assay, 162a–t displayed dose-dependent inhibitions compared to Acarbose (*K*_i_ = 4.81 ± 0.35 µM, IC_50_ = 10.65 ± 0.42 µM). 162a–j were more potent (*K*_i_ = 0.65 ± 0.01–37.33 ± 1.20 µM, IC_50_ = 0.61 ± 0.02–38.08 ± 1.47 µM) inhibitors than 162k–t(*K*_i_ = 11.17 ± 0.34–41.02 ± 1.30 µM, IC_50_ = 12.07 ± 0.29–40.40 ± 1.28 µM). 162a–t showed non-competitive inhibition except 162g, as competitive inhibitor. 162a–j showed enhanced α-glucosidase inhibition than 162k–t, some members were comparable inhibitors. 162e served as the most potent inhibitor among 162a–j (*K*_i_ = 0.65, IC_50_ = 0.61 µM) followed by 162h (*K*_i_ = 4.96 ± 0.14, IC_50_ = 5.73 ± 0.16 µM), and 162b (*K*_i_ = 5.68 ± 0.17, IC_50_ = 6.56 ± 0.22 µM).^78^ Among 162k–t, the most potent inhibitor was 162r (*K*_i_ = 11.17 µM, IC_50_ = 12.07 µM) followed by 162t (*K*_i_ = 18.10 ± 0.52, IC_50_ = 18.47 ± 0.74 µM), 162p (*K*_i_ = 16.91 ± 0.47, IC_50_ = 17.77 ± 0.33 µM), 162q (*K*_i_ = 17.18 ± 0.52, IC_50_ = 16.92 ± 0.81 µM), and 162o (*K*_i_ = 18.03 ± 0.48, IC_50_ = 20.11 ± 0.76 µM). Thus, hydroxyl, dihydroxyl, nitro, cyanide, and fluorine groups on phenylthiazole, and unsubstituted 162k (*K*_i_ = 14.83 ± 0.38, IC_50_ = 15.41 ± 0.33 µM) were more potent, augmenting positional isomerism significantly influenced inhibitions.^78^ The presence of 4-methyl piperidine ring in 162a–j, enhanced enzyme inhibition compared to 3-methyl group in 162k–t. Notable differences occurred among inhibitions of 162e (*K*i = 0.65, IC_50_ = 0.61 µM), and 162o (*K*i = 18.03, IC_50_ = 20.11 µM) due to positional isomerism. Similarly, differences in inhibitions were observed among other isomers such as; 162a–k, 162g–q, and 162i–s, where isomeric configuration contributed very little to potencies, highly aligned with computational analysis (Fig. 57).^78^

In molecular dynamics, non-competitive inhibitors bind allosteric site residues Val265, Lys15, Lys262, Ile271, His258, Ala289, Ser295, Thr273, Glu293, Val294, Asp338, Trp14, and Ser339 of the homology model of yeast α-glucosidase. 162a (*K*_i_ = 17.56 µM, IC_50_ = 16.32 µM) interacted through electrostatic, and hydrophobic contacts. Hydrazine linker formed weak hydrogen bond with Val294. Lys262, Ala289, and Asp338 engaged in hydrophobic interactions through 4-methylpiperidine, and aryl rings. Benzylidene ring developed π–cation interaction (His258). In 162b (*K*_i_ = 5.68 µM, IC_50_ = 6.56 µM), strong electrostatic, and hydrophobic interactions occurred. Hydrogen bonding interactions were established through nitrogen atoms of the linker (His258, Thr273, Glu293), and thiazole ring formed four hydrogen bond with Ser295. Toluene, and 4-methylpiperidine developed hydrophobic interactions (Ile271, Ala289) in 162b. Toluene moiety facilitated T-shaped π–π interaction (Trp14). 162b, and 162l showed similar patterns. 162l (*K*_i_ = 23.00 µM, IC_50_ = 23.86 µM), through the linker nitrogen atoms, engaged in hydrogen bonds (His258, Thr273), and three hydrogen bonds occurred between thiazole, and Ser295 with allosteric site. Hydrophobic stabilization was mediated by 3-methylpiperidine, benzylidene, and toluene groups with Val265, Ile271, and Ala289, complemented by T-shaped π–π stacking between the toluene, and Trp14. Allosteric stabilization of 162e (*K*_i_ = 0.65 µM, IC_50_ = 0.61 µM) was achieved through hydrophobic interactions (Ile271, Ala289), complemented by hydrogen bonding (His258, Thr273, Glu293, Ser295), and T-shaped π–π stacking arrangement mediated between fluoro-benzene ring, and Trp14. In 162o (*K*_i_ = 18.03 µM, IC_50_ = 20.11 µM), linker region formed one hydrogen bond (Val294), and stabilization achieved through hydrophobic interactions (Asp338, Ile271, Ala289).^78^

162o lacks T-shaped stacking of fluorobenzene (Trp14), making 162e as more potent than 162o. 162q (*K*_i_ = 17.18 µM, IC_50_ = 16.92 µM) achieves stabilization within allosteric site through hydrogen bonding interactions involving nitrile group's nitrogen atom, linker region, and thiazole ring (Lys15, His258, Ser295). Hydrophobic interactions involving Trp14, Lys262, Ile271, and Ala289, mediated by the 3-methylpiperidine and benzonitrile groups, enhanced stabilization within the binding pocket. 162q conformation in allosteric site was consistent with that of non-competitive inhibitors. 162g (*K*_i_ = 17.82 µM, IC_50_ = 36.87 µM) adopted U-shaped orientation in the active site, where the linker nitrogen atom formed a hydrogen bond with Phe157 and the nitrile group established a hydrogen bond with Asn347. These interactions were further reinforced by hydrophobic contacts with Phe300 and Asn349.^78^

### 2.2. Azomethine-thiazole hybrids

Azomethine-thiazole hybrids are privileged heterocyclic scaffolds due to synergistic integration of the biologically versatile thiazole ring with azomethine linkage. This combination generates conformationally flexible molecular framework capable of engaging multiple biological targets implicated in DM. The azomethine functionality enhances electronic delocalization, and binding adaptability, while the thiazole core contributes metabolic stability, and broad bioactivity. Collectively, these features enable effective inhibition of key carbohydrate-digesting enzymes, including α-glucosidase, and α-amylase, comparable to Acarbose, thereby attenuating postprandial hyperglycemia. Beyond glycemic control, several azomethine-thiazole hybrids modulate the polyol pathway through aldose reductase inhibition, offering protection against chronic diabetic complications. Importantly, these hybrids frequently exhibit pronounced anti-oxidant activity, to scavenge free radicals efficiently, and mitigate oxidative stress-induced damage to pancreatic β-cells. Because of the central role of oxidative stress in diabetes progression, the combined enzyme inhibitory, and anti-oxidant properties turn azomethine–thiazole hybrids as promising multifunctional candidates for comprehensive antidiabetic therapy.

**Scheme 25:**
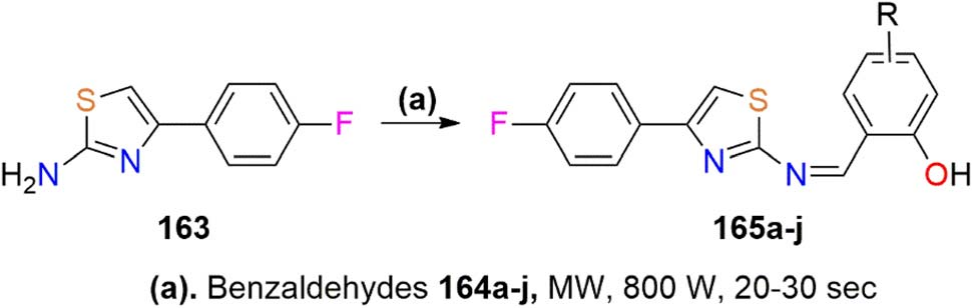
Synthesis of azomethine-thiazole hybrids 165a–j.

D. Bhosale *et al.* synthesized ten azomethine-thiazole hybrids 165a–j (Scheme 25). Thus, thiaole-2-amine 163, and benzaldehydes 164a–j were mixed in absolute ethanol, using piperidine as catalyst, and microwave irradiated (800 watt power) for 20–30 seconds.^79^

165a–j were subjected to *in vitro* anti-inflammatory assay using Ibuprofen as drug, to assess their ability to inhibit heat-induced protein denaturation. In terms of % inhibition employing serial dilutions (100–1000 µg mL^−1^), 165a–j showed dose-dependent inhibition (6.25–77.77%). 165a exhibited highest inhibition (77.77%), close to Ibuprofen (80.55%) at 1000 µg mL^−1^. With decrease in concentration to 100 µg mL^−1^, 163a, and 163f showed excellent % inhibition (63.19, 59.72%) compared to Ibuprofen (42.36%). 163b (31.25%) 163c (1.48%), 163e (8.33%), 163h (0.69%), and 163i (8.32%), showed very low % inhibition than Ibuprofen, with 163d, and 163g devoid of any significant anti-inflammatory effects at 100 µg mL^−1^. To conclude, inhibitors bearing electron withdrawing groups at 3, 4, 5 positions possess potent anti-inflammatory effects than electron withdrawing groups, and unsubstituted inhibitors. In terms of IC_50_ values, 163h showed comparable inhibition (IC_50_ = 399.8 µg mL^−1^) to Ibuprofen (IC_50_ = 348.5 µg mL^−1^).^79^

**Fig. 58:**
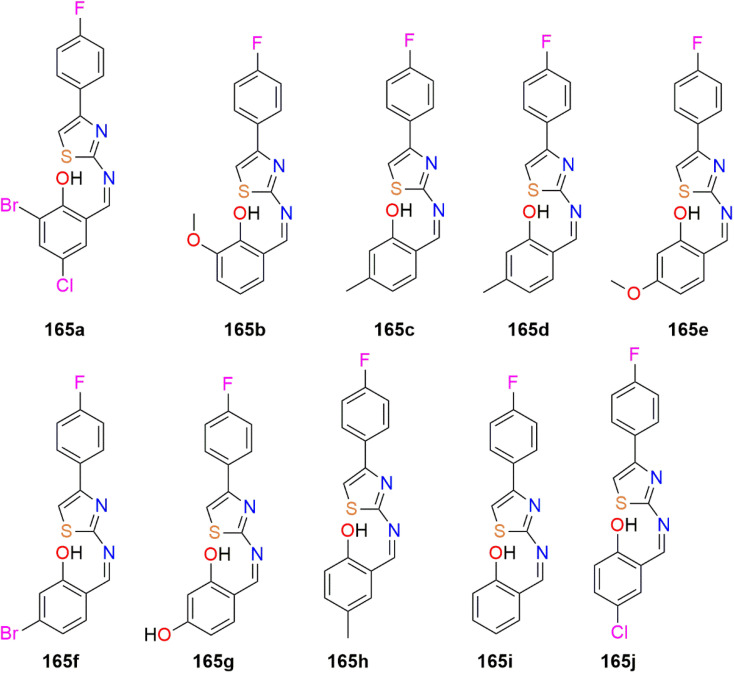
Structures of the synthesized azomethine-thiazole hybrids.

165a–j were screened for % DPPH FRSA assay using Ascorbic acid as drug. 165a–j received % inhibition (6.18–25.89%), and IC_50_ values (36.10–957.66 µg mL^−1^). Modest % inhibition was observed compared to Ascorbic acid. In terms of IC_50_ values, 165e–j exhibited lower IC_50_ values, than 165a–b, and 165d compared to drug. 165j, being the most potent, received lowest IC_50_ value (436.10 µg mL^−1^). Anti-oxidant potential decreases as: 165j > 165g > 165h > 165i > 165f > 165e compared to drug (Fig. 58).^79^

**Scheme 26:**
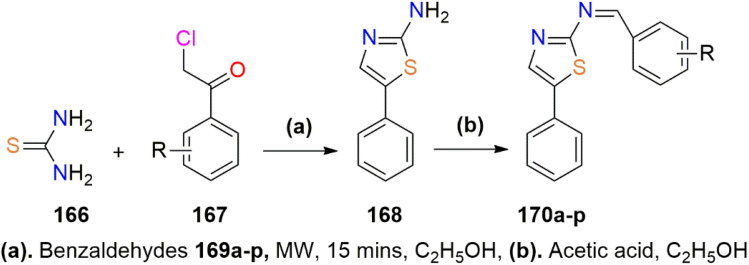
Synthesis of azomethine-thiazoles 170a–p.

H. Ullah *et al.* synthesized sixteen azomethine-thiazole hybrids 169a–p. Thus, phenacyl bromide 167 reacted thiourea 166 in ethanol under irradiation for 15 min to afford intermediate 168, which upon reflux with benzaldehydes 169a–p in ethanol, using acetic acid as catalyst, for 3–4 h yield azomethine-thiazole hybrids 170a–p(Scheme 26).^80^

In α-amylase assay, 170a–p showed variable effects (0.6 ± 0.05–32.20 ± 0.50 µM) compared to Acarbose (IC_50_ = 8.90 ± 0.10 µM). 170f, 170e, 170k, 170d, 170o, 170a, and 170b (IC_50_ = 0.6 ± 0.05, 0.80 ± 0.05, 0.90 ± 0.05, 2.30 ± 0.10, 4.10 ± 0.05 7.90 ± 0.10, 11.70 ± 0.20, µM) served as better inhibitors. SAR suggests 170f emerged as most potent inhibitor, due to *ortho*-OH group, involved in hydrogen bonding with active site residues. Electron withdrawing chloro group in 170f activates ring, and is more potent than 170o because of differently substituted phenyl rings. Similarly, difference in inhibitory effects of 170a, 170b, and 170h (IC_50_ = 13.40 ± 0.20 µM), arise due to NO_2_ group located at *ortho, para,* and *meta* positions across phenyl ring. In 170d (*ortho*, *para*), and 170g (*meta*, *para*) with chlorine atoms attached, 170d was more potent than 170g (IC_50_ = 2.30 ± 0.10, 8.60 ± 0.30 µM). 170e is the 2^nd^ most potent inhibitor (IC_50_ = 0.80 ± 0.05 µM), owing to hydrogen bonding mediated by *para*-OH group, with the active site residues. 170e (*para*-OH*, meta*-OCH_3_), and 170k (*meta*-OH*, para*-OCH_3_) slightly differ in their inhibitions (IC_50_ = 0.90 ± 0.05 µM), owing to the different locations of EDGs (OH, OCH_3_) across phenyl ring B. Convenient hydrogen bonding of *para*-hydroxyl group occurs with the active site residues. In 170i–j, bulky groups (benzyloxy/anthracenyl) decreased inhibitions (IC_50_ = 32.20 ± 0.50 µM).^80^ In α-glucosidase assay, variable inhibitions were obtained (IC_50_ = 0.50 ± 0.05–32.70 ± 0.50 µM) compared to Acarbose (IC_50_ = 9.10 ± 0.10). 170a, 170c–g, 170i, and 170o (IC_50_ = 7.90 ± 0.10, 5.80 ± 0.10, 3.50 ± 0.10, 1.20 ± 0.05, 0.50 ± 0.05, 6.45 ± 0.30, 0.80 ± 0.05, 3.20 ± 0.05 µM) emerged as excellent inhibitors. SAR suggest, nature, number, and position of groups effects inhibition.^80^

**Fig. 59:**
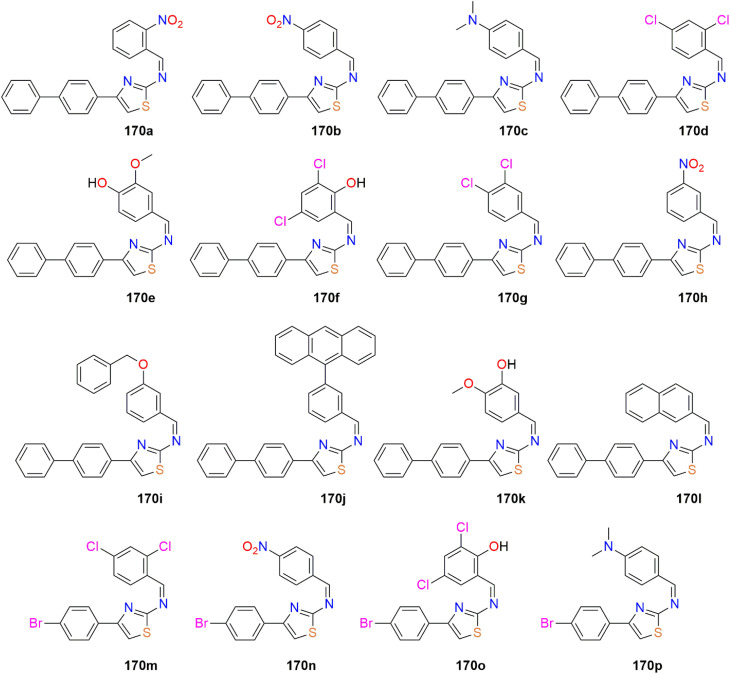
Structures of the potent azomethine-thiazole hybrids.

• Differences in inhibitory effects of 170f (IC_50_ = 0.50 ± 0.05 µM), and 170o (IC_50_ = 3.20 ± 0.05 µM), reflect difference in substitution patterns across phenyl rings.

• 170a, 170b, and 170h with variable inhibitions (IC_50_ = 7.90 ± 0.10, 10.80 ± 0.20, 12.80 ± 0.20 µM) possess nitro group at *ortho, meta,* and *para* positions across phenyl rings.

• 170d (IC_50_ = 3.50 ± 0.10 µM), and 170g (IC_50_ = 6.45 ± 0.30 µM) contain chlorines at different positions.

• 170e, and 170k possess EDGs (hydroxyl, methoxy), showed excellent inhibitions.

• Inhibitions decreased with bulky groups in 170i–j (Fig. 59).^80^

Molecular docking analysed binding modes of the inhibitors against α-amylase, and α-glucosidase enzymes. Enhanced inhibitions were shown by inhibitors bearing both EDGs, and EWGs. In α-amylase assay, and among the most potent inhibitors 170f, 170e, and 170k, 1^st^ inhibitor showed hydrogen bond (Arg346), and hydrophobic contacts (π-stacking). 170f with elevated potency, suggests synergistic contribution of OH group (activation), and Cl group (deactivation), with activating influence exerts stronger effect than deactivating one. Similarly, the ranked 2^nd^ inhibitor 170e interacted with Asp433. 170f showed pronounced α-glucosidase inhibitory potential, extra interaction with Asn241, and Phe157. 2^nd^ inhibitor 170k revealed interaction with His245, and Phe157. To conclude, against α-amylase enzyme, mostly single interaction with different energy, was observed. Against α-glucosidase enzyme, numerous key interactions were observed, facilitated by the enzyme's active site embedded in the protein framework.^80^

### 2.3. Pyrazole clubbed thiazoles

Pyrazole-thiazole hybrids exert antidiabetic effects through multi-target therapeutic strategy that simultaneously regulates glucose metabolism, and underlying metabolic dysfunctions. These hybrids primarily inhibit key carbohydrate-hydrolyzing enzymes, thereby reducing intestinal glucose absorption, and effectively controlling postprandial hyperglycemia. Several analogues exhibit potent inhibitory activity against α-amylase, and α-glucosidase, highlighting their strong enzyme-binding efficiency. Beyond enzyme inhibition, pyrazole-thiazole hybrids are frequently designed as structural, and functional analogs of thiazolidinediones, such as Pioglitazone, enabling them to activate the nuclear receptor PPAR-γ, enhance insulin sensitivity, and promote glucose uptake in peripheral tissues. Recent studies have further demonstrated their ability to inhibit DPP-IV, an enzyme responsible for incretin hormone degradation, thereby prolonging insulin secretion, and improving glycemic control. Collectively, the dual modulation of digestive enzymes, and insulin-sensitizing pathways positions pyrazole-thiazole hybrids as promising multifunctional scaffolds for the development of next-generation antidiabetic agents.

**Scheme 27:**
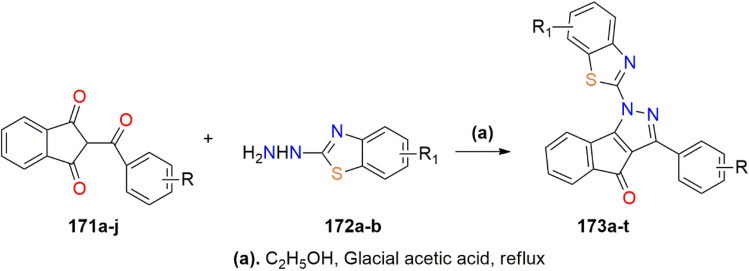
Synthesis of benzothiazole appended fused indenopyrazolones 173a–t.

R. Punia *et al.* synthesized twenty indeno-pyrazole-based thiazoles 173a–t. Thus, equimolar mixture of indene-diones 171a–j, and 2-hydrazinylthiazoles 172a–b were refluxed in ethanol, and glacial acetic acid as catalyst (Scheme 27).^81^

**Fig. 60:**
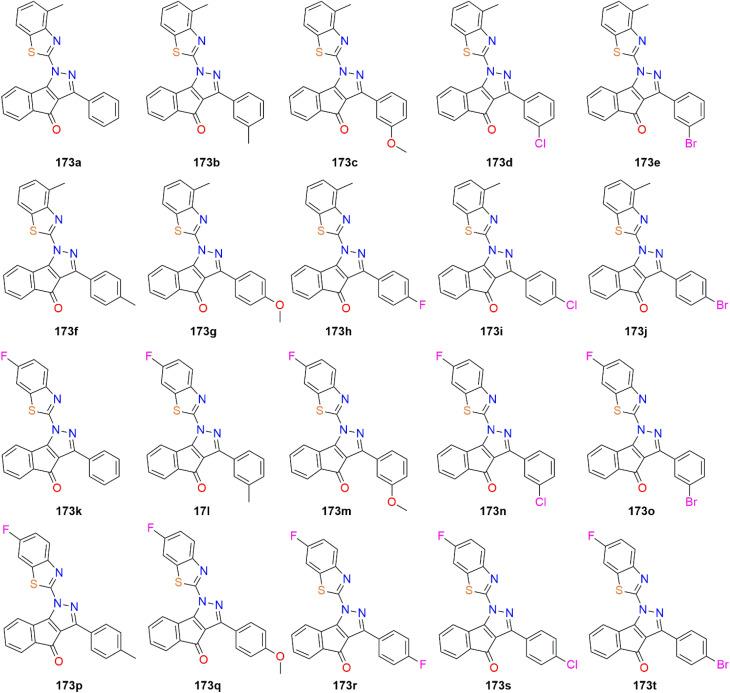
Structures of the screened indeno-pyrazole-based thiazoles 173a–t.

173(a–t) were subjected to in *in vitro* α-amylase assay using Acarbose as drug. Thus, 173i, 173k–l, and 173q served as better inhibitors (IC_50_ = 92.99 ± 1.94, 98.42 ± 2.19, 95.41 ± 3.92, 104.74 ± 3.26 µg mL^−1^). 173j, 173p, and 173r (IC_50_ = 132.41 ± 7.02, 135.14 ± 3.22, 144.10 ± 5.34 µg mL^−1^) exhibited good α-amylase inhibitions. 173a–h, 173m–o, and 173s–t (IC_50_ = 159.95 ± 5.66–200.95 ± 26.23 µg mL^−1^) showed moderate inhibitions than Acarbose (IC_50_ = 103.60 ± 2.15 µg mL^−1^) (Fig. 60).^81^

**Fig. 61:**
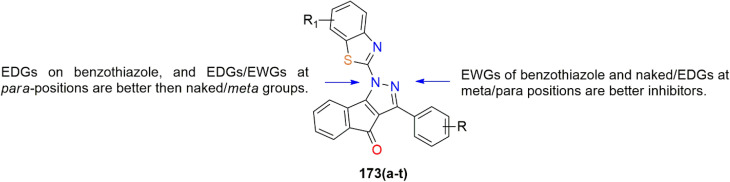
SARs of the screened indeno-pyrazole-based thiazoles 173a–t.

Structure activity relationship analysis suggests:

• Indenopyrazolone-linked inhibitors featuring benzothiazole (R_1_ = 4-CH_3_), and phenyl rings (R = 4-Cl), as well as benzothiazole (R_1_ = 6 F), and aryl groups (R = H, 3-CH_3_, 4-OCH_3_), exhibit superior inhibition.

• Inhibitors bearing 4-CH_3_ on benzothiazole, along substituted aryls appended with indenopyrazolones, showed discernible inhibitions as R = 4-Cl > 4-Br > 4 F > 4-OCH3 > 4-CH_3_ > H > 3-Cl > 3-Br > 3-CH_3_ > 3-OCH3. Inhibitors with R_1_ = 6-F on benzothiazole, coupled with substituted phenyl ring inhibited: R = 3-CH_3_ > H > 4-OCH_3_ > 4-CH_3_ > 4-F > 3-OCH_3_ > 4-Cl > 3-Cl > 3-Br > 4-Br (Fig. 61).^81^

173i, and 173l being most potent, were docked into the active site of α-amylase. Inhibitor–receptor complexes were stabilized through indanone, and electrostatic π–anion interactions (ASP329). Benzothiazole, and TRP12 formed T-shaped π–π contacts, while similar interaction occurred between TYR55, and indanone. Pyrazole, and SER335 established π-donor type hydrogen bonding. Acarbose acted as hydrogen donor (ASN53, ASP329, HIS236, SER51, GLU262), and hydrogen acceptor (LYS235). Binding energies of 173l, and 173i (−8.0, −8.2 kcal mol^−1^) were higher than Acarbose (−6.9 kcal mol^−1^).^81^

**Scheme 28:**
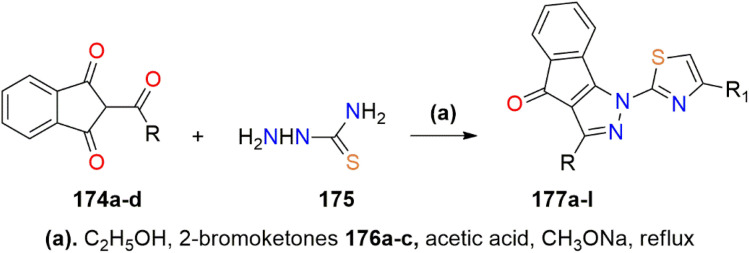
Synthesis of indeno-pyrazole based thiazoles 177a–l.

S. Mor and M. Khatri synthesized twelve indeno-pyrazole based thiazoles 177a–l(Scheme 28). Thus, indene-1,3-diones 174a–d, and thiosemicarbazide 175 were refluxed in methanol, adding sodium acetate, α-bromoketones 176a–c, and glacial acetic acid as catalyst for 5–8 h to yield indeno-pyrazole based thiazoles 177a–l.^82^

**Fig. 62:**
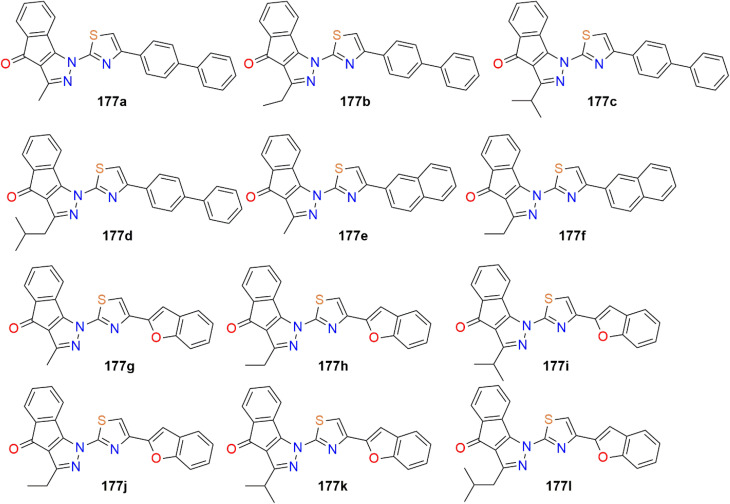
Structures of indeno-pyrazole based thiazoles 177a–l.

177a–l were assessed for their antidiabetic effects, using Acarbose as standard, in α-amylase inhibition assay. Thus, 177j–k (IC_50_ = 0.79, 0.46 µM) served as the most potent inhibitors, followed by 177i (IC_50_ = 0.94 µM), and 177l (IC_50_ = 0.89 µM) as good inhibitors. Similarly, 177a (IC_50_ = 5.29 µM), 177e–f (IC_50_ = 6.88, 4.17 µM), and 177h (IC_50_ = 3.21 µM) were found to be moderately potent inhibitors. 177b–d, and 177g (IC_50_ = 10.05,20.51, 10.78, 18.31 µM) showed poor inhibition than Acarbose (IC_50_ = 0.11 µM).^82^ SARs analysis suggests:

• Benzofuran-2-yl on thiazole are potent than 2-naphthyl, and 1,1-biphenyl.

• Indenopyrazoles bearing iso-propyl on pyrazole, and 2-naphthyl/1,1-biphenyl on thiazole ring are least potent than methyl, ethyl, and iso-butyl groups on pyrazole.

• I-propyl group on pyrazole, and benzofuran-2-yl on thiazole ring are the most potent inhibitors (Fig. 62).^82^

177j–k, being the most potent were docked against α-amylase (PDB id: 7TAA). Benzofuran ring's oxygen atom hydrogen bonded with NH of Arg344, pyrazole ring's nitrogen atom interacted Gln35 through hydrogen bond. Indenopyrazole developed π–π stacked interactions with Tyr75, and benzofuran moiety with Tyr82. Thiazole ring created π-anion interaction with Asp340 in 177j–k, and π-cation with Arg344 in 177k. 177j–k, and Acarbose received affinity of −8.7, −9.0, and −10.1 kcal mol^−1^ (IC_50_ = 0.79, 0.46, 0.11 µM). Thus, the closely related binding affinity, and IC_50_ values suggests good correlation between experimental, and theoretical results.^82^

### 2.4. Thiazolidinones

Thiazolidinones represent a well-established class of heterocycles with considerable importance in antidiabetic drug discovery, largely due to their close pharmacophoric resemblance to clinically used thiazolidinediones. Thiazolidin-4-one scaffold enables effective interaction with molecular targets governing glucose, and lipid homeostasis, positioning these compounds as versatile templates for designing next-generation antidiabetic agents with improved therapeutic profiles. Their antidiabetic activity is primarily mediated through PPAR-γ activation, leading to enhanced insulin sensitivity and improved peripheral glucose utilization, along with inhibition of key carbohydrate-hydrolyzing enzymes such as α-glucosidase, thereby attenuating postprandial hyperglycemia. Beyond glycemic control, numerous thiazolidinone derivatives exhibit notable anti-oxidant properties, which are particularly advantageous in counteracting oxidative stress, and chronic low-grade inflammation associated with diabetes, and its long-term complications. Collectively, the multifunctional biological profile of thiazolidinones underscores their continued relevance as promising scaffolds in the development of safer, and more effective antidiabetic therapeutics.

**Scheme 29:**
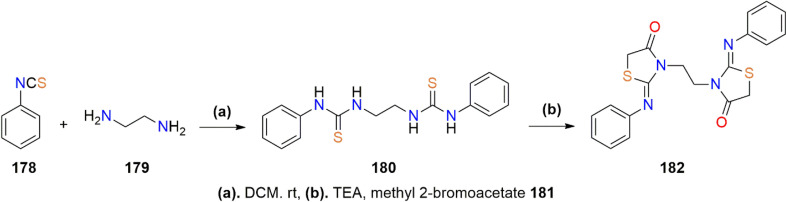
Synthesis of 3,3-ethane-1,2-diylbis(2-benzylidene-1,3-thiazolidin-4-one 182.

L. H. Abdel-Rahman, S. K. Mohamed, Y. El Bakri *et al.* synthesized 3,3-ethane-1,2-diylbis(2-benzylidene-1,3-thiazolidin-4-one) 182. Thus, phenylisothiocyanate 178 reacted diethylamine 179 in DCM at ambient temperature to prepare *N*,*N*″-ethane-1,2-diylbis[3-phenyl(thiourea)] 180. Later, 180, and ethyl bromoacetate 181 were refluxed in ethanol, using TEA as catalyst, for 5 h to yield 182. The resulting solid was filtrated, and recrystallized from ethanol (Scheme 29).^83^

Molecular docking analysis suggested 182 received higher binding affinity (−7.3 kcal mol^−1^). 182 interacted, His232, Asp329, Phe188, Arg376, Glu256, His90, Asp372, Ala229, Leu196, Asp228, and His23 of the active site. The observed interactions include carbon hydrogen bonding, π–sulfur, π–cation, π–anion, hydrogen bonding, π–sigma, and π–alkyl. Tolbutamide, Acarbose, and Rimonabant (−7.1, −7.9, −7.8 kcal mol^−1^) as drugs were docked to the active site. Thus, Rimonabant, and Acarbose received higher, and Tolbutamide lower energy. Drugs interacted similar surrounding residues of the active pocket, mainly through hydrophobic interactions.^83^

**Scheme 30:**
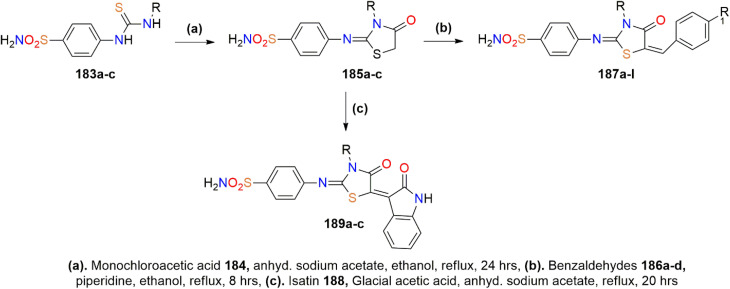
Synthesis of benzenesulfonamide-thiazolidinone hybrids 187a–l, and 189a–c.

M. A. Gamal *et al.* synthesized fifteen sulfonamides based thiazolidinones 187a–l, and 189a–c(Scheme 30). Thus, monochloroacetic acid 184 cyclized thioureas 183a–c in anhydrous sodium acetate as catalyst, using ethanol at reflux to get 1,3-thiazolidin-4-ones 185a–c. Later, thiazolidinone 185a–c, and benzaldehydes 186a–d were condensed in piperidine as catalyst to yield arylidenes 187a–l. Similarly, condensation of thiazolidinones 185a–c, and isatin 188 using glacial acetic, and anhydrous sodium acetate as catalyst yield isatins 189a–c.^84^

**Fig. 63:**
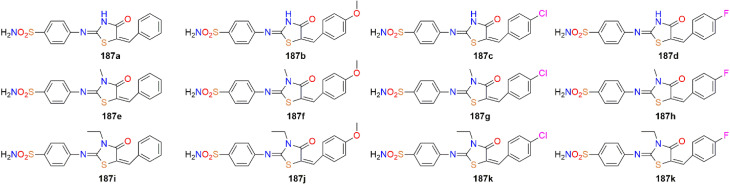
Structures of sulfonamide-based thiazolidinones 187a–k.

187a–l, and 189a–c were assessed against α-glucosidase using Acarbose as drug. Thus, good to excellent inhibitions were observed (IC_50_ = 0.3456–3.346 µM) compared to drug (IC_50_ = 0.420 µM). 187b, 187d–e, and 187k served as the most potent inhibitors (IC_50_ = 0.475, 0.440, 0.3456, 0.447 µM). 187e showed highest inhibition (IC_50_ = 0.3456 µM). Unsubstituted thiazolidinones 187a–d showed enhanced inhibitions, compared to *N*-methyl or *N*-ethyl groups in 187e–l where inhibitions decreased. Relative to unsubstituted thiazolidinone 187a (IC_50_ = 0.690 µM), incorporation of *para*-methoxy group 187b or *para*-fluoro group 187d significantly improved inhibition (IC_50_ = 0.475, 0.440 µM). Chloro group 187c decreased inhibition to 1.646 µM. In 187f–h (IC_50_ = 0.859–3.346 µM) with *N*-methyl thiazolidinone, groups at *para* position of the arylidine ring either electron donating (Methoxy) or withdrawing (chloro or fluoro) decreases α-glucosidase inhibition compared to unsubstituted phenyl 187e (IC_50_ = 0.3456 µM). Relative to 187i (IC_50_ = 2.025 µM), inhibitors 187j–l (IC_50_ = 0.447–1.276 µM) demonstrated enhanced inhibition, highlighting the favorable impact of *para*-substitution on the phenyl ring, as *para-*chloro substitution enhanced inhibition 4-times (187k, IC_50_ = 0.447 µM). Similarly, 187j, and 187l induced 2-times higher inhibition (IC_50_ = 1.085 and 1.276 µM). Rigid inhibitors 187a, 187e, and 187i declined potency than open chain analogue. 189a–b (IC_50_ = 1.833, 0.5991 µM) decreased one-fold inhibition, and 189c (IC_50_ = 0.9781 µM) decreased inhibition 2-times (Fig. 63).^84^

187b, 187d, 187e, and 187k being the most potent, were docked into the active pocket (PDB ID: 3W37) of α-glucosidase. Negative binding energies (−9.7085 to −11.0996 kcal mol^−1^) suggest desirable molecular interactions with enzyme. Acarbose (−9.9884 kcal mol^−1^) showed greater hydrogen bonds (Asp232, Ala234, Asn237, Arg552, Asp568, His626). Acarbose hydrophobic interactions include Phe236, Ile233, Trp432, Trp329, Met470, and Phe601. Inhibitors consistently formed two to three hydrogen bonds with the same amino acid, which were critical for stabilizing the complex. Thiazolidinone moiety formed hydrogen bond with Arg552. Interaction analysis revealed that in 187b (−9.7085 kcal mol^−1^), and 187d (−10.664 kcal mol^−1^), NH of thiazolidinone ring was anchored to Asp232 *via* hydrogen bond, whereas in 187e (−11.0996 kcal mol^−1^), NH_2_ of sulfonamide group engaged Asp232 residue. NH_2_ of sulfonamide in 187b, and 187d further interacted with Asn237 by hydrogen bond. Moreover, carbonyl group of the thiazolidinone moiety of 187d, and S=O of sulfonamide group of 187e established hydrogen bonds with Met470, and Ala234, respectively, contributing to stabilization. 187e showed the best binding (−11.0996 kcal/mol), and augments lowest IC_50_ value (0.3456 µM) against α-glucosidase. Furthermore, numerous hydrogen-π interactions were observed. Thus, benzylidene moiety of 187b, and 187d–e interacted Trp329, sulfonamide moiety in 187b interacted Asn237, and Ala234. In 187d, the same moiety interacted with Ile233, and Ala234, and thiazolidinone ring engaged Arg552. Furthermore, 187b, 187d, 187e, and 187k (−10.1650 kcal mol^−1^) established multiple hydrophobic contacts with residues such as Met470, Phe601, His626, Phe236, Trp432, and Ile233.^84^

**Scheme 31:**
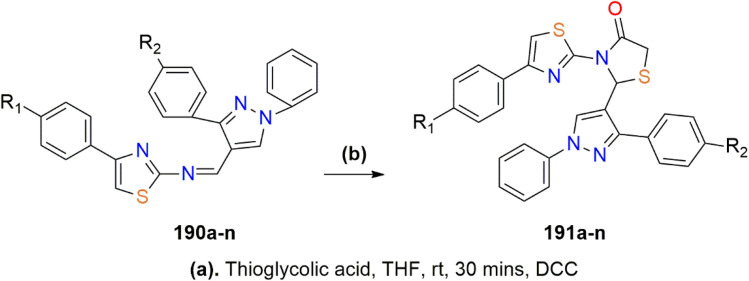
Synthesis of thiazolidin-4-ones 191a–n.

R. M. More *et al.* synthesized fourteen thiazolidin-4-ones 191a–n (Scheme 31). Thus, intermediates 190a–n were mixed *in situ* with thioglycolic acid, in THF at room temperature for 30 min, DCC was added gently. Upon completion, ice was added, solid was extracted from ethyl acetate, washed with 10% sodium bicarbonate, and crude product was purified by column chromatography (5–15% ethyl acetate in petroleum ether) to get thiazolidin-4-one 191a–n.^85^

**Fig. 64:**
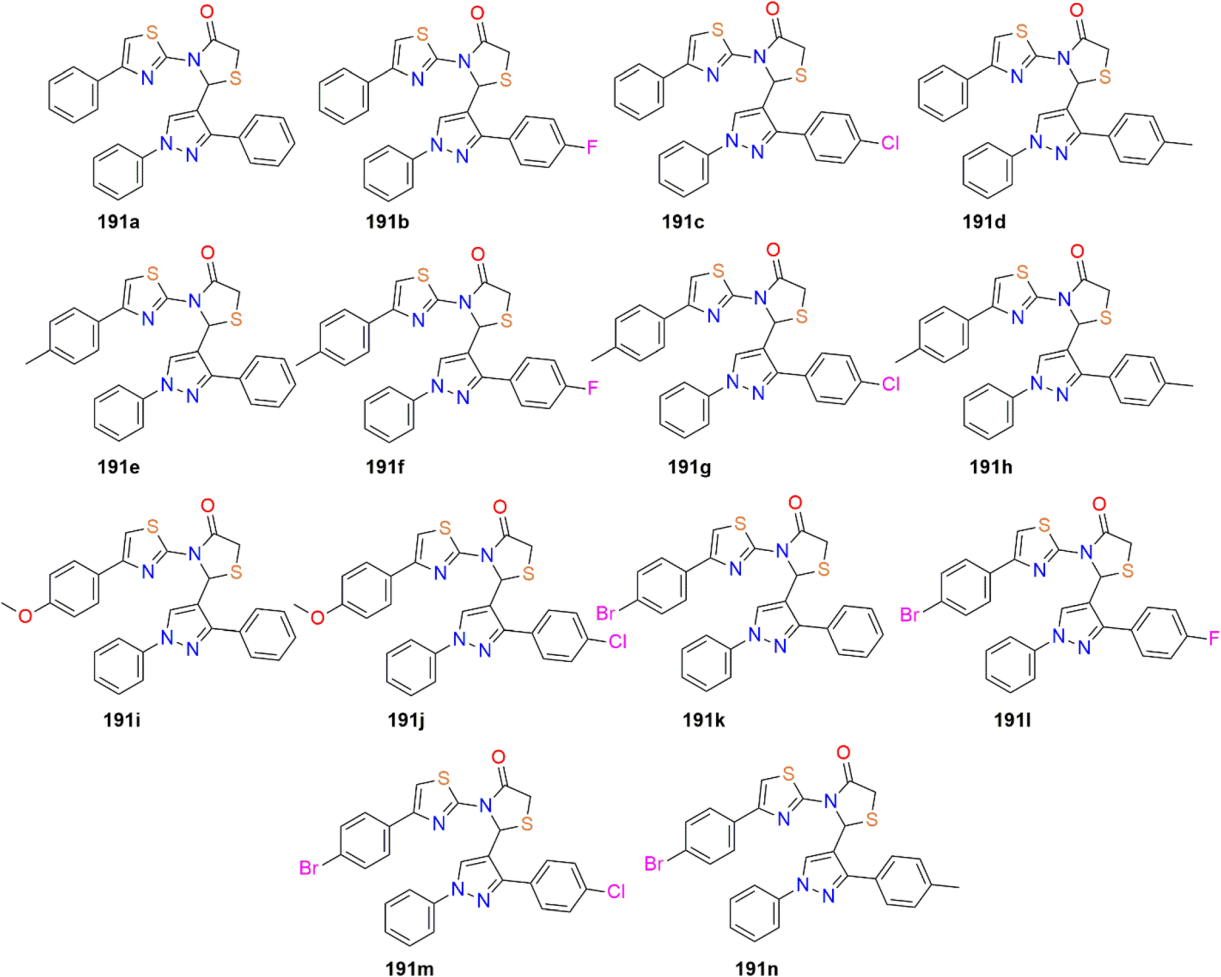
Structures of the potent thiazolidin-4-ones 191a–n.

191a–n were evaluated against α-amylase using Acarbose as drug. In terms of % inhibitions, 191a–c, and 191i–j possess comparable % inhibitions to Acarbose. In terms of their IC_50_ values, 191a showed highest inhibition (IC_50_ = 78.11 ± 0.01 µM), followed by 191j–k as better inhibitors (IC_50_ = 401.06 ± 0.01, 465.89 ± 0.02) than Acarbose (IC_50_ = 561.00 ± 0.01 µM). 191e, 191h–i, and 191l–m showed moderate inhibition (1429.0 ± 0.01–2654.80 ± 0.01 µM). 191c, 191f–g, and 191n were weak inhibitors (IC_50_ = 2791.89 ± 0.01, 3389.76 ± 0.05, 3139.69 ± 0.02, 2777.08 ± 0.04 µM) (Fig. 64).^85^

Simulations of 191a–b, 191e–f, and 191l showed stable interactions with active sites: 191e (10.7 kcal mol^−1^) > 191a = 191f (10.4 kcal mol^−1^) > 191l (10.2 kcal mol^−1^) > 191b (10.1 kcal mol^−1^). 191a showed electrostatic interaction of C-phenyl ring of pyrazole with Arg303 by π-anion interaction (4.06 Å), hydrophobic interactions include π–π stacked (Trp59), π–π T-shaped interaction (Trp58), and π–alkyl interaction (Agr303), followed by two halogen interactions with Asp356 (2.93, 3.31 Å) in 191b. Electrostatic interaction occur between C-phenyl ring of pyrazole and Arg356 (4.31 Å) *via* π–anion interaction. 191b established π-sulphur, π–π stacked interaction (Trp59), and π–π T-shaped interaction (Trp58). Thiazoline ring in 191e engaged in conventional hydrogen bond with S-atom of thiazoline ring, and Gln63 (3.08 Å). Electrostatic interactions were present *via* π–anion interaction between C-phenyl ring of pyrazole, and Asp356 (3.99 Å). Hydrophobic interaction occurred by π–π stacked interaction (Trp59), π–π T-shaped interaction (Trp58), and aryl rings of 191a, while two π–alkyl interactions were observed (His 299, Agr303). Similar interactions were observed with 191f, and 191a. Thiazole ring of 191l formed hydrogen bond with Gln63 (2.99 Å). Fluorine functionality in thiazolidin-4-one 191l showed two halogen interactions with Asp356 (3.35, 2.90 Å). C-phenyl ring of pyrazole in 191l formed electrostatic interaction with Asp356 (4.11 Å). 191l exhibited six hydrophobic interactions within the active site. Among these, C-phenyl ring of pyrazole formed π-σ contacts (His305), and π–π T-shaped interaction (Trp58). Thiazole ring established π–π stacked, and π–π T-shaped interaction (Trp58, Trp59). Additionally, C-phenyl ring of thiazole ring showed π–π T-shaped interaction with the receptor, and π–alkyl interaction was present with His299. MD suggests electrostatic interaction accounts for higher α-amylase inhibitory potential augmented by *in vitro* assay.^85^

DPPH FRSA was determined for 191a–n against Ascorbic acid (IC_50_ = 9.71 ± 0.02 µM). Thus, unsubstituted, and bromo-substituted phenyl ring of thiazole showed good DPPH FRSA. Bromine, due to its strong electron-withdrawing inductive effect, and moderate atomic size, exhibited the highest inhibition. As the halogen size increased, the inductive effect diminished, which in turn enhanced the free radical scavenging activity (IC_50_ = 191l–n: 52.03 ± 0.02, 41.39 ± 0.04, 56.29 ± 0.19 µM). Hydrogen atom at similar position possesses comparable FRSA (IC_50_ = 191b, 191d: 57.18 ± 0.06, 44.36 ± 0.07 µM). 191c, 191e, 191i, and 191j exhibited enhanced FRSA (IC_50_ = 551.50 ± 0.10, 699.00 ± 0.03, 543.00 ± 0.02, 742.90 ± 0.02 µM) owing to the presence of EDGs (Methyl, Methoxy). 191a, 191f–h, 191k displayed average inhibitions (IC_50_ = 83.31 ± 0.01–374.90 ± 0.05 µM).^85^

**Scheme 32:**
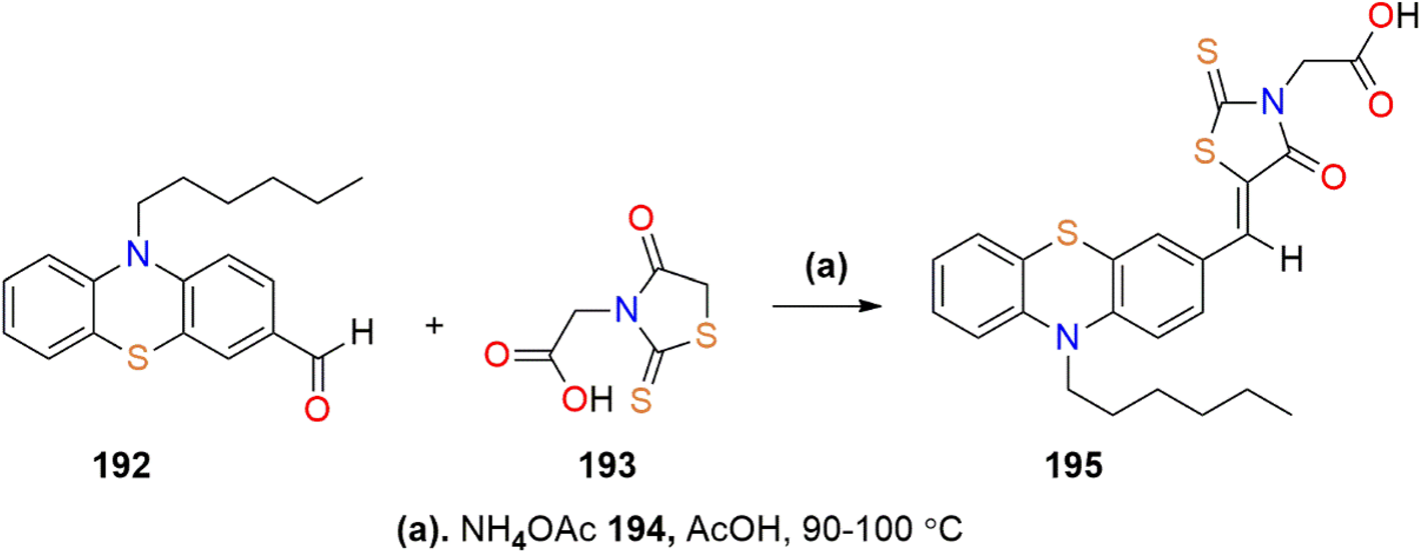
Synthesis of 2-(5-((10-hexyl-10H-phenothiazin-3-yl) methylene)-4-oxo-2-thioxothiazolidin-3-yl) acetic acid 195.

K. Sudhakar *et al.* synthesized 2-(5-((10-hexyl-10*H*-phenothiazin-3-yl) methylene)-4-oxo-2-thioxothiazolidin-3-yl) acetic acid (Scheme 32). Thus, phenothiazine-3-carbaldehyde 192, thioxothiazolidine acetic acid 193, and ammonium acetate 194 in acetic were refluxed at 90–100 °C for 12 h. Upon completion, the product was purified by column chromatography, in 30% *n*-hexane: EtOAc eluent system to get 195.^86^

DPPH degradation by 195 increased in dose dependent manner. 195 exhibited maximum DPPH scavenging (38.8 ± 0.6%-88.6 ± 4.0%). 195 exhibited maximum hydrogen peroxide (H_2_O_2_) scavenging property (74.10.2%). ABTS reducing properties of 195 were increased in dose dependent manner. Functional 195 exhibited maximum ABTS reducing potential (39.1 ± 1.2%-77.8 ± 6.4%). Functional 195 showed maximum Ferric reducing anti-oxidant power (FRAP) potential in the range of 16.0 ± 0.6%-43.6 ± 0.5%, strongly dependent upon concentration.^76^ Molecular docking analysis was performed against target protein (PDB code: 1HD2) with crystal structure of human and alkyl hydroperoxides. 195 ligand-protein interactions against target protein residues include LYS63 (N–H⋯O, 1.86 Å), LYS63 (C–H⋯O, 2.78 Å), and GLN68 (C–H⋯O, 2.73 Å), with associated higher binding energy (−240.3 kcal mol^−1^). Ascorbic acid as drug, established ligand–receptor interactions with constituent residues; LYS93 (O–H⋯O, 1.90 Å), LYS63 (C–H⋯O, 2.3 Å), GLY92 (O⋯H, 2.50 Å), GLN68 (C–H⋯O, 2.69 Å), and VAL70 (O–H⋯O, 3.00 Å), and showed higher interaction energy (−166.77 kcal mol^−1^). Thus, 195 possesses the potential to inhibit human, and alkyl hydroperoxides protein, and could be employed to counter oxidative stresses, and related complications.^86^

**Scheme 33:**
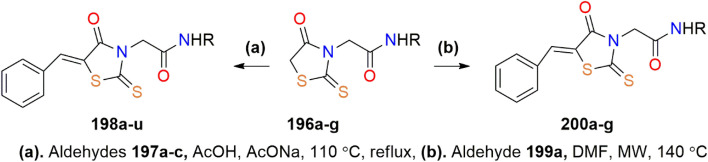
Synthesis of thioxothiazolidines 198a–u, and 200a–g.

A. Ravi *et al.* synthesized twenty-eight thioxothiazolidines 198a–u, and 200a–g. Thus, 2-(4-oxo-2-thioxothiazolidin-3-yl)acetamides 196a–g, and benzaldehydes 197a–c/199a were refluxed in acetic acid by Knoevenagel condensation, and microwave-assisted conditions in DMF at 140 °C to afford thioxothiazolidines 198a–u, and 200a–g in good yields (72–91%) (Scheme 33).^87^

**Fig. 65:**
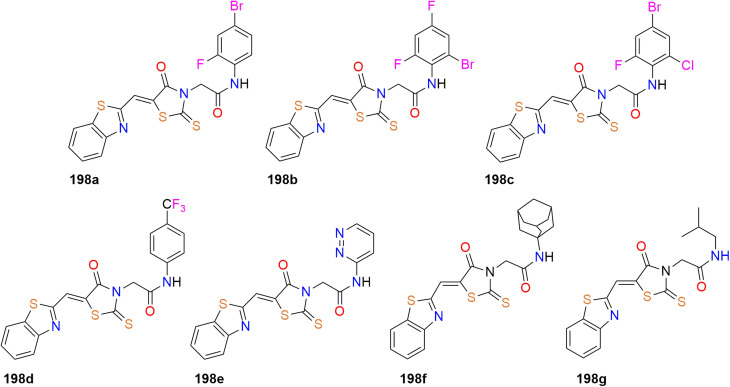
Structures of the potent thioxothiazolidines 198a–g.

198a–u, and 200a–g were evaluated against α-amylase, and α-glucosidase using Acarbose as drug (81.2%, IC_50_ = 950.9 ± 0.34 µM). Against α-glucosidase, no inhibitions were shown compared to Acarbose (76.4%), except 200c with very low inhibition (10.23 ± 1.9%). Against α-amylase enzyme in benzothiazole based inhibitors, 198d showed 234-folds higher inhibition (IC_50_ = 4.07 ± 0.01 µM) than drug. 198a served as the 2nd most potent inhibitor (IC_50_ = 128.07 ± 0.07 µM). 198c further decreased inhibition (IC_50_ = 167.21 ± 0.12 µM). 198b, and 198e–g with cyclic, and acyclic aliphatic/heteroaromatic groups have lower inhibition (29.4–43.2%) (Fig. 65).^87^

**Fig. 66:**
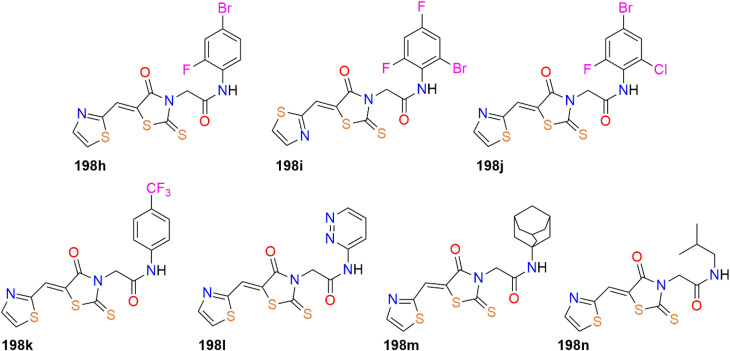
Structures of the potent thioxothiazolidines 198h–m.

In thiazole-based inhibitors, 198j showed highest inhibition (IC_50_ = 45.07 ± 0.05 µM), 21-folds higher than Acarbose. 198h–i, and 198k–n showed moderate inhibition (10.1–42.8%) (Fig. 66).^87^

**Fig. 67:**
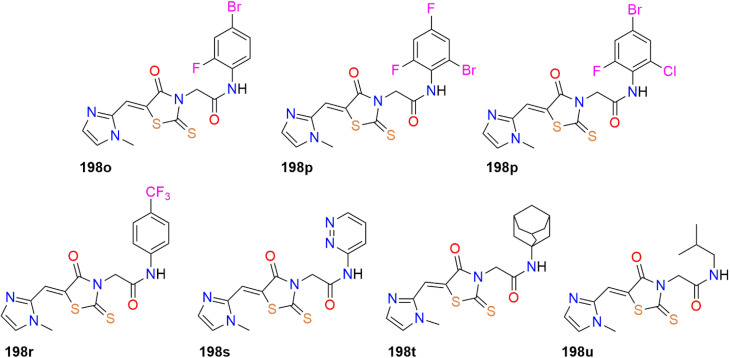
Structures of the potent thioxothiazolidines 198o–u.

In imidazole-based inhibitors, 198r showed the best inhibition (IC_50_ = 0.71 ± 0.01 µM), 1339-folds higher than Acarbose. 198p showed significant inhibition (IC_50_ = 129.34 ± 0.08 µM). 198o, 198q, and 198s–u were less potent α-amylase inhibitors (10.2–38.2%) (Fig. 67).^87^

**Fig. 68:**
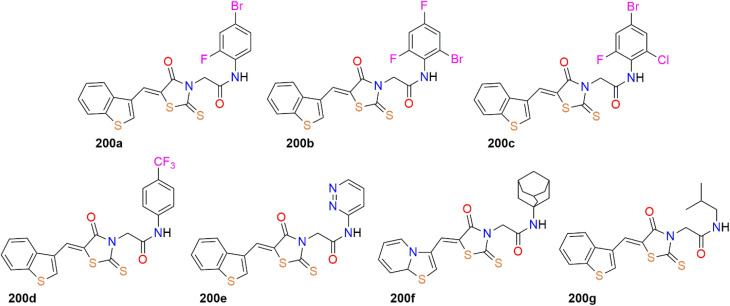
Structures of the potent thioxothiazolidines 200a–g.

In benzothiophene based inhibitors, 200d showed 68-folds higher inhibition (IC_50_ = 14.01 ± 0.01 µM), than Acarbose. 200c displayed remarkable inhibition (IC_50_ = 154.05 ± 0.04 µM) followed by 200b (IC_50_ = 189.69 ± 0.06 µM). 200a, and 200e–g, showed varied inhibitions (14.7–37.8%) (Fig. 68).^87^

198r being the most potent, was docked with α-amylase enzyme (PDB ID: 5E0F) using Acarbose as drug. 198r showed binding affinity (−7.2 kcal mol^−1^) for the catalytic active site of enzyme α-amylase. 198r formed hydrogen bonds with His299 (2.81 Å), and Gln63 (2.51 Å) within the pocket. Carbon hydrogen bonds were established with Thr163 (3.73 Å), and Gly104 (4.22 Å), hydrophobic π–alkyl contacts with Tyr62 (4.11 Å), Val107 (4.76 Å), and Ile51 (4.70 Å), π–sulfur interaction was observed with Trp59 (4.22 Å), and halogen bonds engaged residues like Asp197 (3.07 Å), and Asp300 (3.31 Å). These interactions modify the active-site pocket conformation, preventing the hydrolysis of starch. The favorable binding energy (−6.70 kcal mol^−1^) of Acarbose with α-amylase underscores its strong inhibitory activity against starch hydrolysis. Acarbose exhibited conventional hydrogen bonds engaging essential residues. Asp197 (2.52 Å), and Asp300 (2.70 Å) established hydrogen bonds with Acarbose. Glu233 (2.19 Å), and Tyr151 (2.01 Å) contribute to hydrogen bonding. His305 (1.91 Å), and Trp59 (1.89 Å) suggest additional stabilizing effects to enhance inhibition. Alkyl interactions with Ile235 (5.16 Å) provide hydrophobic stabilization, reinforcing structural integrity of the active site.^87^

**Scheme 34:**
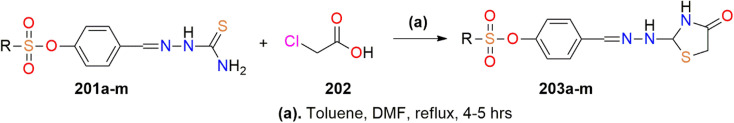
Synthesis of sulfonate esters of thiazolidin-4-one 203a–m.

R. Kaur, R. Kumar, N. Dogra *et al.* synthesized thirteen sulfonate esters of 2-(2-benzylidenehydrazono)thiazolidin-4-one (Scheme 34). Thus, thiosemicarbazone compounds 201a–m, and chloro acetic acid 202 were refluxed in toluene, and DMF as catalyst to get 4-thiazolidinones 203a–m.^88^

**Fig. 69:**
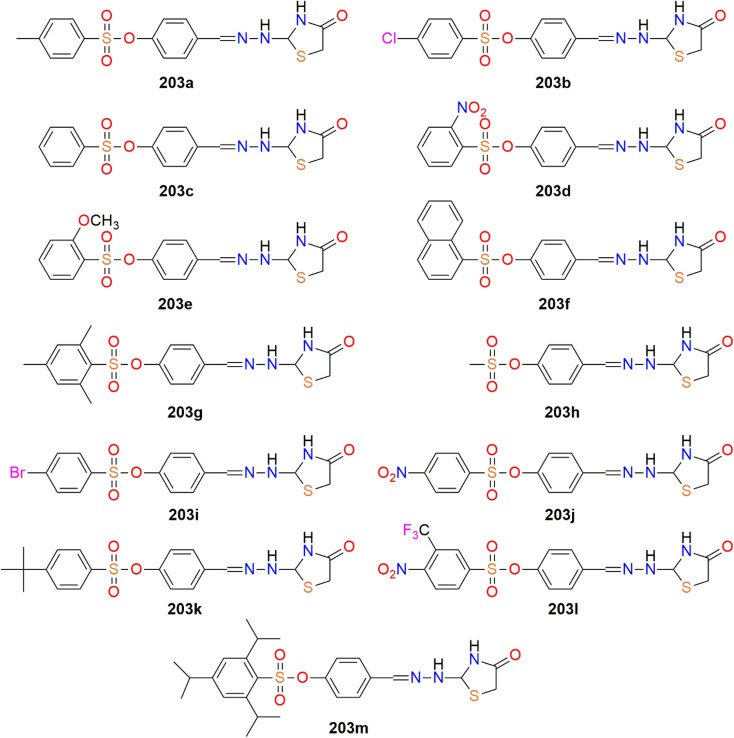
Structures of 2-(2-benzylidenehydrazono)thiazolidin-4-ones 203a–n.

203a–m were found to be variable α-glucosidase inhibitors (IC_50_ = 42.80 ± 0.48–599.04 ± 1.26 µM) compared to Acarbose (IC_50_ = 478.07 ± 1.53 µM). 203d (IC_50_ = 42.80 ± 0.48 µM) emerged as highly active, then 203m (IC_50_ = 48.19 ± 0.36 µM). Structure activity relationships depicted that:

• Aryl sulfonate group is crucial, alkyl sulfonate 203h decreases inhibition (IC_50_ = 599.04 ± 1.26 µM) .

• Substitution of aryl sulfonate better than naphthyl sulfonate, 203c (IC_50_ = 142.74 ± 0.51 µM), and 203f (IC_50_ = 198.71 ± 0.83 µM).

• Among aryl sulfonates, 203d showed highest inhibition, bearing hydrogen bond acceptor, and electron withdrawing nitro group at *ortho*-position,

• Bulky and electron releasing groups, 203m (IC_50_ = 48.19 ± 0.36 µM) favored inhibition at *ortho* and *para* positions. 203g (IC_50_ = 56.00 ± 0.32 µM) than 203a (IC_50_ = 91.06 ± 0.69 µM) (Fig. 69).

203d being most potent, was actively docked against the active site of α-glucosidase enzyme. Hydrogen bonding, salt bridges, and hydrophobic contacts exist between enzyme, and inhibitor. 203d has a snug fit (−5.1 kcal mol^−1^) within the active site. 4-Thiazolidinone ring penetrates cavity formed by Ala278, Arg212, Tyr71, Phe177, His348, and Arg439, along with catalytic residues Glu276, Asp214, and Asp349. 203d established hydrogen bonds with Arg212 and Glu276 through respective oxygen and NH groups, Arg312 forms hydrogen bonds with oxygen of nitro group, and second with oxygen atom of sulfonyl group. Hydrophobic interactions, and salt bridge between Glu 304 and nitrogen atom of nitro group stabilized enzyme–inhibitor complex. Hydrogen bonds contribute to better α-glucosidase inhibition of 203d.^88^

Ferric reducing assay assessed oxidative nature of 203a–m, and effectively reduced ferric to ferrous ions (EC_50_ = 62.76 ± 0.89–223.15 ± 1.45 µM), in comparision to Ascorbic acid (EC_50_ = 196.51 ± 0.48 µM). 203m served as the most potent inhibitor (EC_50_ = 62.76 ± 0.89 µM). Phenylsulfonate bearing groups having + I inductive effect favored reduction. Anti-oxidant nature of inhibitors 203a–n augment their potential anti-oxidant efficacy, to manage post prandial hyperglycemia, and related diabetic complications.^88^

### 2.5. Sulfonamide-based thiazoles

Sulfonamide-based thiazoles constitute a distinctive class of antidiabetic molecular hybrids that synergistically combine the well-known enzyme-inhibitory properties of sulfonamides with the metabolic regulatory potential of thiazole scaffold. This rational hybridization strategy enables a dual-target mode of action, simultaneously modulating intestinal carbohydrate digestion, and peripheral insulin sensitivity. These hybrids effectively inhibit key digestive enzymes, including α-glucosidase, and α-amylase, thereby slowing carbohydrate hydrolysis, and attenuating postprandial glucose excursions. In addition to their effects on glucose absorption, several thiazole-sulfonamide conjugates have demonstrated DPP-4 inhibitory activity, protecting incretin hormones such as GLP-1 from enzymatic degradation, and enhancing glucose-dependent insulin secretion. Notably, some derivatives also target the polyol pathway through inhibition of aldose reductase, preventing excessive sorbitol accumulation that contributes to diabetic cataracts, and neuropathy. Through this integrated modulation of digestive enzymes, incretin signaling, and complication-associated pathways, sulfonamide-based thiazole hybrids emerge as promising multifunctional scaffolds for comprehensive diabetes management.

**Scheme 35:**
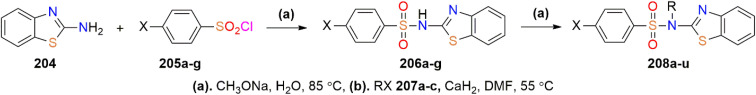
Synthesis of *N*-sulfonylated *N*-alkylated aminobenzothiazole 208a–u.

S. Khair-ul-Bariyah *et al.* synthesized twenty-one 2-aminobenzothiazoles 208a–u. Thus, seven *N*-Sulfonyl-2-aminobenzothiazoles 206a–g were synthesized, adding sodium acetate into water, followed by sulfonyl chloride 205a–g, and 2-aminobenzothiazole 204, stirring the mixture at 80–85 °C. Once completed, the solid was filtered, and recrystallized from ethanol. *N*-Sulfonamides reacted calcium hydride in DMF at 50–55 °C. Alkylating agents 207a–c were added after 30 min, and the blend was cooled, filtered and recrystallized from ethanol 208a–u (Scheme 35).^89^

**Fig. 70:**
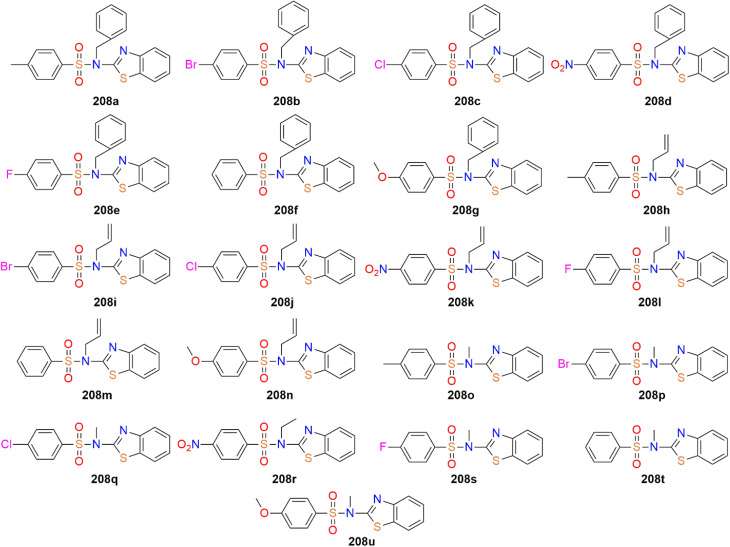
Structures of *N*-sulfonylated *N*-alkylated amino benzothiazoles 208a–u.

208a, 208g, 208k, 208o, and 208s possess significant α-glucosidase inhibition in a dose-dependent manner (79.35 ± 1.13, 139.53 ± 1.12, 172.16 ± 1.23, 179.35 ± 1.13, 97.36 ± 1.15 µM) using Acarbose as drug (375.82 ± 1.76). Molecular docking against human lysosomal acid-α-glucosidase enzyme (PDB ID: 5NN8) evaluated α-glucosidase inhibition using Acarbose as drug. Analysis of ligand-protein complex revealed interactions with the active site shows π–π T-shaped bond between benzene of benzothiazole, and benene of TRP 376 (4.56 Å), and π–π T-shaped link between π electrons of thiazole, and benzene of TRP 376 (4.82 Å). π-Sulfur attraction between sulfur of thiazole, and benene ring of TRP 376 (5.14 Å). Moreover, π-sulfur interactions include: benzene ring of TRP 481 interacts sulfur of thiazole ring (4.61 Å), and sulfonamide's sulfur atom (5.30 Å), π–π-T-shaped interaction between TRP 481 benzene, and ligand's benzyl moiety (4.93 Å), π–sulfur binding of MET 519 sulfur, and ligand's benzyl moiety's benzene ring interact (5.13 Å), conventional hydrogen bond (4.82 Å) between sulfonamide oxygen and NH of ARG 600 (Fig. 70).^89^

**Scheme 36:**
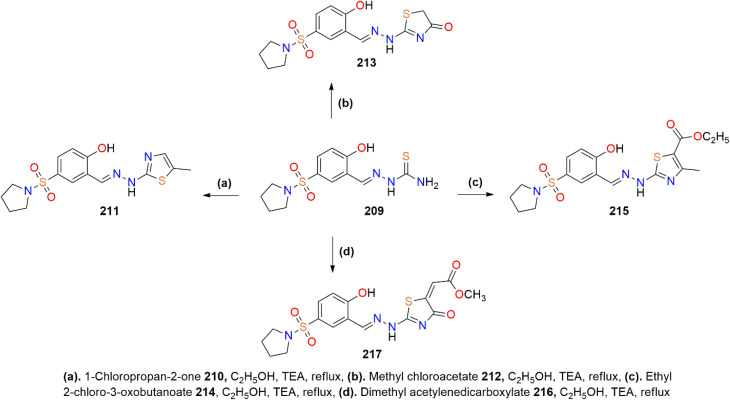
Synthesis of sulfonamide-based thiazoles 211, 213, 215, 217.

**Scheme 37:**
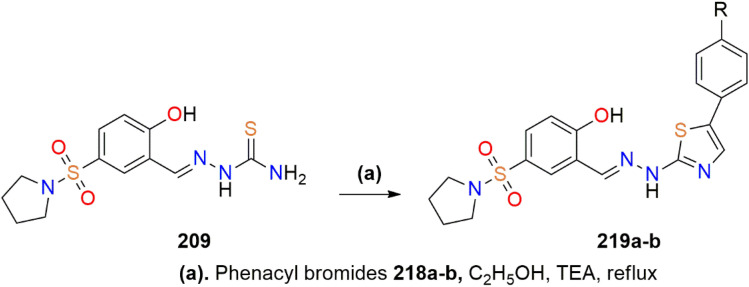
Synthesis of 5-aryl thiazoles 219a–b.

H. Khamees Thabet *et al.* synthesized six sulfonamide-based thiazoles. Thus, thiosemicarbazone 209 reacted 1-chloroacetone 210, methyl chloroacetate 212, ethyl 2-chloro-3-oxobutanoate 214, and dimethyl acetylene dicarboxylate 216 at reflux in ethanol, and TEA as catalyst. The reactions yields 2-((2-(5-methylthiazol-2-yl)hydrazineylidene)methyl)-4-(pyrrolidin-1-ylsulfonyl)phenol 211, 2-(2-(2-hydroxy-5-(pyrrolidin-1-ylsulfonyl)benzylidene)hydrazineyl) thiazol-4(5*H*)-one 213, ethyl 2-(2-(2-hydroxy-5-(pyrrolidin-1-ylsulfonyl)benzylidene)hydrazineyl)-4-methylthiazole-5-carboxylate 215, and methyl 2-(2-(2-(2-hydroxy-5-(pyrrolidin1-ylsulfonyl)benzylidene) hydrazineyl)-4-oxothiazol-5(4*H*)-ylidene)acetate 217 (Scheme 36). Later, thiosemicarbazone 209, and phenacyl bromides 218a–b were refluxed in ethanol, and TEA as catalyst to get 2-((2-(5-phenylthiazol-2-yl) hydrazineylidene) methyl)-4-(pyrrolidin-1-ylsulfonyl) phenols 219a–b (Scheme 37).^90^

211, 213, 215, 217, and 219a–b were analyzed in *in vitro* DPP-4 assay using Sitagliptin as drug. Thus, moderate inhibitions (40.66–75.62%) were observed compared to Sitagliptin (63.14%). In terms of IC_50_ (µM) values, cyclization to thiazole enhanced inhibition. 219a–b were significant inhibitors (IC_50_ = 2.75 ± 0.27, 2.51 ± 0.27 µM), than Sitagliptin (IC_50_ = 3.32 ± 0.22 µM). Enhanced inhibition was observed when a hydroxyl group occupied *para*-position of phenyl ring. Replacing phenyl ring with methyl in 211, and thiazol-4(5*H*)-one in 213, decreased DPP-4 inhibition (IC_50_ = 15.06 ± 0.59, 10.07 ± 0.06 µM). 217 bearing methyl acrylate group at 5^th^ position of thiazole, and thiazol-4(5*H*)-one moiety, decreased inhibition (IC_50_ = 14.61 ± 0.53 µM). Likewise, inhibition decreased in 215 (IC_50_ = 12.13 ± 0.32 µM) by combining ethyl ester, and methyl group at 5^th^, and 4^th^ positions of thiazole. SARs analysis suggests hydroxyl group as hydrophobic part at 5^th^ position of thiazole, increased DPP-4 inhibition. while polar groups (carbonyl, ester, acrylate) decreased DPP-4 inhibition.^90^

**Fig. 71:**
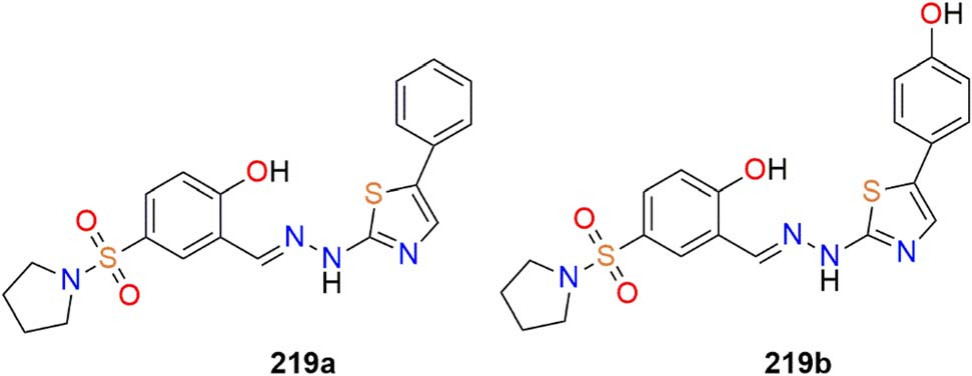
Structures of the potent 5-aryl thiazoles 219a–b.

219a–b being the most potent, were assessed for antidiabetic potential using Acarbose as drug. Thus, 219a exhibited higher inhibition (65.29%) than Acarbose (61.63%), and 219b have similar inhibition (61.55%) against α-glucosidase. Conversely, 219a showed higher inhibition (65.31%) than Acarbose, and 219b (64.25%) against α-amylase. In terms of IC_50_ values, 219a showed significant α-glucosidase inhibition (IC_50_ = 3.02 ± 0.23 µM), than 219b (IC_50_ = 3.34 ± 0.10 µM), and drug (IC_50_ = 3.05 ± 0.22 µM). 219b served as potent inhibitor (IC_50_ = 2.91 ± 0.23 µM), than Acarbose (IC_50_ = 2.99 ± 0.21 µM), and 219a (IC_50_ = 3.30 ± 0.16 µM) (Fig. 71).^90^

Simulations of 219a–b with α-glucosidase (PDB: 3W37) showed binding affinities (−6.03, −5.85 kcal mol^−1^). 219b established hydrogen bonds with Asp232, and Asp357 through hydroxyl group of aryl sulfonamide, and hydroxyl group at C-4 of 4-hydroxyphenyl at C-5 of thiazole (3.14, 3.34 Å). Trp329 established arene–hydrogen interaction with CH at C-2 of phenyl of thiazole (3.75 Å). Hydrophobic interactions include Ala234, Lys506, Ser474, Ile233, Phe476, Arg552, Asp568, and Asn475 with thiazole ring, sulfonyl pyrrolidine, and hydrazone linker. 219a established hydrogen bonds with sulfur of thiazole, and Asp (4.17, 3.18 Å). Thiazole formed arene–hydrogen interaction with Trp432 (4.25 Å). Trp239 revealed arene–hydrogen interaction with CH at C-3 of phenyl attached at C-5 of thiazole (3.98 Å). 219a showed hydrophobic interactions with thiazole, sulfonyl pyrrolidine, and hydrazones. Acarbose validated affinity (−6.39 kcal mol^−1^) by hydrogen bond with Ala234 (3.02 Å), ionic interactions with NH_2_ of hydroxyl amine and Asp568 (3.55, 2.78 Å), and hydrogen bonds with Met470 (3.83 Å), Asp232 (3.15 Å), Asp568 (3.55, 3.06 Å), andAsp469 (2.74, 2.89, 3.14 Å).^90^

Simulations of 219a–b against α-amylase active site (PDB:2QV4) were performed compared to Acarbose. 219a showed highest binding affinity than 219b (−6.27, −6.09 kcal mol^−1^). Acarbose showed binding affinity (−9.73 kcal mol^−1^) through hydrogen bonding between Ala106, and OH group at C-4 of pyranose, ionic bonding between NH_2_ of hexyl amine, and Asp300, hydrogen bonding of His305 (2.97 Å), and His201 (2.98 Å), and hydrogen bonds with Glu233 (3.05 Å), Asp300 (3.05 Å), and Asn105 (3.12 Å). 219a revealed hydrogen bonds between His201, and oxygen of sulfonyl group (3.33 Å), hydrogen bond between Asp197, and hydroxyl group of phenol (3.35 Å), and hydrogen bond between Trp62, and NH of hydrazone fragment (3.27 Å). Arene–arene interaction occurred between aryl ring at C-5 of thiazole, and Trp59 (3.93 Å). Hydrophobic interaction was established with 5-phenyl thiazole, and pyrrolidine (Tyr151, Leu165, Asp300, Thr163, Ile235, His305, and Asp356). 219b exhibited binding affinity (−6.27 kcal mol^−1^) with Glu233 (3.11 Å), and Gln63 (2.99 Å) through hydrogen bindings, arene–arene interaction (3.83 Å) of aryl sulfonyl with Trp59. Thus, 5-aryl thiazoles 219a–b developed crucial interactions within the target proteins comparable to Acarbose, as well as Sitagliptin and can serve as potent inhibitors.^90^

### 2.6. Imidazole based thiazoles

Imidazole-based thiazoles have emerged as highly potent hybrid antidiabetic agents, leveraging the synergistic effects of two privileged bioactive heterocyclic rings. These compounds primarily exert their antidiabetic action through the inhibition of key digestive enzymes, thereby delaying intestinal glucose absorption and reducing postprandial blood glucose excursions. Owing to their multitarget design, imidazole-thiazole hybrids influence multiple pathways relevant to the management of T2DM. Several reported derivatives exhibit strong α-glucosidase inhibitory activity, comparable to Acarbose. In addition, these hybrids effectively interact with the active site of α-amylase, suppressing the hydrolysis of dietary starch into absorbable sugars. Collectively, these mechanisms highlight the promise of imidazole-based thiazoles as multifunctional scaffolds for improved glycemic control with potential therapeutic advantages.

**Scheme 38:**
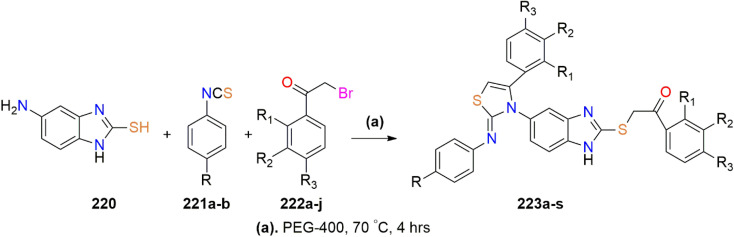
One-pot three component synthesis of imidazole based thiazole hybrids 223a–s.

R. Chedupaka *et al.* synthesized nineteen imidazole-based thiazoles (Schemes 38). Thus, benzimidazole 220, isothiocyanate 221a–b, and phenacyl bromides 222a–j, were refluxed for 3–5 h at 70 °C in PEG-400. Once completed, ice was added, filtered, and recrystallized from ethanol.^91^

**Fig. 72:**
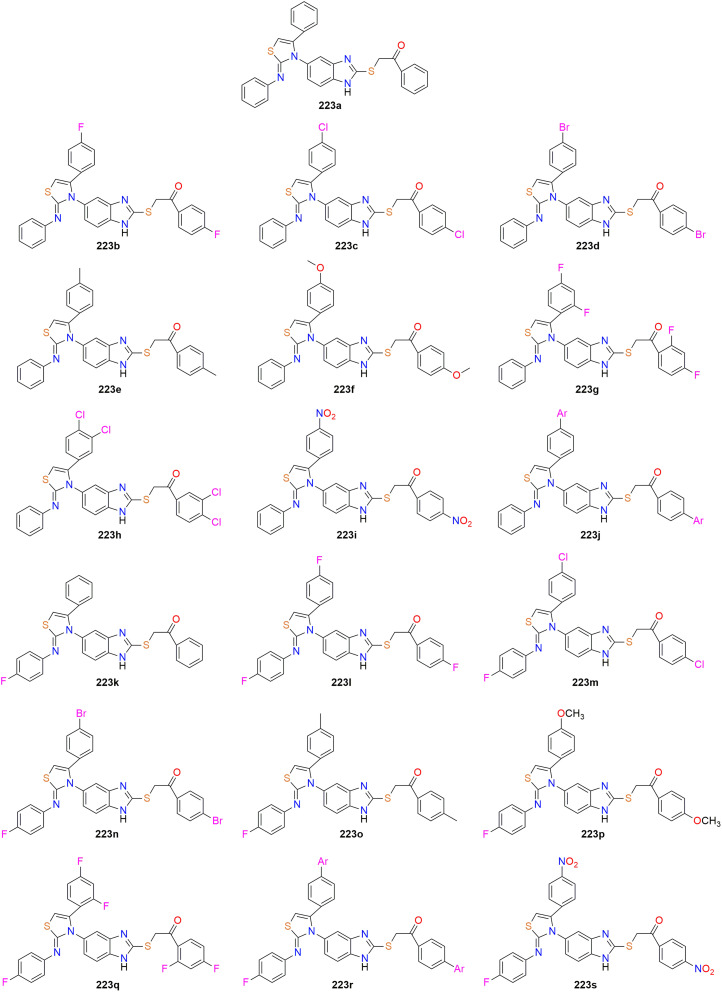
Structures of the potent thiazoles 223a–s.

223a–d, 223g–h, 223j–n, and 223r were screened against α-amylase using Acarbose as drug. Thus, varied inhibition (IC_50_ = 12.02 ± 0.51–44.57 ± 0.47 µg mL^−1^) was observed compared to Acarbose (IC_50_ = 11.88 ± 0.68 µg mL^−1^). 223b–d, 223h, and 223j showed significant inhibitions (IC_50_ = 12.02 ± 0.56, 12.25 ± 0.28, 12.74 ± 0.45, 17.83 ± 0.21, 19.10 ± 0.88 µg mL^−1^). 223k, 223l, and 223n showed weak inhibition (IC_50_ = 40.08 ± 0.56, 44.57 ± 0.47, 39.97 ± 0.64 µg mL^−1^). SARs suggest mono substitution (F, Cl, Br) on 4^th^ position of phenyl as most potent inhibitors (223b, 223c, 223d). Di-substituted halogens, 223g, and 223h are better inhibitors than 223b. 223j substituted at 4^th^ position showed significant inhibition. The presence of EDGs on 4^th^ position of phenyl ring, and unsubstituted scaffolds were weak inhibitors (Fig. 72).^91^

MD analyzed binding of 223a–s with montbretin A (PDB ID: 4W93). 223d being the most potent (IC_50_ = 12.02 ± 0.56 µg mL^−1^, LF dG −11.34 kcal mol^−1^) showed hydrogen bond between sulfur atom of thiazole ring, and Gln63 (2.54 Å), chlorophenyl group was enclosed in hydrophobic pocket of Trp59, and phenylamino group exhibited hydrophobic interaction with Tyr63, and Thr163. Chlorophenyl ring forms π–π contact with Tyr62. Benzimidazole formed hydrogen bond R195, and H299. 223c showed highest binding (LF dG −10.01 kcal/mol), and formed π–π interactions of Tyr62 with chlorophenyl, and hydrophobic interactions with Tyr62, and Thr163. 223a–s possess imidazole, and thiazole rings decorated with diverse groups showed strong binding with surrounding active site residues especially the core catalytic site Gln63, and Thr163 augment their superior inhibitory potential.^91^

**Scheme 39:**
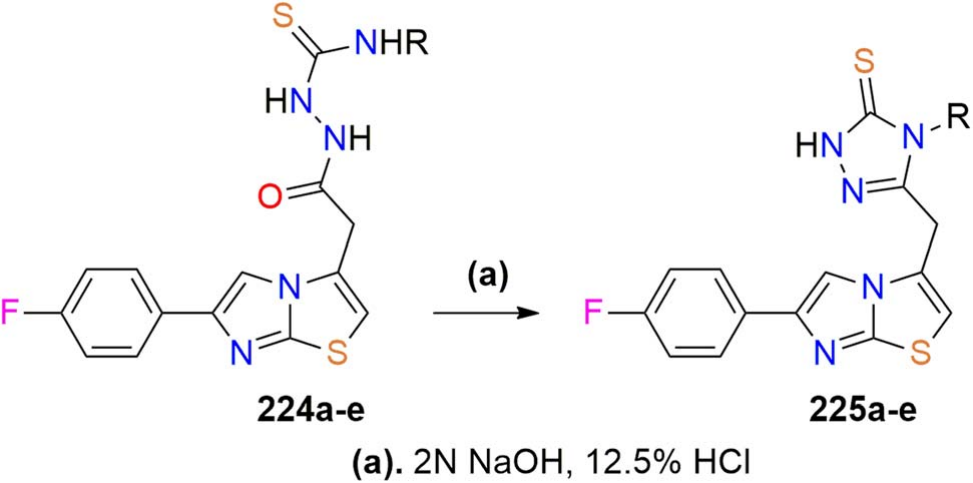
Synthesis of imidazole-based thiazoles 225a–e.

E.D. Dincel, G. Hasbal-Celikok, T. Yilmaz-Ozden *et al.* synthesized five imidazole-based thiazoles 225a–e. Imidazole-thiazole-based thiosemicarbazone compounds 224a–e were refluxed in 2N aqueous NaOH. Upon cooling, the mixture was acidified by HCl (12.5%) to get 225a–e in higher yield (86.40–78.07%) (Schemes 39).^92^

**Fig. 73:**
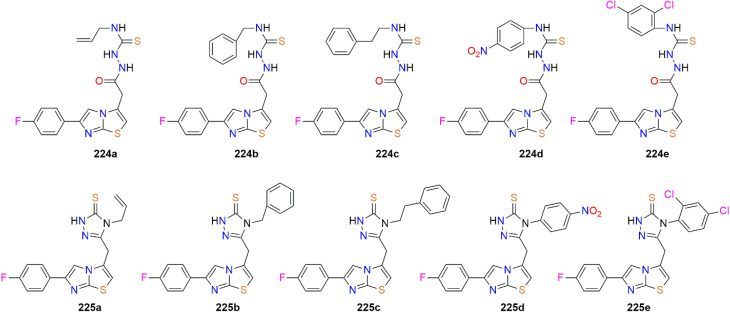
Structures of the potent imidazo[2,1-*b*]thiazoles 224a–225e.

224a–225e were screened against α-glucosidase using Acarbose as drug (IC_50_ = 214.71 ± 8.34 µM). 225c (IC_50_ = 4.54 ± 0.19 µM) emerged as 47-folds more potent than drug. 225a (IC_50_ = 145.26 ± 5.43 µM), 225d (IC_50_ = 155.68 ± 3.88 µM), 224e (IC_50_ = 172.99 ± 4.01 µM), 224d (IC_50_ = 208.06 ± 2.23 µM), and 225b (IC_50_ = 209.08 ± 11.82 µM) exhibited higher inhibitions than drug. 224a–c, and 225e lack any inhibitions, and were termed as inactive (Fig. 73).^92^

224a–225e, and Acarbose were docked into OBS (Orthosteric Ligand-Binding Site), and ALBS1-4 (Allosteric Ligand-Binding Site 1–4). 225c being the most potent, formed six π–π stacking with PHE 157 in OBS (−6.990 kcal mol^−1^), two hydrogen bonds with GLN 66, and two π-cation interactions with LYS 410 in ALBS3 (−4.284 kcal mol^−1^). 225c formed hydrogen bonds with LYS 370, two hydrogen bonds with ASP 485, and two π–π stackings with TYR 563 in ALBS4 (−1.357 kcal mol^−1^) suggesting 225c as diverse inhibitor. Superpositions of 224a–225e, and Acarbose in binding sites of SCM displayed similar orientation and conformation. Acarbose received higher docking scores than inhibitors, though lesser potent *in vitro* inhibitor than 224a–225e, indicating interactions with amino acids holds significance than scores.^92^

### 2.7. Amide based thiazoles

Amide functionality is widely favored in drug design due to their chemical stability, peptide-bond mimicry, and strong hydrogen-bonding capacity with biological macromolecules, all of which contribute to favorable pharmacokinetic properties, and enhanced drug-likeness. When integrated with thiazole scaffold, amide groups generate structurally versatile pharmacophores whose biological activity can be precisely modulated through strategic functional substitutions. The polar amide linker strengthens critical hydrogen-bonding interactions with target proteins, while fine-tuning of lipophilicity, and metabolic stability governs target engagement, and potency. In antidiabetic drug development, amide-based thiazoles act as multifunctional molecular hybrids capable of interacting with multiple metabolic targets. These compounds effectively inhibit key carbohydrate-hydrolyzing enzymes, thereby reducing intestinal glucose absorption, and controlling postprandial hyperglycemia. Several derivatives demonstrate affinity for DPP-4, preventing incretin hormone degradation, enhancing glucose-dependent insulin secretion. Beyond enzyme inhibition, selected amide-linked thiazole derivatives also exhibited significant anti-oxidant activity, demonstrating strong capacity to neutralize free radicals DPPH assay. This anti-oxidant potential is particularly relevant in diabetes management, as oxidative stress plays a crucial role in β-cell dysfunction, and the progression of diabetic complications. Collectively, the incorporation of amide linkages within the thiazole framework represents a rational, and effective strategy for the development of non-insulin-dependent antidiabetic agents with improved efficacy, selectivity, and balanced pharmacological profiles.

**Scheme 40:**
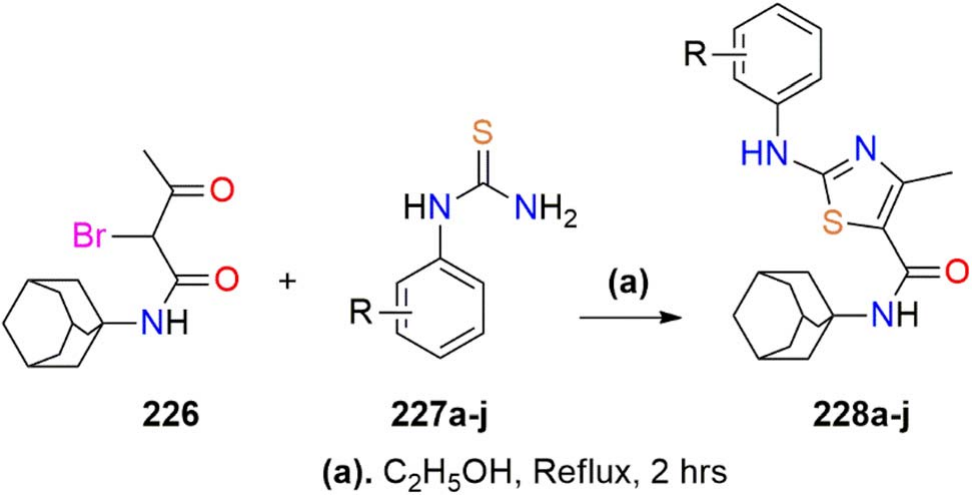
Synthesis of amantadine-based *N*-aryl amino thiazoles 228a–j.

F. T. Zahra *et al.* synthesized ten amantadine clubbed *N*-aryl amino thiazoles (Scheme 40). Thus, *N*-(adamantyl)-2-bromo-3-oxobutanamide 226, cyclized phenyl thioureas 227a–j in ethanol at reflux for 2 hours. The solid after aqueous workup, was filtered, and recrystallized from ethanol to get amantadine-based *N*-aryl amino thiazoles 228a–j.^93^

**Fig. 74:**
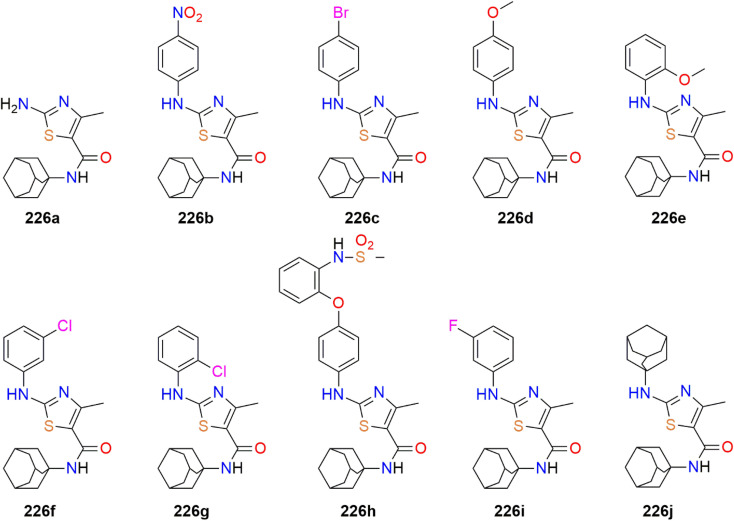
Structures of the synthesized amantadine clubbed *N*-aryl amino thiazoles.

228a–j were screened against α-glucosidase, using Acarbose as drug (IC_50_ = 1.21 ± 0.16 mM). Generally, good inhibition was observed against α-glucosidase. 228d served as the most potent inhibitor (IC_50_ = 38.73 ± 0.80 mM), followed by 228e, 228a, and 228c (IC_50_ = 41.63 ± 0.26, 72.12 ± 0.11, 98.65 ± 0.70 mM). Molecular docking suggests, 228a, 228c, and 228e possess strongest affinity, with lowest energy (−3.6, −0.7, −1.5 kcal mol^−1^), showing dual interaction with ARG609 (3.5 Å), ARG-587 (2.5 Å), and THR-384 (3.1 Å). In α-amylase assay, using Acarbose as drug (IC_50_ = 5.17 ± 0.25 mM), 228a, and 228d showed moderate inhibition (IC_50_ = 118.3 ± 0.71, 97.37 ± 1.53 mM). MD suggests, 228a, and 228d showed strongest interactions (−6.1, −1.1 kcal mol^−1^). 228a showed dual interaction with SER-4 (2.9 Å), and THR6 (3.0 Å). 228d showed one interaction with ARG-420 (2.8 Å). Thus, based on their strong inhibitory effects against α-amylase, and α-glucosidase, the screened hybrids 228a–j were found to be flexible towards the development of novel, and potent anti-diabetic drugs.^93^ DPPH FRSA assessed oxidative potential using Ascorbic acid as drug (IC_50_ = 0.892 ± 0.36 mM). 228d showed highest inhibition (IC_50_ = 19.19 ± 0.37 mM) than 228h (IC_50_ = 64.1 ± 0.7 mM). 228a, 228b, 228j, and 228i showed comparatively lower activity (IC_50_ = 130–157 mM), and rest of the inhibitors exhibited moderate DPPH scavenging (IC_50_ = 80–120 mM) (Fig. 74).^93^

**Scheme 41:**
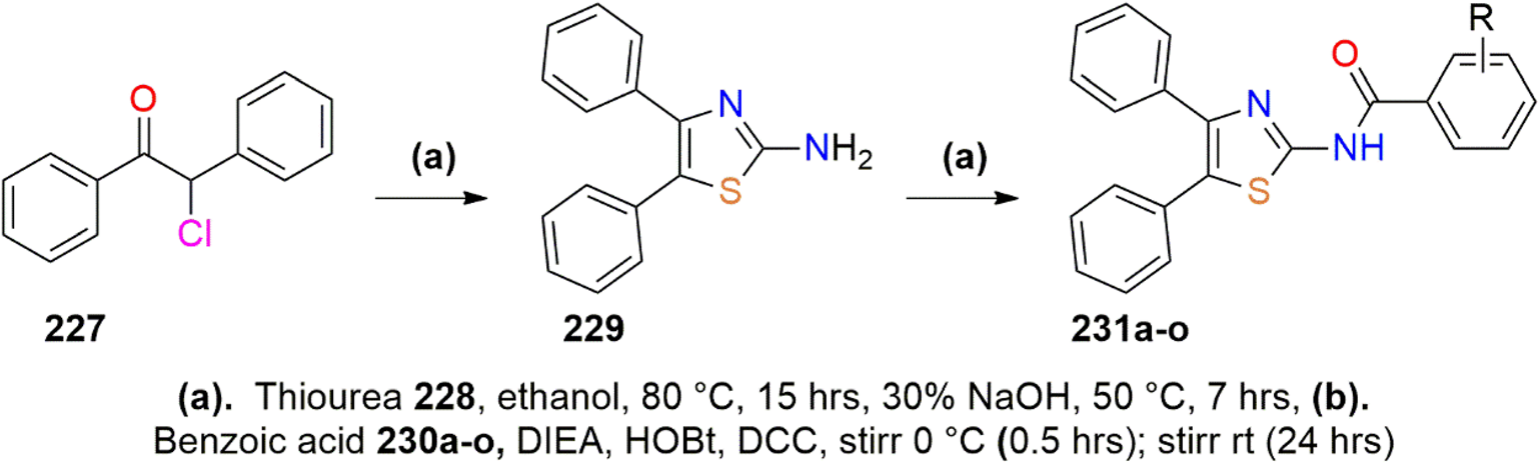
Synthetic processes of amide-based thiazoles 231a–o.

M. Fan *et al.* synthesized fifteen amide-based thiazoles 231a–o. Intermediate 227, and thiourea 228 were reacted at 80 °C in ethanol for 15 hr. Upon completion, ethanol was rotary evaporated, solid was added 30% NaOH solution, and stirred at 50 °C for 7 h. Later, the solid was washed, yielding 229 by recrystallization from ethanol. Finally, benzoic acid 230a–o, DIPEA, and HOBt in DCM were stirred in ice bath, adding DCC, continuously stirring for 15 min. Later, 2-aminothiazole 229 was poured, and stirred for half an hour under ice bath, at room temperature. Once completed, the mixture was washed, extracted from EtOAc, drying the organic extracts with Na_2_SO_4_. 231a–o were obtained by filtration, concentration, and purification using chromatography (Scheme 41).^94^

**Fig. 75:**
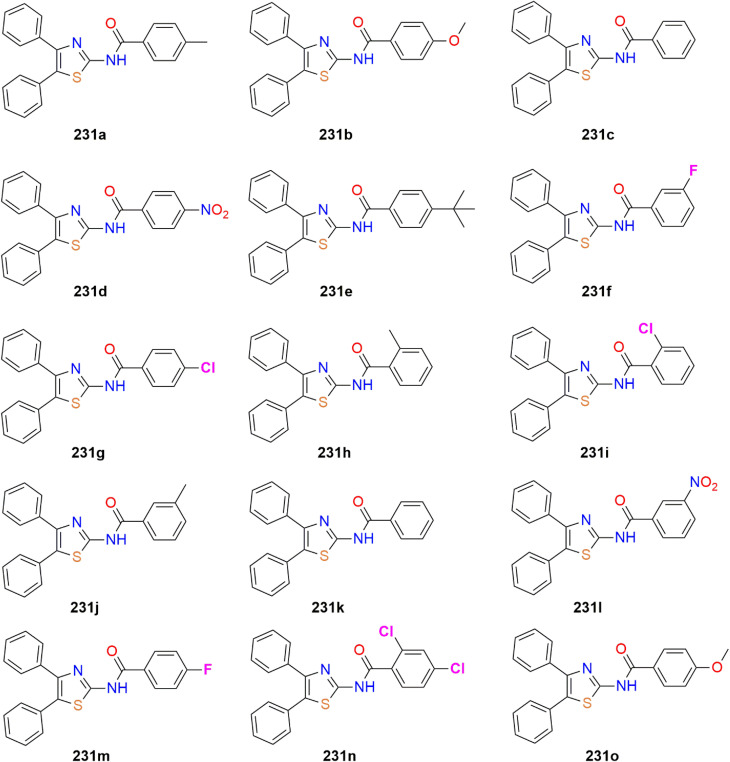
Structures of the synthesized amide-based thiazoles 231a–o.

231a–o were screened against α-glucosidase using Acarbose as drug. 231a–o inhibited α-glucosidase (IC_50_ = 12.18 ± 0.08–79.34 ± 1.30 µM) compared to Acarbose (IC_50_ = 774.69 ± 11.65 µM). 231c show highest inhibitory potential (IC_50_ = 12.18 ± 0.08 µM) against α-glucosidase. SARs suggest, 231b (IC_50_ = 25.30 ± 0.63 µM), 231h (IC_50_ = 38.83 ± 0.96 µM), and 231i (IC_50_ = 44.77 ± 0.92 µM) exhibited higher inhibition than 231k (IC_50_ = 49.07 ± 0.71 µM). The presence of electron withdrawing, and donating groups enhanced inhibition at *ortho*-position. 231d (IC_50_ = 20.29 ± 0.18 µM), and 231l (IC_50_ = 45.01 ± 0.90 µM) indicated *para*-position as better than *meta*-position for EWG. 231a (IC_50_ = 39.60 ± 0.91 µM), and 231e (IC_50_ = 22.68 ± 0.19 µM), suggests bulky groups favor inhibitions. 231c with naphthalene ring showed the most potent inhibitory effect. 231n containing chloro group (IC_50_ = 40.85 ± 1.02 µM) was higher than 231g (IC_50_ = 43.68 ± 0.31 µM), which means inhibition increased with increase in the number of chlorine atoms. 231a (IC_50_ = 39.60 ± 0.91 µM), 231h (IC_50_ = 38.83 ± 0.96 µM), and 231j (IC_50_ = 48.94 ± 2.40 µM), with methyl group at *para*-position increased inhibition as: 4-CH_3_>2-CH_3_>3-CH_3_. Thus;

• Inhibitions improved with EWGs, and EDGs except fluorine.

• Inhibitions improved with introduction of bulky groups.

• Inhibitions increased with increase in number of substituents (Fig. 75).^94^

**Scheme 42:**
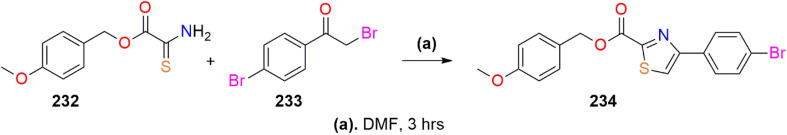
Synthesis of 4-(4-bromophenyl)thiazole-2-carboxylate 234.

N. S *et al.* synthesized 4-(4-bromophenyl)thiazole-2-carboxylate 234. Equimolar 4-methoxybenzyl 2-amino-2-thioxoacetate 232, and 2-bromo-phenacyl bromide 233 in DMF, were stirred for 3 hr. Upon completion, the mixture was extracted with ethyl acetate, dried over Na_2_SO_4_, and purified by column chromatography in hexane/ethyl acetate (88 : 12) eluent to afford 234 (Scheme 42).^95^

234 was evaluated in DPPH FRSA using Ascorbic acid as drug. 234 served as potent anti-oxidant (IC_50_ = 199 µg mL^−1^) compared to drug. 234 exhibited strong binding when docked into the cytochrome c peroxidase coordinates, indicating good binding score (−8.5 kcal mol^−1^), and potential inhibition. 234 interacts with nineteen favorable residues (MET172, VAL154, TYR187, THR234, ASP146, PRO145, THR180, PHE191, SER182, LEU177, PRO44, ARG48, LYS179, LEU232, ALA147, HIZS175, PHE158, TRP51, HIS181). Oxygen atom, and centroid of 3H-pyrole ring of PRO145, formed C–H bond, and π-alkyl interaction with carbon of 2-methoxy-2-methylpropane moiety (3.21, 5.30 Å). Oxygen attached to 2-methoxy-2-methylpropane moiety formed conventional H-bond with ARG48 (2.39 Å). ARG48 formed two π-cation interactions with centroid of *tert*-butoxybenzene and centroid of 4-methyl-4,5-dihydrothiazole (3.48, 4.81 Å). Hydrogen atom attached to oxygen atom of THR234 formed π–donor hydrogen bond with centroid of bromo benzene (3.08 Å). π–σ Interaction was observed between ALA147, and centroid of 4-methyl-4,5-dihydrothiazole moiety (3.83 Å). Further, π–alkyl, and alkyl interactions such as VAL154, MET172, and PHE158 were observed between 234, and amino acid residues (4.46, 4.51, 5.20 Å). van der Waals interactions, LEU177, TYR187, PHE191, THR180, ASP146, SER182, TRP51, LYS179, and HIS181 were observed between CCP protein and 234.^95^

**Scheme 43:**
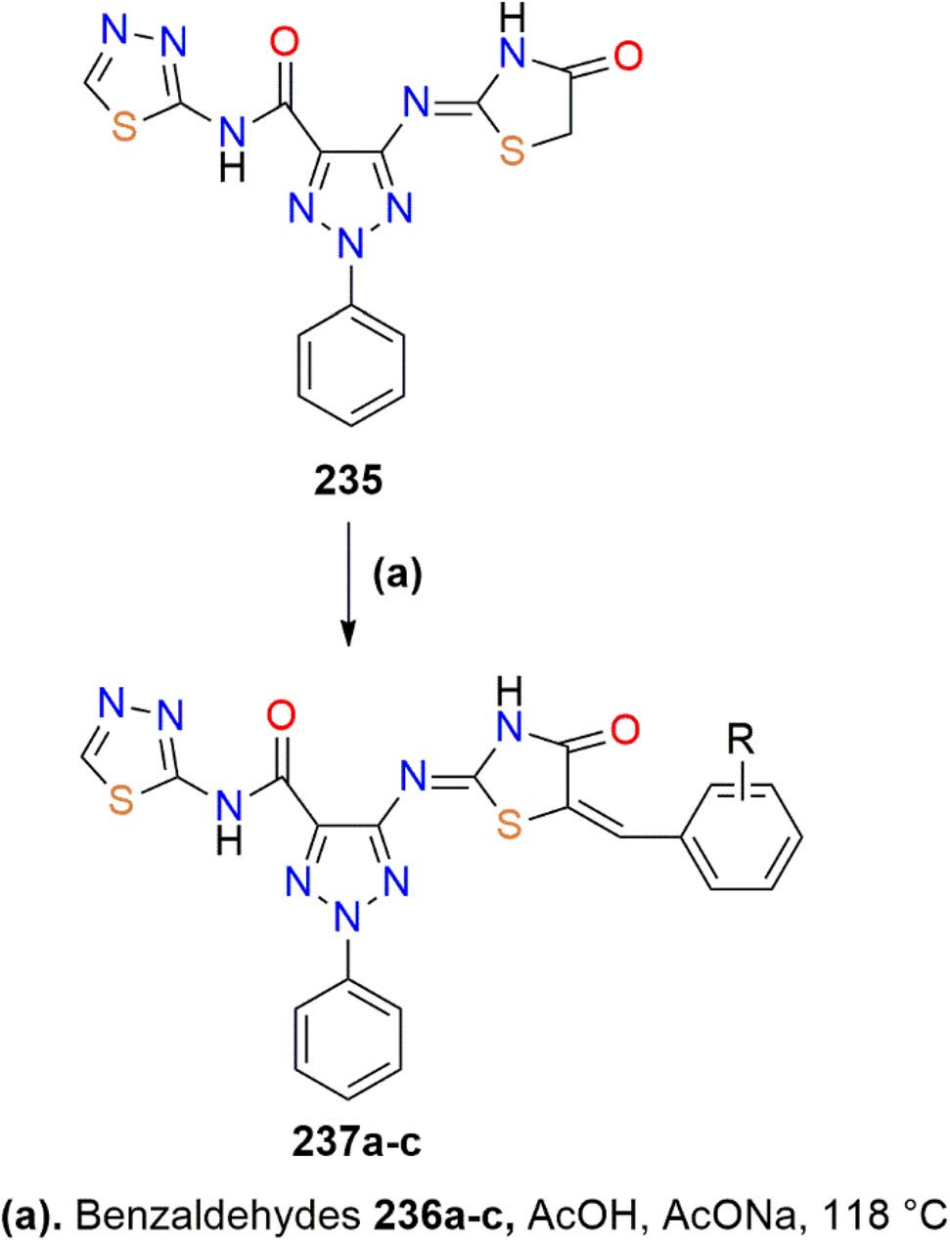
Synthesis of *N*-(thiadiazolyl)-triazole-4-carboxamides 237a–c.

S. Abu-Melha *et al.* synthesized three thiadiazole-triazole based thiazolones 237a–c (Scheme 43). Briefly, thiazolidine-4-one 235 dissolved in acetic acid, was added benzaldehydes 236a–c, and sodium acetate. The reaction mixture was refluxed at 118 °C for 4 h, and upon completion, was solidified, collected, and recrystallized from acetic acid.^96^

**Fig. 76:**
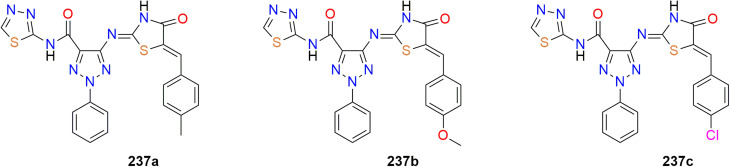
Structures of the synthesized thiadiazole-triazole-based thiazolones.

237a–c were subjected to anti-oxidant evaluation in DPP FRSA, using Vitamin C, and BHT as drugs (IC_50_ = 22.24 ± 0.12, 26.37 ± 0.04 µg mL^−1^). In terms of % inhibition at different concentrations (2.5, 5.0, 10.0 µg mL^−1^), 237a–c showed dose dependent free radical scavenging effects, which increased with increase in concentration, though less potent (33.36, 29.41, 26.33%) than Vitamin C (7.15%), and BHT (14.62%). In terms of their IC_50_ values, thiadiazole-triazole hybrids 237a–c revealed advanced inhibitions (IC_50_ = 25.45 ± 0.29, 22.63 ± 0.09, 26.37 ± 0.04 µg mL^−1^). SAR analysis of thiadiazolo-triazole hybrids 237a–c showed superior anti-oxidant potential owing to the presence of substituted amino-triazole moiety. 237a–c possess thiadiazole containing thiazolone ring, which transfer substantial inhibitory potential, coupled with triazole moiety associated with enhanced anti-oxidant potential (Fig. 76).^96^

**Scheme 44:**
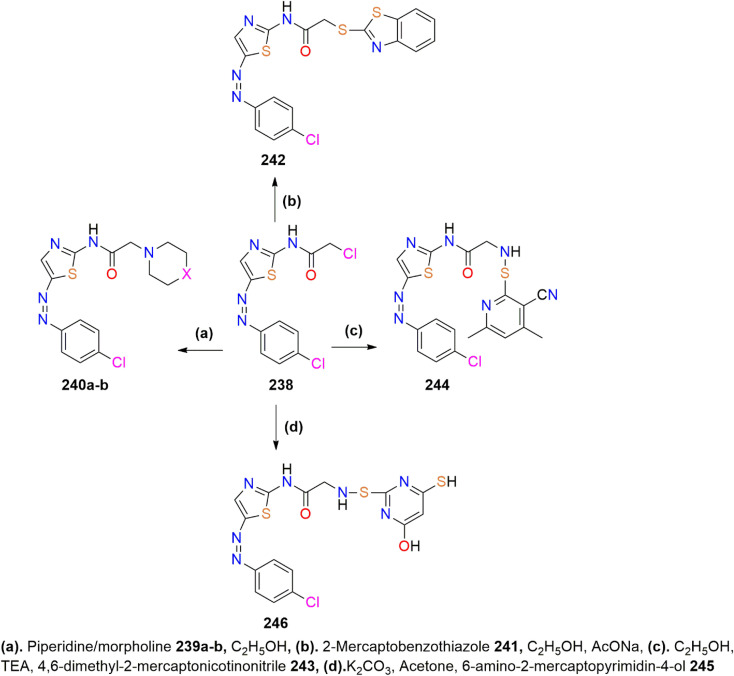
Synthesis of 5-(4-chlorophenylazo)-2-(2-substitutedacetamido)thiazoles 240a–b, 242, 244, 246.

S. Abu-Melha synthesized 5-(4-chlorophenylazo)-2-(2-substitutedacetamido)thiazoles 240a–b, 242, 244, 246. Thus 238 was dissolved in ethanol, mixed with piperidine/morpholine 239a–b, refluxed for 2 h, and recrystallized in ethanol to 5-(4-chlorophenylazo)-2-(2-substituted-acetamido)thiazoles 240a–b. Later, 2-chloroacetamido-thiazole 238 in ethanol, 2-mercaptobenzothiazole 241, and sodium acetate, were refluxed for 2 h, cooled, and collected to get 2-((benzothiazol-2-ylthio)acetamido)-5-(4-chloro-phenylazo)thiazole 242. A suspension of 238 in ethanol containing TEA, and 4,6-dimethyl-2-mercaptonicotinonitrile 243 was refluxed for 2 h, and recrystallized in ethanol to get sulfide *N*-(5-(4-chlorophenylazo)thiazol-2-yl)-2-((3-cyano-4,6-dimethylpyridin-2-yl)thio)acetamide 244. Later, 238, 6-amino-2-mercaptopyrimidin-4-ol 245, and potassium carbonate were stirred in acetone at 25–30 °C for 6 h. Upon dilution, solids were collected, and recrystallized from 2-((4-amino-6-hydroxypyrimidin-2-yl)thio)-*N*-(5-(4-chlorophenylazo)thiazol-2-yl)acetamide 246 (Scheme 44).^97^

**Fig. 77:**
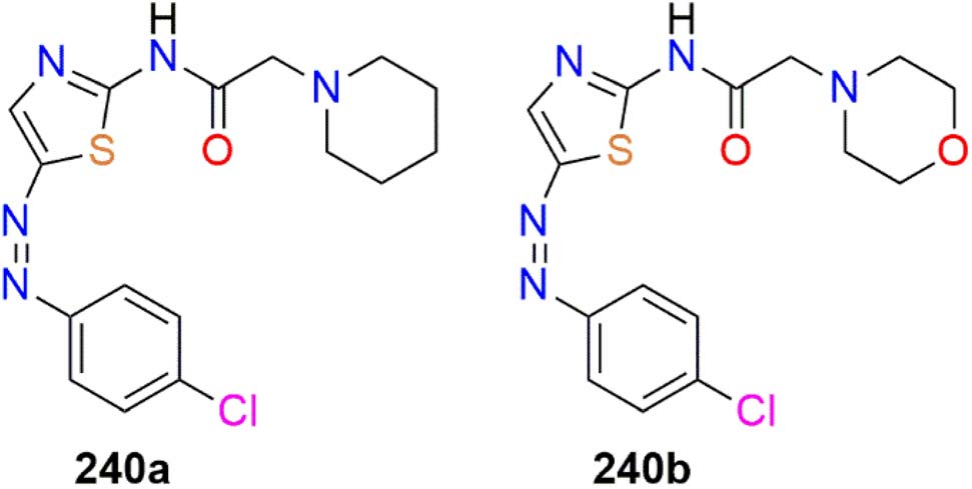
Structures of the synthesized acetamide-thiazole hybrids.

DPPH FRSA assessed anti-oxidant potential using Butylated hydroxytoluene (BHT), and Ascorbic acid as drugs (IC_50_ = 26.32 ± 0.17, 23.02 ± 0.01 µg mL^−1^). Thus, oxidative nature of thiazole-based inhibitors enhanced due to greater local electron density across S atom. 242, 244, 246 showed promising inhibition (IC_50_ = 32.26 ± 0.39, 28.43 ± 0.75, 24.17 ± 0.69 µg mL^−1^). 240a–b showed moderate (IC_50_ = 39.52 ± 0.62, 37.02 ± 0.43 µg mL^−1^), and 238 revealed lower inhibition (IC_50_ = 40.27 ± 1.54 µg mL^−1^). SARs suggest thiazolyl acetamides as good anti-oxidants due to the thiazole ring. 242, 244, 246 are potent inhibitors due to sulfide moiety. 246 showed highest inhibition owing to the presence of hydroxy/aminopyrimidine ring. 244 possess sulfide linkage, and cyanopyridine moiety that introduce an anti-oxidant power, while 242 has benzothiazole ring which possess anti-oxidant effects (Fig. 77).^97^

Molecular docking analyzed 240a–b, 242, 244, 246 with protein (PDB Code-2Y9X). 240a showed hydrogen-acceptor of binding (−6.14311 kcal mol^−1^) between O-atom of carbonyl group with Lys 379 (3.35 Å). 240b showed two different intermolecular hydrogen bonds (−6.3046 kcal mol^−1^) between S-atom of thiazole moiety with Glu 359 (3.69 Å), and O-atom of carbonyl group with Thr 308 (2.99 Å). Sulfide inhibitor 242 showed good binding (−6.3934 kcal mol^−1^) with two dissimilar contacts *i.e.*, two hydrogen bonds between Glu359, with S-atom of thiazole ring (3.06 Å), and S atom of sulfide moiety (3.00 Å). Second type includes two π–π stick interactions between benzene ring of phenyl azo moiety with Lys18 (4.43 Å), and benzene ring of benzothiazole with Thr308 (4.36 Å). 244 received reasonable energy (−6.5735 kcal mol^−1^) from four hydrogen acceptors, between S-atom of thiazole, and Glu359 (3.95 Å), O-atom of carbonyl with Lys379 (2.91 Å), N-atom of nitrile, and Gln 307 (3.49 Å), and hydrogen-acceptor between N of nitrile with Asp312 (3.80 Å). Sulfide 246 showed three intermolecular hydrogen bonds (−7.2835 kcal mol^−1^), between S-atom of thiazole with Glu359 (3.59 Å), O-atom of hydroxyl with Asp312 (2.99 Å), and N-atom of amino with Glu356 (2.92 Å), and one π–hydrogen interaction between pyrimidine with Tyr311 (4.14 Å). Thus, docking results correlate well with the results obtained from *in vitro* anti-oxidant assay.^97^

**Scheme 45:**
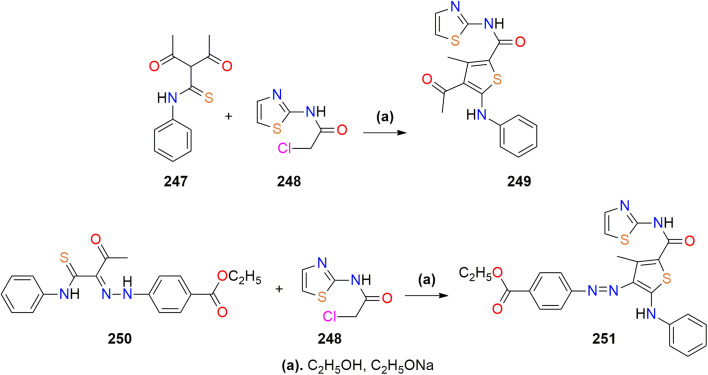
Synthesis of 3-methyl-5-(phenylamino)-*N*-(thiazol-2-yl)thiophene-2-carboxamides 249-251.

**Scheme 46:**
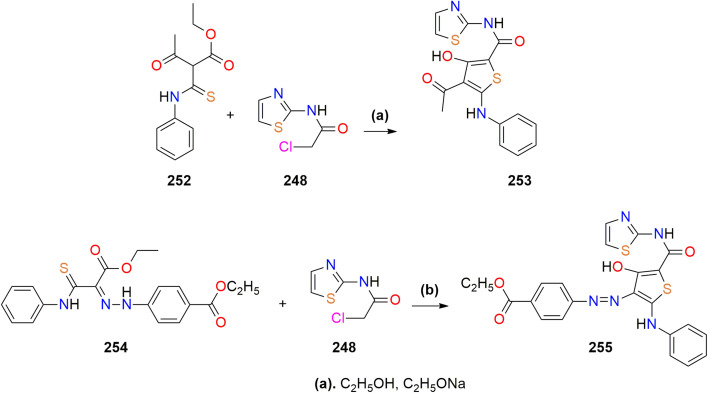
Synthesis of 3-hydroxy-5-(phenylamino)-*N*-(thiazol-2-yl)thiophene-2-carboxamides 253, 255.

**Scheme 47:**
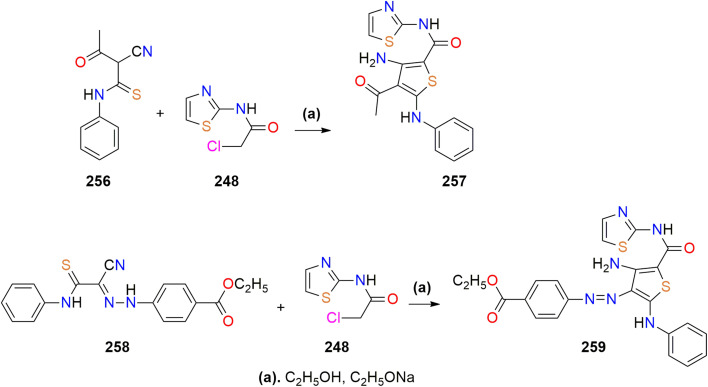
Synthesis of 3-amino-5-(phenylamino)-*N*-(thiazol-2-yl)thiophene-2-carboxamides 257–259.

H.M. Abumelha, A. Bayazeed, A. Alsoliemy *et al.* synthesized six thiophene-based thiazoles 249, 251, 253, 255, 257, 259 (Schemes 45–47). Thiocarbamoyls 247, 250, 252, 254, 256, 258, and sodium ethoxide solution in ethanol, were added 2-(chloroacetamido)thiazole 248, at reflux for 4 h. The mixture was cooled, collected, and recrystallized from ethanol to afford *N*-(thiazol-2-yl)thiophene-2-carboxamides 249, 251, 253, 255, 257, 259.^98^

Thiophene-based thiazoles 249, 251, 253, 255, 257, 259 have reasonable anti-oxidant potential in DPPH FRSA (IC_50_ = 23.17 ± 0.36–43.28 ± 0.14 µg mL^−1^), using BHT (IC_50_ = 26.33 ± 0.05 µg mL^−1^), and Ascorbic acid (IC_50_ = 23.06 ± 0.02 µg mL^−1^) as drug. 251, 255, and 259 containing arylazo-thiophene moiety were good anti-oxidants (IC_50_ = 32.26 ± 0.07, 27.31 ± 0.23, 23.17 ± 0.36 µg mL^−1^). Thiazolyl-thiophene hybrids 249, 253, and 257 containing acetyl-thiophene moiety showed reasonable anti-oxidant potential (IC_50_ = 43.28 ± 0.14, 35.36 ± 0.11, 37.52 ± 0.04 µg mL^−1^) in comparison to BHT, and Ascorbic acid. Limited SAR suggests:

• Aminothiophenes 257, 259 are potent anti-oxidants.

• Hydroxythiophenes 253, 255 showed reasonable scavenging.

• Methyl thiophenes 249, 251 showed lowest IC_50_ values than rest of arylazo-thiophene, and acetyl thiophene hybrids.

Inhibitors 259 and 255 have lower *E*_HOMO_–*E*_LUMO_ (1.64, 1.76 eV), and exhibited potent anti-oxidant effects (lower IC_50_), in reference to 249, 257 with higher *E*_HOMO_–*E*_LUMO_, having lower activity (higher IC_50_). This translates into facile intramolecular charge transfer for small HOMO–LUMO energy gap.^98^

### 2.8. Hydrazide based thiazoles

Hydrazide-based thiazoles are sophisticated hybrids, that utilize the hydrazide linker to generate highly polar, and flexible bridge between the thiazole core, and other bioactive aromatics. This specific linkage is a “hotspot” for hydrogen bonding, allowing these molecules to lock firmly into the active sites of metabolic enzymes. The presence of hydrogen-bond acceptor, and donor sites, turns hydrazides, as potent anti-oxidant, and anti-inflammatory motifs against RONS, often show potential against LIPOX enzyme, and lipid peroxidation. These hybrids effectively manage T2DM through “dual-action” approach. This includes exceptional α-glucosidase inhibition, with IC_50_ values in low micromolar range, as well as α-amylase inhibition, potent than Acarbose. Hydrazide moiety effectively prevents the formation of Advanced Glycation End-Products, which is crucial for protecting diabetic patients from long-term complications such as kidney failure and nerve damage. By mimicking structural features of certain PPAR-γ agonists, hydrazide-based thiazoles, and related thiazolidine-2,4-diones hybrids can improve how cells respond to insulin, facilitating better glucose uptake from the bloodstream.

**Scheme 48:**
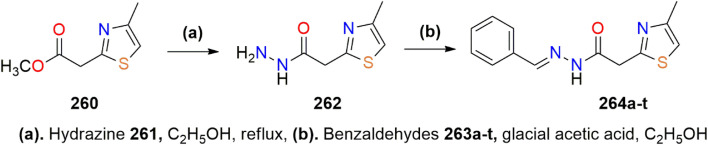
Synthesis of hydrazide-thiazole hybrids 264a–t.

M. Taha *et al.* synthesized twenty hydrazide-based thiazoles (Scheme 48). Thus, methyl 2-(4-methylthiazol-2-yl)acetate 260, and hydrazine hydrate 261, in methanol were refluxed to afford 2-(4-methylthiazol-2-yl-acetohydrazide) 262. Consequently, the obtained hydrazide 262 reacts benzaldehydes 263a–t in methanol, and acetic acid as catalyst to yield hydrazide base thiazole hybrids 264a–t.^99^

264a–t showed variable inhibitions (1.10 ± 0.10–17.20 ± 0.30 µM) against α-glucosidase compared to Acarbose (IC_50_ = 9.80 ± 0.20 µM). SARs suggest 264s (IC_50_ = 1.10 ± 0.10) with three OH groups, as the most potent. Mono hydroxyl 264f (IC_50_ = 2.50 ± 0.10 µM), 264g (IC_50_ = 1.30 ± 0.10 µM), and 264t (IC_50_ = 5.20 ± 0.10 µM), and di-hydroxyls 264n (IC_50_ = 1.60 ± 0.10 µM), 264o (IC_50_ = 3.60 ± 0.10 µM), 264p (IC_50_ = 4.20 ± 0.20), 264q (IC_50_ = 1.20 ± 0.10 µM) and 264r (IC_50_ = 3.60 ± 0.10 µM) were less potent due to decrease in number of hydroxyl groups. 264c–e displayed dynamic inhibitions due to chloro group. 264c exhibited higher inhibition (IC_50_ = 3.50 ± 0.10 µM) than 264d (IC_50_ = 5.40 ± 0.10 µM), and 264e (IC_50_ = 4.40 ± 0.10 µM). Among 264h–j (IC_50_ = 12.30 ± 0.20, 14.60 ± 0.30, 13.80 ± 0.20 µM), 264h was more potent indicating methoxy group as favorable at 2-position, and 264l as least potent (IC_50_ = 15.10 ± 0.30 µM). 264a–b (IC_50_ = 17.20 ± 0.30, 14.60 ± 0.20 µM) were the least potent due to electron withdrawing nitro goup.^99^

**Fig. 78:**
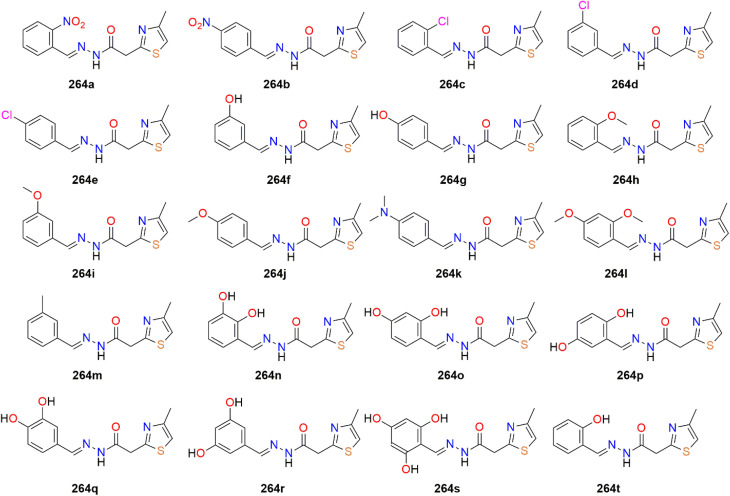
Structures of the screened hydraide-thiazole hybrids 264a–t.

Against α-amylase, varied inhibitions (0.90 ± 0.01–16.20 ± 0.30 µM) were noted compared to Acarbose (IC_50_ = 10.30 ± 0.20 µM). 264c, 264e–f, and 264n–s were superior inhibitors than drug. 264r (IC_50_ = 0.90 ± 0.01 µM) served as the most potent inhibitor due to two hydroxyl groups. Slight differences in inhibition of 264n–r (IC_50_ = 1.20 ± 0.10, 2.20 ± 0.10, 5.40 ± 0.10, 3.80 ± 0.10 µM) is due to the different position of OH group on aryl ring. 264g (IC_50_ = 1.50 ± 0.10 µM) being the 2^nd^ most potent, holds OH group at 4-position, favorable for interaction. 264f, and 264s–t (IC_50_ = 2.80 ± 0.10, 4.60 ± 0.10, 1.20 ± 0.10 µM) showed excellent inhibition. 264c–e (IC_50_ = 3.20 ± 0.10, 5.50 ± 0.10, 4.30 ± 0.10 µM) exhibited remarkable inhibition. 264a (IC_50_ = 16.20 ± 0.30 µM) displayed low inhibition due to EWG (Nitro) than 264b (IC_50_ = 13.20 ± 0.20 µM) (Fig. 78).^99^

MD analysis of the most potent inhibitors 264q, and 264s interacted with the active sites. 264q against α-amylase revealed interactions with Arg267, Asn431, and Asn481, while 264s developed similar interactions in addition to Lys268. 264q, and 264s revealed similar interactions against α-glucosidase. Based on orientation within the active site, the potent inhibitors bearing electron donating groups shared electron density to the reaction center than EWGs, and served as highly potent inhibitors of both enzymes.^99^

**Scheme 49:**
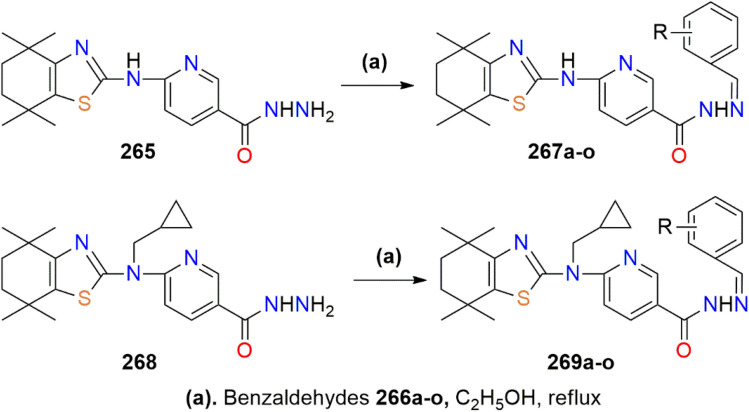
Synthesis of tetrahydro benzo[*d*]thiazole tethered nicotinohydrazides 267a–269o.

S.B. Patchipala *et al.* synthesized thirty hydrazide thiazoles 267a–2690 (Scheme 49). Thus, carboxylic acid hydrazides 265, 268 dissolved in ethanol, and benzaldehydes 266a–o were refluxed for 3 h. The mixture was cooled, filtered, and recrystallized from ethanol, to obtain the desired hydrazide-based thiazoles 267a–269o.^100^

**Fig. 79:**
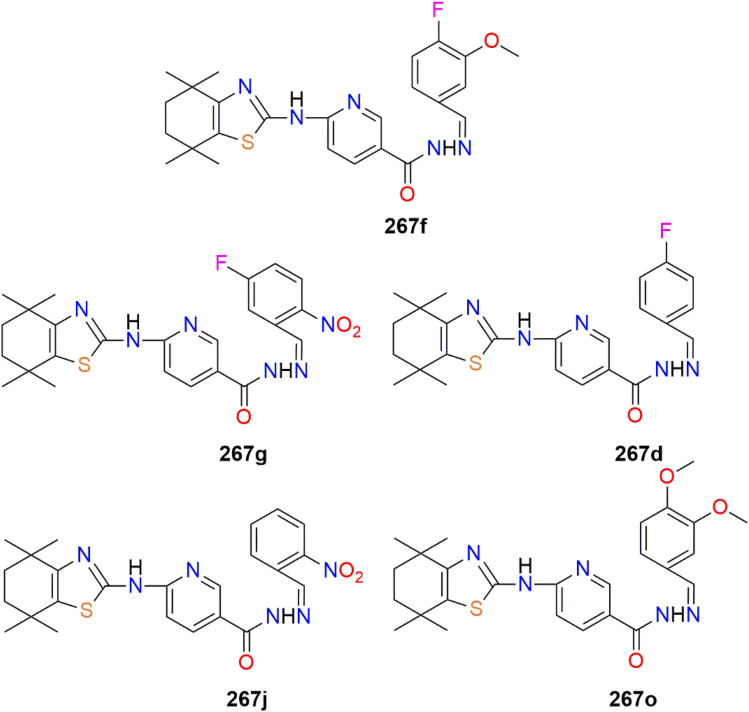
Structures of the potent tetrahydro benzo[*d*]thiazole tethered nicotinohydrazides.

Alloxan administration leads to a significant increase in blood glucose levels (295.3 ± 6.40 mg dL^−1^) when compared to normal control mice. Inhibitors reduced blood glucose level (in seven days) significantly compared to diabetic mice. 267f–g showed significant reduction in blood glucose levels (214.3 ± 2.92–118.8 ± 6.21 mg dL^−1^, 226.8 ± 5.10–115.5 ± 3.09 mg dL^−1^). 267d, 267j, and 267o showed moderate decrease in blood glucose level compared to Glibenclamide. For 28 days, change in the blood glucose levels was clear, and the raised blood glucose levels were normalized (Fig. 79).^100^

**Scheme 50:**
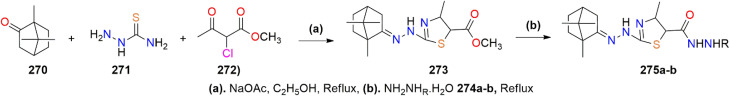
Synthesis of camphor thiazoles 275a–b.

H. Cahyana, G. Santika and K. Phukan synthesized three camphor-based thiazoles 273, 275a–b (Scheme 50). Thus, camphor 270, ethyl-2-chloro acetoacetate 272, and thiosemicarbazide 271 dissolved in ethanol, were mixed with sodium acetate catalyst. The reaction mixture was refluxed for 10 h, cooled, filtered, and recrystallized from ethanol 273. Later, camphor thiazole 273, and hydrazine or phenyl hydrazine 274a–b, dissolved in ethanol were refluxed for 4 h at 70 °C. Upon completion, the mixture was evaporated, dried, and recrystallized from ethanol to get the required products 275a–b.^101^

**Fig. 80:**
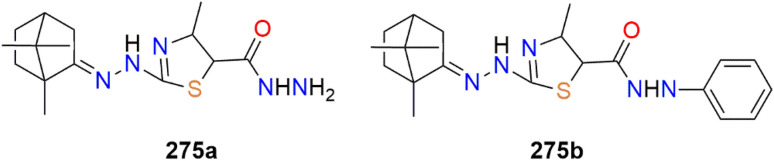
Structures of the potent camphor thiazoles.

273, 275a–b were screened against α-glucosidase enzyme compared to Acarbose (IC_50_ = 0.33 ppm). Thus, the inhibitory potential increased with increase in the concentration of the inhibitors. In terms of their IC_50_ values, camphor thiazole 273 showed enhanced inhibition (IC_50_ = 859.06 ppm) than camphor thiazole hydrazine, and phenylhydrazine 275a–b (IC_50_ = 1893.40, >2000 ppm). Thus, hydrazine group does not increase inhibition of α-glucosidase enzyme (Fig. 80).^101^

273, 275a–b were evaluated in DPPH FRSA. Thus, the results obtained suggest modification of camphor with thiazole hydrazine/phenylhydrazine 275a–b favored anti-oxidant effects against DPPH free radicals, and can serve as potential anti-oxidants, alhough IC_50_ values of these inhibitors have not been able to surpass Vitamin C (IC_50_ = 4.72 ppm).^101^

### 2.9. Indole-based thiazoles

Indole-based thiazoles are advanced molecular hybrids that combine two of the most significant “privileged” scaffolds. By fusing indole nucleus (common in natural products like serotonin) with the thiazole ring, the obtained hybrids manage diabetes through superior enzyme inhibition, and antiglycation properties. Indole-based thiazoles address metabolic failures of T2DM through multiple pathways. These hybrids inhibit α-glucosidase, and α-amylase effectively, and suppress postprandial hyperglycemia comparable to Acarbose. These hybrids are highly effective at preventing the formation of Advanced Glycation End-products to help mitigate late-stage diabetic complications such as neuropathy and retinopathy.

**Scheme 51:**
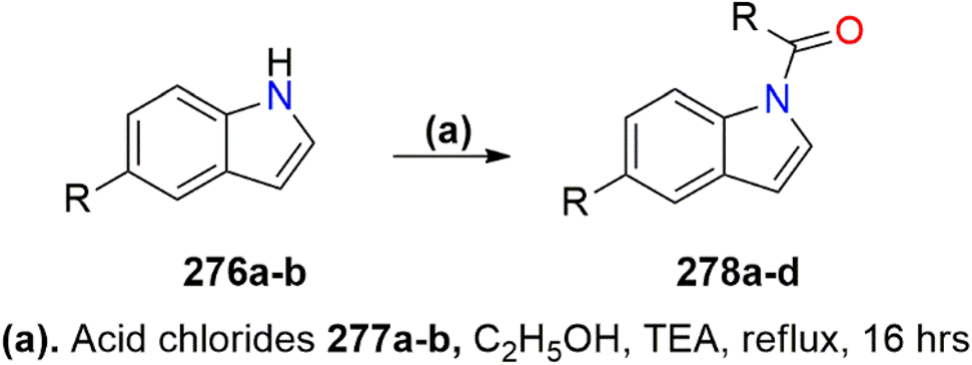
Synthesis of indole-based thiazoles 278a–d.

S. Jagadeesan and S. Karpagam *et al.* synthesized four indole-based thiazole hybrids (Scheme 51). Indole 276a–b dissolved in dry DMF was cooled to 0 °C, adding 60% NaH in mineral oil, and stirred the mixture for 30 min at 0 °C. Acid chlorides 277a–b were added gently, and stirred for 60 min at 0–5 °C. Later, ice-cold water was added, extracted twice with DCM, washed with de-ionized water, and dried over anhydrous sodium sulphate. The salt was filtered off, and the solvent was evaporated to give the crude product, purified by silica gel column chromatography (60–100 mesh) using hexanes/ethyl acetate to get 278a–d.^102^

**Fig. 81:**
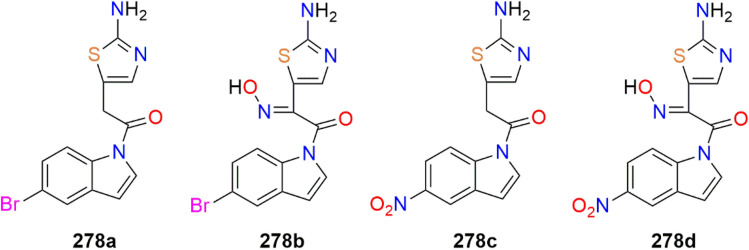
Structures of indole-based thiazoles 278a–d.

278a–d were assessed against *in vitro* DPPH FRSA. 278a–d showed sensible anti-oxidant potential, with 278a displayed higher inhibition than Ascorbic acid (EC_50_ = 17.95 µg mL^−1^), due to bromine on the indole ring, and heterocyclic *N*-acyl side chain at the 1^st^ position, followed by 278b, where low inhibition was observed. Among the nitro derivatives, 278d exhibited higher anti-oxidant effects (EC_50_ = 13.2 ± 1.2 µg mL^−1^). Thus, nitro group at 5^th^ position of indole ring halts anti-oxidant effects, while bromo group at 5^th^ position shifts electrons over the ring system to quench free radicals by creating a metastable indolyl cation radical. To conclude, bromo indole hybrid, and the heterocyclic side chain attached to the first position gives conclusive anti-oxidant effects (Fig. 81).^102^

### 2.10. Cholic acid-thiadiazole hybrids

Cholic acid-thiadiazole hybrids are specialized antidiabetic candidates that combine the natural metabolic regulatory properties of bile acids with the synthetic enzyme-inhibiting power of the 1,3,4-thiadiazole ring. This hybridization strategy targets both the absorption of sugars in the gut, and the signaling pathways that control systemic glucose homeostasis. Thiadiazole moiety possess inhibitory effects carbohydrate-digesting α-glucosidase, and α-amylase enzymes more potent than Acarbose. Cholic acid is a natural ligand for receptors like TGR5 and FXR. By tethering Cholic acid to thiadiazole, activate these receptors, which stimulates GLP-1 secretion from intestinal L-cells and enhances insulin sensitivity in peripheral tissues.

**Scheme 52:**
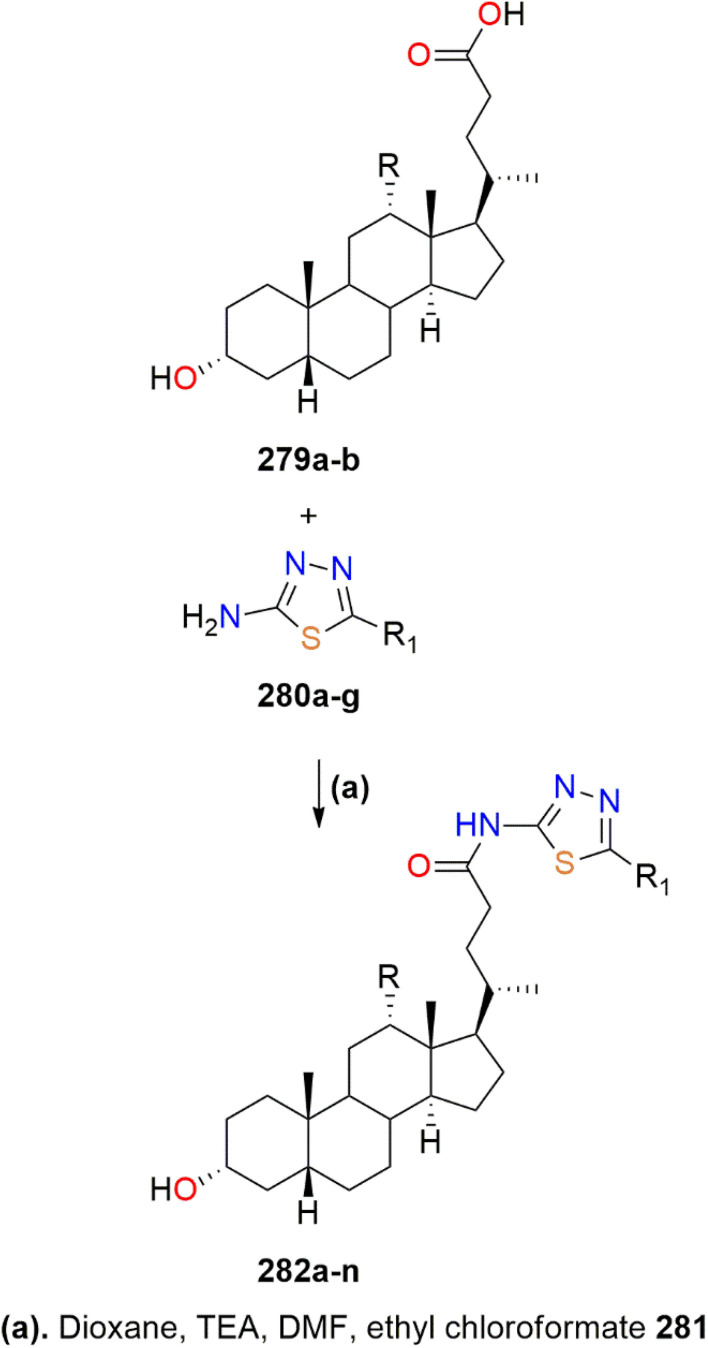
Synthesis deoxycholic/lithocholic acid-thiadiazoles 282a–n.

S. Patel and S. Mishra *et al.* synthesized fourteen deoxycholic/lithocholic acid-thiadiazole conjugates 282a–n(Scheme 52). Thus, deoxycholic and lithocholic acids 279a–b were activated by anhydride form, reacting with ethyl chloroformate 281 in 1,4-dioxane, and DMF solutions at 0 °C for 20 min. Then, TEA, and a solution of substituted 2-amino thiadiazole 280a–g in DMF were added at stirring until completion. Later, upon purification by silica gel chromatography with ethyl acetate/hexane, the crude product afforded the deoxycholic acid-thiadiazole conjugates 282a–n.^103^

**Fig. 82:**
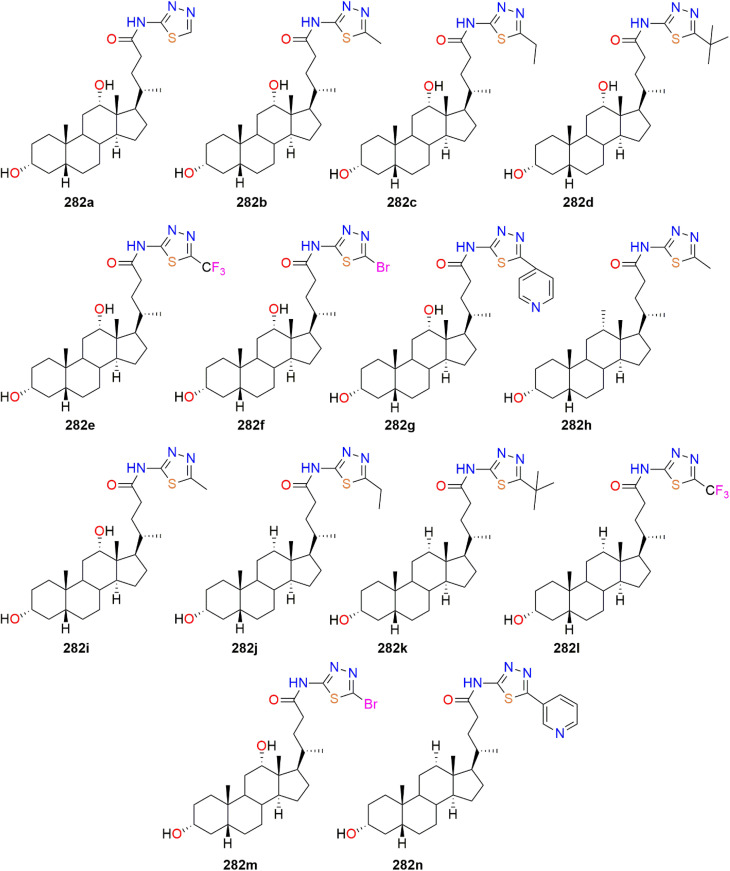
Structures of the deoxycholic/lithocholic acid-thiadiazole hybrids 282a–m.

DPPH FRSA evaluated 1,3,4-thiadiazole 280, and conjugates 282a–n, using Ascorbic acid (IC_50_ = 20.72 ± 1.02 µM) as drug. Thus, thiadiazole-deoxycholic/lithocholic acid conjugates 282b, 282c, 282d, 282e, 282h, 282i, 282j, and 282n exhibited good DPPH FRSA (IC_50_ = 13.45 ± 0.25–19.23 ± 0.23 µM) serving as electron donors. Most of the conjugates showed better DPPH FRSA. 282c, and 282j showed better DPPH, and ABTS inhibitions (IC_50_ = 15.34 ± 0.07, 13.45 ± 0.25 µM), (IC_50_ = 69.65 ± 1.23, 67.59 ± 1.47 µM). 282c showed higher inhibition of ABTS (IC_50_ = 69.65 ± 1.23 µM). 282j showed strongest DPPH FRSA (IC_50_ = 13.45 ± 0.25 µM). Anti-oxidant potential enhanced significantly by introducing ethyl group at 5^th^ position. Thiadiazole, and conjugates served as relatively better anti-oxidants. Thiadiazole, and deoxycholic acid conjugates anti-oxidant activity enhanced by substitution at 5^th^ position with EDGs. However, 282e potential decreased sharply by changing 5^th^ position to EWG (CF_3_). In ABTS assay, 282c, and 282j were superior anti-oxidants, though the poor solubility negatively affected anti-oxidant potential (Fig. 82).^103^

### 2.11. Oxadiazole-thiadiazole hybrids

Oxadiazole-thiadiazole hybrids are emerging multitarget heterocycles, that integrate oxygen based oxadiazole, and sulfur based 1,3,4-thidiaole rings into single molecular framework. This combination enhances their antidiabetic efficacy by allowing simultaneous interaction with several enzymatic targets responsible for glucose homeostasis. These dual-action inhibitors primarily inhibit digestive systems, and oxidative stress. These highly potent α-glucosidase, and α-amylase inhibitors delay carbohydrate digestion outperforming Acarbose. By blocking these enzymes, the hybrids induce a slower release of glucose into the bloodstream, preventing the sharp blood sugar spikes common after meals. The thiadiazole unit provides strong radical scavenging abilities, vital for protecting pancreatic β-cells from oxidative stress.

**Scheme 53:**
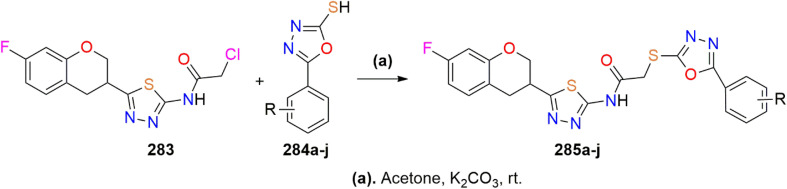
Synthesis of thiadiazole hybrids 285a–j.

M. Rupapara *et al.* synthesized ten oxadiazole-thiadiazole hybrids 285a–j (Scheme 53). Thus, 5-aryl-1,3,4-oxadiazol-2-thiols 284a–j dissolved in acetone, and potassium carbonate were stirred for 30 min. Then, *N*-acetylated 1,3,4-thiadiazole 283 was added, stirred for 6 h, and poured into cold water, washed, and dried to get target hybrids 285a–j.^104^

**Fig. 83:**
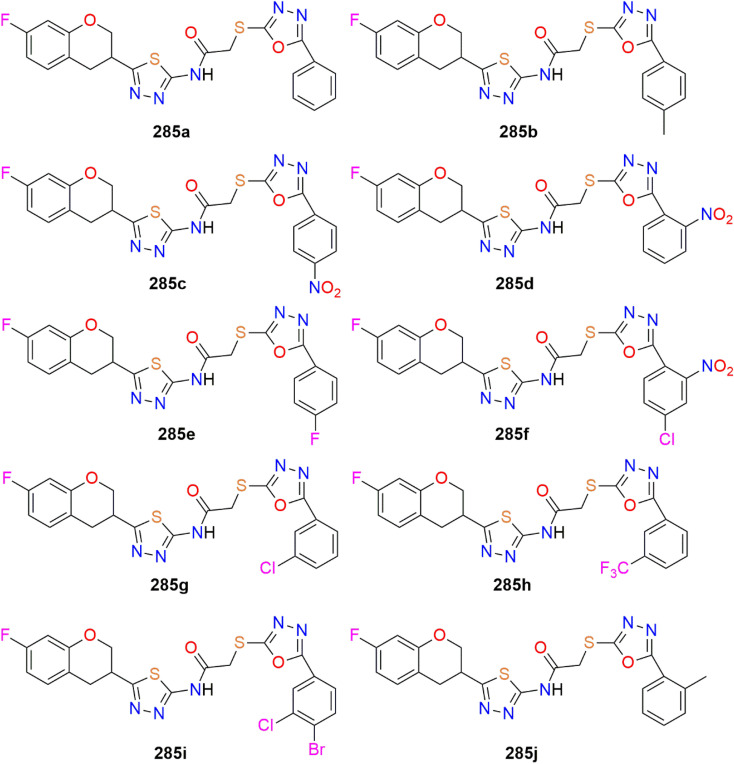
Structures of the potent thiazole hybrids 285a–j.

285a–j were screened for DPPH FRSA, using Ascorbic acid as drug (781.32 ± 10.07 µM). 285a–j showed significant DPPH scavenging (IC_50_ = 399.57 ± 2.33–638.68 ± 15.93 µM). 285c exhibited highest (IC_50_ = 399.57 ± 2.33 µM), and 285d as promising inhibitions (IC_50_ = 551.85 ± 8.06 µM). Inhibitions decreased (IC_50_ ≥ 600 µM) with EDGs 285a, 285b, 285j than oxadiazole moiety bearing fluorine (285e = 565.71 ± 9.29, 285h = 574.48 ± 15.04 µM). Chlorine 7285, 285g, bromine 285i, phenyl 285a, and methyl group 285j, 285e decreased scavenging (>600 µM). General trend observed was: Nitro > Fluoro > Chloro > H > Methyl > Bromo (Fig. 83).^104^

### 2.12. Coumarin–thiadiazole hybrids

Coumarin–thiadiazole hybrids have emerged as promising multifunctional antidiabetic scaffolds, combining the well-recognized pharmacological potential of coumarins with the metabolic, and enzyme-binding versatility of the 1,3,4-thiadiazole ring. Coumarins are known for their antioxidant, and enzyme-modulating properties, while thiadiazoles contribute enhanced stability, electron-rich heteroatoms, and favorable interactions with biological targets. Their hybridization offers a rational strategy for developing compounds with improved glycemic control. These hybrids primarily exert antidiabetic effects through inhibition of carbohydrate-digesting enzymes, particularly α-glucosidase and α-amylase, leading to delayed glucose absorption, and reduced postprandial hyperglycemia. In addition, the strong anti-oxidant potential of coumarin–thiadiazole conjugates helps mitigate oxidative stress, a key factor in the progression of diabetes, and its associated complications. Structural diversity within this hybrid framework enables fine-tuning of activity, selectivity, and safety. Overall, antidiabetic coumarin–thiadiazole hybrids represent a valuable platform for the development of next-generation glucose-lowering agents with dual enzyme-inhibitory, and protective antioxidant effects, supporting their continued exploration in medicinal chemistry, and drug design.

**Scheme 54:**
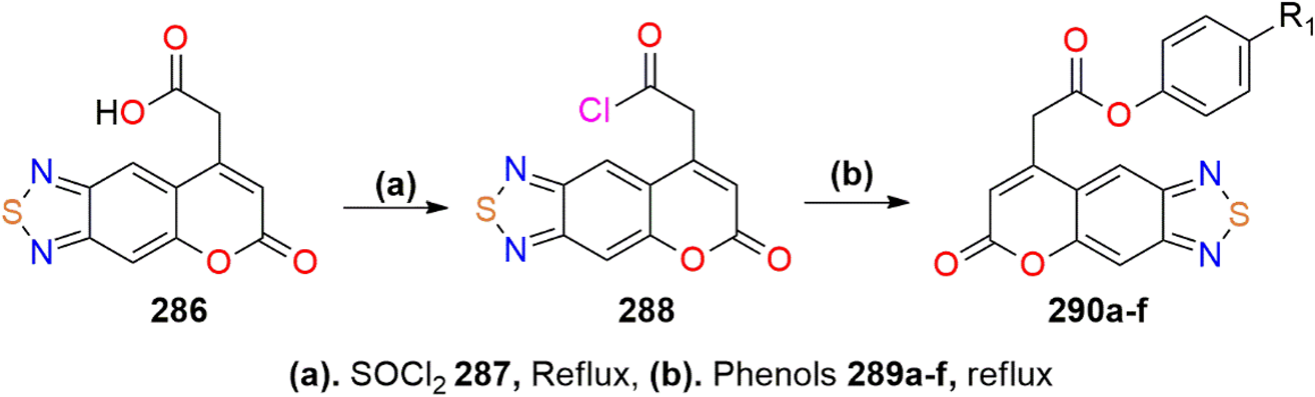
Synthesis of linearly fused thiadiazolocoumarins 290a–f.

R. N. Jibroo *et al.* synthesized six linearly fused thiadiazolocoumarins (Scheme 54). Precursor 286, and thionyl chloride 287, were stirred for 30 min in anhydrous conditions, followed by reflux, for three hr at room temperature. Upon completion, thionyl chloride was distilled, to get acetyl chloride 288. Solution of azabenzene, and 4-substituted phenols 289a–f in dehydrated diethyl ether, and precipitates were stirred for 30 min, and then refluxed. Upon completion, water was added, and organic layer was vaporized to 290a–f.^105^

**Fig. 84:**
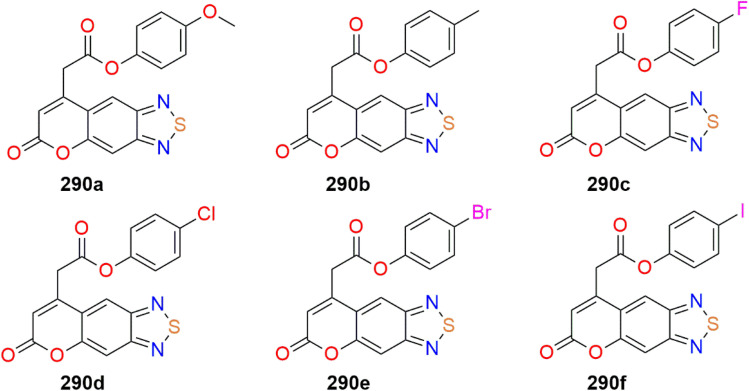
Structures of linearly fused thiadiazolocoumarins.

290a–f were evaluated against oxidative stress on a human neuroblastoma cell line (SH-SY5Y, ATCC: CRL-2266) treated with H_2_O_2_ to induce oxidative stress in the cells. H_2_O_2_, DMF, and DMF in combination with H_2_O_2_ served as negative control, positive control, and reference. 290c–d showed strong anti-oxidative stress activity (214.37 ± 0.75, 215.76 ± 1.02) close to the reference (211.77 ± 0.88). Anti-oxidative stress effectiveness was ordered in a decreasing manner as follows: 290c, 290d, 290a, 290b, 290e, and 290f (Fig. 84).^105^290a–f were evaluated for antidiabetic effects against porcine α-amylase, and yeast α-glucosidase, using Acarbose as drug. In terms of their IC_50_ values, the moderate inhibition ranges from 326.67–372.89 µg mL^−1^, and 364.73–424.54 µg mL^−1^ against α-amylase, and α-glucosidase enzymes in comparison to drug (263.98 ± 0.86, 283.78 ± 0.84 µg mL^−1^). Inhibitions decreased as: 290a, 290b, 290c, 290d, 290e, and 290f against both enzymes. 290a–b were found to be more potent, owing to the presence of EDGs (Methyl, Methoxy) at C-4′. In 290c–d, enzymes inhibitory effects decreased due to the presence of EWGs (F, Cl, Br, I) at position 4′.^105^

### 2.13. Thiophene–thiadiazole hybrids

Thiadiazoles are significant anti-inflammatory, anti-oxidant, and antidiabetic heterocycles. Thiadiazoles potentially scavenge RONS, and key carbohydrate-hydrolyzing enzymes (α-amylase, α-glucosidase) which helps suppress postprandial hyperglycemia. Thiophene is a sulfur-based five-membered stable, and versatile scaffold heterocycle, which effectively scavenge RONS, alleviating oxidative stress, recognized as crucial in the progression of diabetes, and its associated complications. Thiophene-conjugated heterocycles, such as thiazole-pyrazoline hybrids, possess pronounced anti-oxidant effects through superoxide radical suppression, and efficient DPPH free radical scavenging, with efficacy comparable to standard antioxidants Allopurinol, and Ascorbic acid. Collectively, the integration of thiophene, and 1,3,4-thiadiazole frameworks offers a synergistic effect by combining anti-oxidant activity with subsequent α-amylase, and α-glucosidase inhibition. This dual functionality supports the rational design of novel antidiabetic agents. and highlights the therapeutic promise of thiophene-thiadiazole hybrids in managing diabetes, and oxidative stress-related complications.

**Scheme 55:**
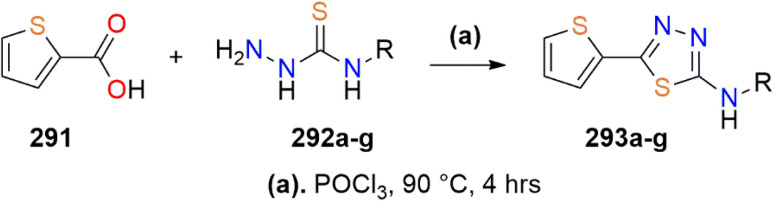
Synthesis of thiophene based 1,3,4-thiadiazole hybrids 293a–g.

H. Mŭglu *et al.* synthesized seven 1,3,4-thiadiazoles 293a–g (Scheme 55). Thus, 2-thiophenecarboxylic acid 291, and thiosemicarbazone 292a–g were added phosphorous oxychloride at 90 °C for 4 h. Upon completion, cold water was added into mixture, neutralized by ammonia, and filtered.^106^

**Fig. 85:**
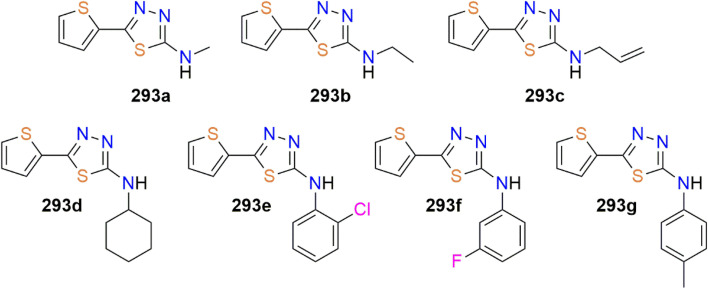
Structures of 1,3,4-thiadiazoles 293a–g.

293a–g scavenged DPPH free radicals compared to Trolox as drug. At 512 µg mL^−1^, 293a served as most potent anti-oxidant (81.13%), followed by 293b (71.36%). DPPH free radical scavenging increased with concentration, with highest free radical scavenging showed by 293a (81.13%), and 293d as the least potent (9.11%). DPPH free radical scavenging decreased as: 293a > 293f > 293e > 293 > 293c > 293b > 293d. Highest anti-oxidant effects were observed for 293a. F, Cl, and CH_3_ groups changed anti-oxidant effects as: (F > Cl > CH_3_) (Fig. 85).^106^

### 2.14. Spiro pyrrolo-thiazoles

Spiro pyrrolo-thiazoles represent a class of structurally sophisticated heterocycles in which two or more cyclic systems are connected through a shared quaternary spiro center. This unique architecture confers pronounced three-dimensionality, conformational rigidity, physicochemical stability, and axial chirality. Such features enable efficient interaction with biological targets, as the rigid 3D geometry enhances binding selectivity while reducing conformational flexibility, an advantage for designing safer, and more potent therapeutic agents.

In diabetes management, these compounds demonstrate notable activity through the inhibition of key carbohydrate-hydrolyzing enzymes, specifically α-amylase, and α-glucosidase. These enzymes catalyze the breakdown of complex carbohydrates into absorbable glucose units. Their inhibition delays carbohydrate digestion and glucose absorption, thereby helping to regulate postprandial hyperglycemia. Several synthesized derivatives, and hybrid analogues exhibit inhibitory effects comparable to established drugs such as acarbose, highlighting their therapeutic promise. Beyond enzyme inhibition, spiro pyrrolo-thiazoles also display significant antioxidant potential. By scavenging reactive oxygen, and nitrogen species, they mitigate oxidative stress, considered as a major contributor to the onset, and progression of diabetes and its complications. This anti-oxidant activity supports cellular protection, particularly in pancreatic β-cells, and other metabolically active tissues, and may help prevent long-term diabetic complications. Overall, the multifunctional profile of spiro pyrrolo-thiazoles, including enzyme inhibition, glycemic control, and anti-oxidant activity positions them as promising lead compounds for the development of next-generation antidiabetic therapies.

**Scheme 56:**
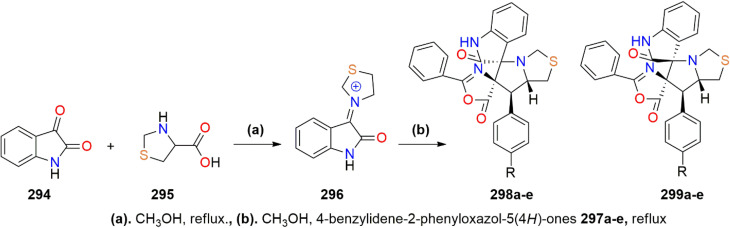
Synthesis of dispiropyrrolothiazoles 298a–299e.

**Scheme 57:**
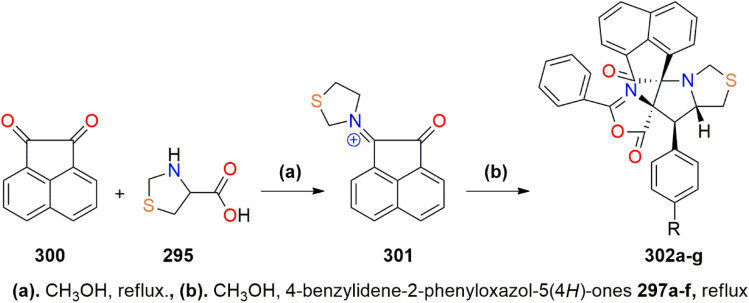
Synthesis of dispiropyrrolothiazoles 302a–g.

M. B. Hammouda, S. Boudriga, K. Hamden *et al.* synthesized sixteen sulfonate esters of 2-(2-benzylidenehydrazono)thiazolidin-4-one (Schemes 56 and 57). Thus, 4-benzylidene-2-phenyloxazol-5(4*H*)-ones 297a–e, L-4-thiazolidine carboxylic acid 295, and isatin 294 were refluxed in methanol for 4 h. Once completed, water was added to the mixture, filtered, and chromatographed on silica gel using ethyl acetate-cyclohexane (3 : 7 v/v) as eluent to get pure 298a–e, and 299a–e. Reaction mixture of 4-benzylidene-2-phenyloxazol-5(4*H*)-ones 297a–f, L-4-thiazolidine carboxylic acid 295, and acenaphthenequinone 300 was refluxed in methanol for 4 h. Once completed, water was added, product was filtered, and purified using silica gel flash chromatography yielding the target dispiropyrrolothiazoles 302a–f.^107^

**Fig. 86:**
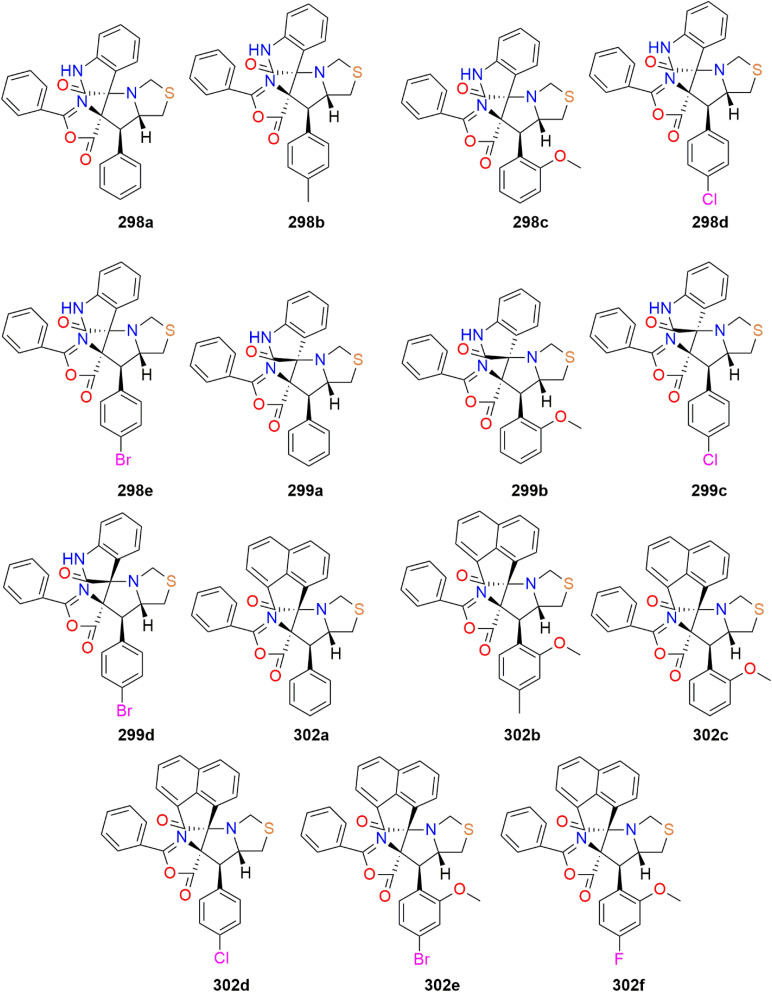
Structures of the synthesized dispiropyrrolothiazoles.

Antihyperglycemic assay assessed antidiabetic potential of 298a–e, 299a, c–e, and 302a–f using Acarbose (IC_50_=(α-amylase) = 1.28 ± 0.13, (α-glucosidase) = 2.80 ± 0.20 Mm). Thus, some inhibitors showed moderate to potent effects. Among 298a–e, 298b served as the most potent (IC_50_ = 1.76 ± 0.14 µM), among 299a–e, 299d (IC_50_ = 2.88 ± 0.19 µM), and among 302a–e, 302f (IC_50_ = 2.18 ± 0.17 µM) served as the most potent inhibitor against α-amylase. Similarly, 298b (IC_50_ = 4.81 ± 0.24 µM) in 298a–e, 299d (IC_50_ = 7.78 ± 0.37 µM) in 299a, 299c–e, and 302e (IC_50_ = 6.41 ± 0.25 µM) in 302a–f served as the most potent inhibitors against α-glucosidase. SARs suggest inhibitors 298a–e, with EDGs like methyl at *para-*position 298b or methoxy at *ortho*-position 298c enhanced inhibitions than chloro 298d, and bromo 298e. 299d showed greater inhibition over 298d due to difference in stereochemistry of the pyrrolidine. 302a–f with acenaphthylen-1(2*H*)-one, increased inhibition of 302c–f than 302a–b due to the formation of more hydrogen bonds (Fig. 86).^107^

Molecular docking analyzed binding of the inhibitors with amino acid residues. Ligand-protein complexes formed within the binding sites showed negative energies. Minor differences were observed (0.5 kcal mol^−1^) in the binding energies of 302a–f. Lower inhibition of 302a–b compared to 302c–f suggests fewer hydrogen bonds were formed with the interacting amino acids. 298d, and 299d differ by stereochemistry at the stereocenter located on their pyrrolidine rings. 299d provided superior inhibition compared to 298d, which aligns with predictions from docking studies. Active site of α-amylase enzyme is characterized by three essential amino acids: Glu233, Asp300, and Asp197. 299d binds through hydrogen bonding to the α-amylase active site (Glu233, Asp300), 298d has single interaction (Asp300), allowing 299d as better inhibitor. 299d-α-amylase was stabilized by Trp59, His101, Leu162, and Leu165. 298d demonstrated increased π-contacts through shared interactions with residues Tyr62, Leu165, Ala198, and Gly233.^107^

### 2.15. Simple thiazoles

**Fig. 87:**
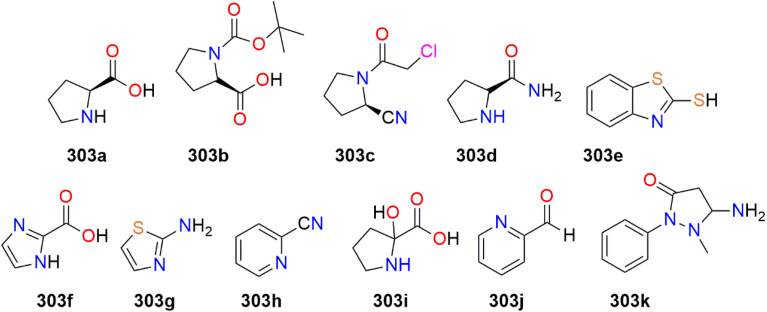
Structures of the screened pyrrolidine, and thiazole heterocycles 303a–k.

A. A. Bhat *et al.* analysed antidiabetic potential (DPP-IV, α-glucosidase, and α-amylase) of differently substituted eleven pyrrolidine, and thiazole heterocycles 303a–k. In DPP-IV assay, 303a, and 303d (pyrrolidine rings), showed highest efficacy (IC_50_ = 26.37 ± 0.45, 23.07 ± 0.31 µg mL^−1^), and then 303g–i (IC_50_ = 26.99 ± 0.87, 26.61 ± 0.68, 26.81 ± 0.27 µg mL^−1^) compared to Sitagliptin as drug (IC_50_ = 7.6 ± 0.90 µg mL^−1^). 303e–f showed least potency (IC_50_ = 45.29 ± 0.26, 45.47 ± 0.33 µg mL^−1^) than Sitagliptin as drug. 303b displayed moderate inhibition (IC_50_ = 27.34 ± 0.62 µg mL^−1^). In α-glucosidase inhibition assay, 303g and 303a served as the most potent inhibitors (IC_50_ = 16.72 ± 0.27, 18.70 ± 0.83 µg mL^−1^), in reference to Acarbose (IC_50_ = 10.07 ± 0.81 µg mL^−1^), following 303d, 303i, and 303h (IC_50_ = 15.40 ± 0.35, 25.45 ± 0.67, 31.32 ± 0.88 µg mL^−1^). 303e, 303f, and 303c were the least effective inhibitors (IC_50_ = 48.83 ± 0.46, 41.90 ± 0.33, 41.44 ± 1.01 µg mL^−1^). In α-amylase inhibition assay, 303a, 303d, and 303g (IC_50_ = 14.64 ± 0.72, 10.17 ± 0.89, 13.67 ± 0.56 µg mL^−1^) showed comparatively higher inhibitions, augmenting their role as effective inhibitors compared to reference Acarbose (IC_50_ = 7.76 ± 0.77 µg mL^−1^). 303k demonstrated lowest inhibition (IC_50_ = 25.49 ± 1.02 µg mL^−1^). 303d emerged as the most potent inhibitor in α-amylase, DPP-IV, and α-glucosidase inhibition assays, followed by 303g, and 303a. 303h, and 303i inhibited DPP-IV significantly. Only few inhibitors served as potential α-amylase inhibitors (Fig. 87).^108^ SARs suggests 303a, and 303d as highly effective DPP-IV inhibitors, in reference to Sitagliptin. Carboxylic group in 303a provided efficacy over amide group in 303d. Sulfur, and nitrogen of thiazole increased inhibition. Proline ring, coupled with OH group on same carbon as acid 303i, improved inhibition. 303g showed highest inhibition against α-glucosidase, than 303a. Six-membered ring 303e proved unfavorable against DPP-IV, and α-glucosidase enzyme. Conversely, six-membered pyridine ring bearing cyano group on 2^nd^ position enhanced inhibition in 303h. Bulky pyrrolidine ring diminished antidiabetic effects. In α-amylase assay, 303g, and 303d showed strongest inhibition indicating hetero-atoms sulfur, and nitrogen in pyrrolidine, and amino group enhances inhibition. Replacing amide functionality of 303g with an acid in 303a lowered α-amylase inhibition. Six-membered ring with electron withdrawing cyano group 303h showed significant inhibition. 303b–c, 303e–f, and 303i–k showed moderate to lower inhibition. Thus, the inhibitors possess lower α-amylase inhibition than Acarbose.^108^

MD analyzed binding energies, and possible binding sites of 303d, 303g, using Sitagliptin (DPP-IV), and Acarbose (α-glucosidase, α-amylase) as drugs. Thus, 303d, and 303g received respective minimum binding energy for DPP-IV (−4.7, −4.2 kcal mol^−1^), α-glucosidase (−5.2, −3.9 kcal mol^−1^), and α-amylase (−4.6, −3.5 kcal mol^−1^), lower than Acarbose, and Sitagliptin. 303d revealed strong hydrogen binding with Thr350 (2.03 Å), and Thr351 (2.24 Å), Asp588 (2.78, 2.88 Å), and Met348 (2.92 Å) amino acid residues of chain A of DPP-IV enzyme. 303d displayed hydrogen bonding with His351 (1.89 Å), Asp352 (2.64 Å), Asp69 (2.22 Å), and Glu277 (2.58, 2.64 Å) amino acid residues of chain A of α-glucosidase. 303d displayed hydrogen bonding with His299 (1.94 Å), Glu233 (2.82, 2.89, 3.08 Å), and Asp300 (2.22 Å) amino acid residues of chain A of human pancreatic α-amylase. 303g revealed hydrogen bonding with Arg125 (2.95 Å), Asn710 (2.19 Å), Asn709 (2.37 Å), and Glu205 (2.44 Å) amino acid residues as well as hydrophobic interaction with Arg125 amino acid residue of DPP-IV enzyme. 303g with α-glucosidase showed hydrogen bonding with Lys156 (2.60, 2.63 Å), Glu422 (2.00 Å), and Ser236 (1.97 Å) amino acid residues. Electrostatic interaction is His423 amino acid, and hydrophobic interaction are Phe314 amino acid residues of chain A of α-glucosidase. 303g showed hydrogen binding with Arg421 (2.73), Arg398 (2.44 Å) amino acid residues. Electrostatic interactions are Arg398, and Asp402 amino acid residues, as well as hydrophobic interactions with Arg398 amino acid residue of chain A of human pancreatic α-amylase. 303a–k upon *in vitro,* and *in silico* screening suggests 303d (pyrrolidine-2-carboxamide), and 303g (thiazol-2-amine) emerged as the most potent with significant biocompatibility. 303d, and 303g designed new, and potent antidiabetic hybrid molecules 303l–t. MD analysis of 303l–t showed improved binding scores with DPP-IV (−5.6 to −6.6 kcal mol^−1^), α-glucosidase (−6.3 to −7.3 kcal mol^−1^), and α-amylase (−5.2 to −6.4 kcal mol^−1^), though lower than Sitagliptin, and Acarbose. Thus, 303l–t still lack notable diabetic efficacy.^108^

**Fig. 88:**
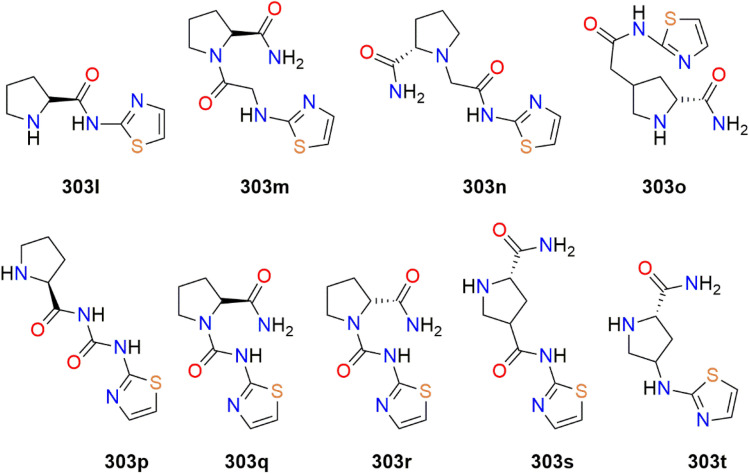
Structures of the designed pyrrolidine, and thiazole hybrid 303l–t.

**Fig. 89:**
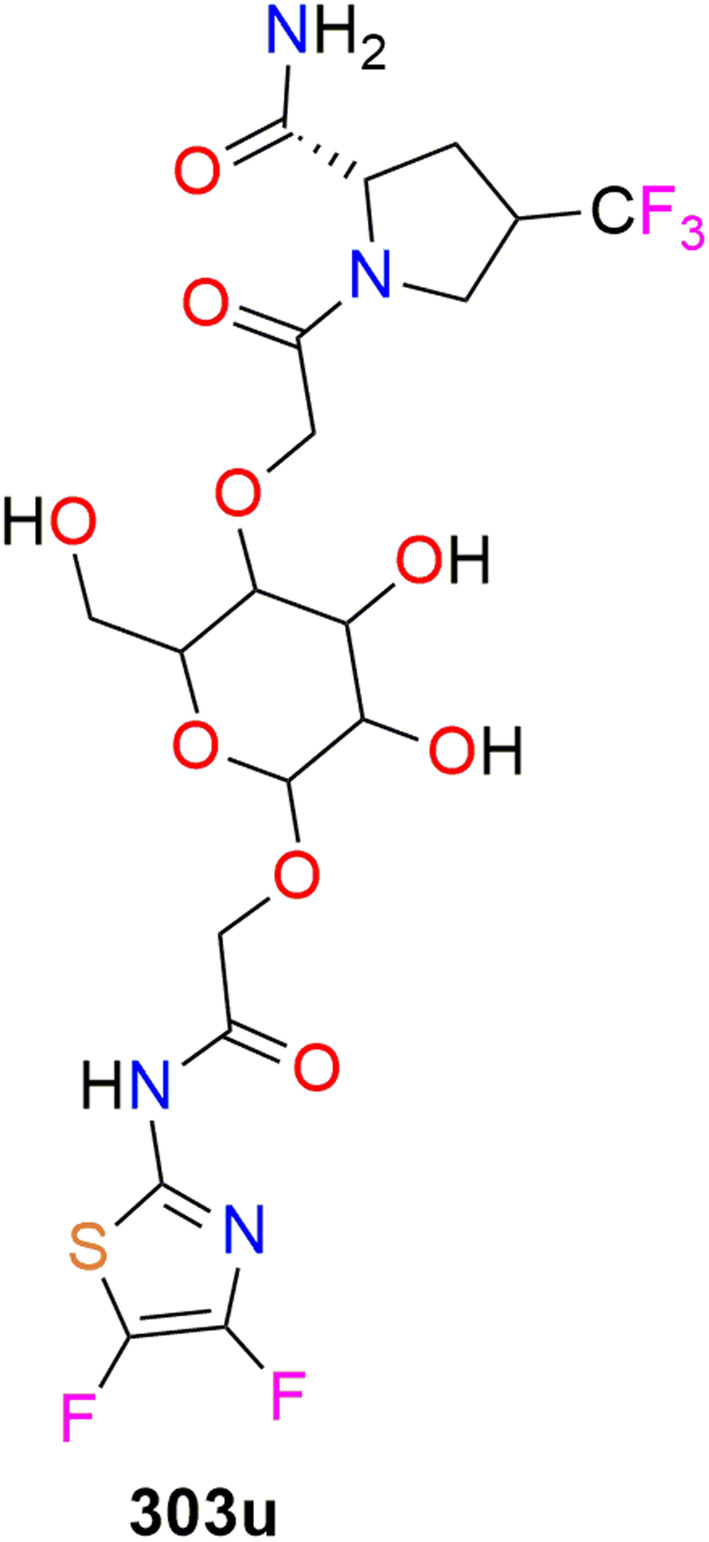
Structure of the potent thiazole containing pyrrolidine, fluorine, and carbohydrate 303u.

In search of potent antidiabetics, novel hybrid 303u was designed using pyrrolidine, thiazole, fluorine (Sitagliptin), and carbohydrate (Acarbose) moieties. 303u showed highest binding score against DPP-IV, α-glucosidase, and α-amylase (−8.8, −10.2, −8.5 kcal mol^−1^) than Sitagliptin, and Acarbose. In 303u, oxygen, nitrogen, and fluorine atoms developed hydrogen bonding with Arg125 (1.85 Å), Val546 (2.25 Å), Tyr547 (2.66 Å), Tyr662 (2.81 Å), Asn562 (2.73 Å), Asn710 (2.73 Å), Trp629 (2.70 Å), His740 (2.66, 3.04 Å), and hydrophobic interactions with Trp627 of DPP-IV enzyme. In 303u, oxygen, nitrogen, and fluorine atoms involved in hydrogen bonding with Asp69 (3.11 Å), Asp215 (2.96, 3.22 Å), Asp242 (3.26, 3.38 Å), and Asp352 (3.32 Å), Tyr158 (3.34 Å), Glu277 (2.26 Å), Glu411 (3.32 Å), Leu313 (2.04 Å), Phe314 (3.78 Å), and Arg442 (2.75 Å). Electrostatic interaction involved Asp352, and hydrophobic interactions Lys156, Tyr158, Val216, and Phe178 of α-glucosidase enzyme. With α-amylase enzyme, 303u showed hydrogen bonding with Arg252 (2.76 Å), Arg398 (2.07, 2.47 Å), Trp280 (3.33 Å), Gly281 (3.60 Å), Gly403 (2.39 Å), Glu282 (3.33 Å), Ser289 (2.61 Å), Pro332 (3.58 Å), Asp402 (2.04 Å), and Phe406 (2.20 Å). 303u shared hydrophobic interactions with His33, Pro332, and Phe335 of α-amylase enzyme (Fig. 88 and Fig. 89).^108^

**Scheme 58:**
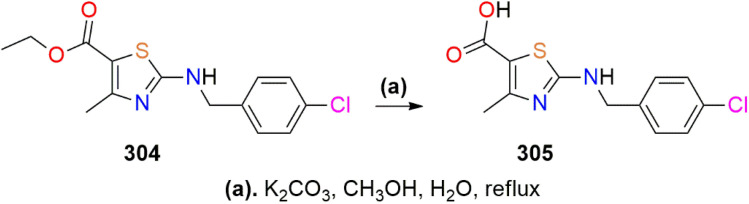
Synthesis of 2-((4-chlorobenzyl)amino)-4-methylthiazole-5-carboxylic acid 305.

Y. N. Paudel *et al.* performed convenient synthesis of anti-oxidant, anti-inflammatory, and antidiabetic 2-((4-chlorobenzyl)amino)-4-methylthiazole-5-carboxylic acid 305. Thus, ethyl 2-((4-chlorobenzyl)amino)-4-methylthiazole-5-carboxylate 304, and potassium carbonate were added into a mixture of methanol, and water (9 : 1). TLC monitored the reaction's progress, in a solvent system of benzene: acetone (4 : 1). Upon completion at reflux, the solution was cooled, neutralized with glacial acetic acid, stirred for 60 min, and recrystallized from ethanol to get the target thiazole compound 305 (Scheme 58).^109^

305 was screened in Streptozotocin induced neonatal model of non-insulin dependent diabetes mellitus rats. Diabetes was induced by injecting Streptozotocin (100 mg kg^−1^) intraperitoneally into two days old pups. 305 administration for 3 weeks decreased blood glucose significantly, raised insulin, and improved insulin sensitivity level. 305 suppressed several inflammatory cytokines generation, protected against hyperlipidemia, and liver injury. 305 significantly restored pancreatic lipid peroxidation, catalase, superoxide dismutase, and reduced glutathione content. Histological studies of pancreatic tissues showed normal architecture after 305 administrations of diabetic rats. 305 successfully reduces blood glucose level, showing anti-oxidant, and anti-inflammatory effects. This leads to decreased histological damage in diabetic pancreatic tissues suggesting the possibility of future diabetes treatments.^109^

## 3 Conclusion

The escalating prevalence of metabolic complications, and the limited efficacy of current therapies underscore the urgent need of novel, and potent antidiabetic agents. Among heterocyclic scaffolds, thiazole has emerged as versatile, and biologically significant motif, highly valued for its synthetic accessibility, structural modifications, and promising pharmacological potential. Thus, the current review (2020–2025) consolidates recent advances in rational design, streamlined synthesis, *in vitro* anti-oxidant assay, antidiabetic evaluations, structure activity relationships, and computational docking analysis of thiazole-based heterocycles. Much emphasis is given to thiazole hybrids incorporating diverse pharmacophores, including hydrazine, azomethine, pyrazole, thiazolidinone, sulfonamide, imidazole, amide, hydrazide, indole, thiadiazole, oxadiazole, coumarin, thiophene, and pyrrole. Thiazole derivatives consistently demonstrated enhanced free radical scavenging potential in *in vitro* hydroxyl, ferric ion reducing, ABTS, and DPPH assays. These thiazoles also exhibited multi-target antidiabetic effects, notably *via* superior *in vitro* inhibitory potential of α-amylase, α-glucosidase, DPP-4, and aldose reductase, alongside selective modulation of PPAR-γ, which may improve insulin sensitivity, and metabolic regulation. SAR investigations identified structural motifs that enhance potency, and selectivity, while molecular docking studies revealed key interactions between thiazole core, and active-site residues of target proteins. Collectively, these computational, and experimental insights clarify mechanisms of action, and guide the optimization of lead compounds. To conclude, thiazole scaffolds, and their hybrid analogues represent promising platform for RONS-mediated antidiabetic drug discovery, particularly for addressing RONS-mediated pathways in DM. Therefore, continued medicinal chemistry efforts-integrating SARs, enzymology, and structure-based design are required to advance target-specific thiazole candidates with improved efficacy, and safety profiles.

## Author contributions

Dr Hamid Aziz: conceptualization, writing–original, and revised drafts.

## Conflicts of interest

Author declares no known potential competing financial interests or personal relationships that could have appeared to influence the research work reported in this review paper.

## Data Availability

This article is a review of previously published literature. No primary research results, software, or code have been included, and no new data were generated or analysed as part of this review.
